# Supplementary material for: Topography and soil variables drive the plant community distribution pattern and species richness in the Arjo-Diga forest in western Ethiopia
Source: PLoS One. 2024 Aug 6;19(8):e0307888. doi: 10.1371/journal.pone.0307888 (PMC11302921; doi:10.1371/journal.pone.0307888)
Supplement: S1 Table — (DOCX) [file pone.0307888.s001.docx]

|  | S1 Table. Floristic list of species in Arjo-Diga Forest.  (DOCX)  Growth Habit=Tree, SH=Shrub, H=Herb, Cl=Climber and *= endemic species | | | | |
| --- | --- | --- | --- | --- | --- |
| S/No | Scientific name | Family | Local name | Habit |  |
| 1 | *Abutilon figarianum Webb* | Malvaceae | Incinnii | H |  |
| 2 | *Abutilon longicuspe* *Hochst. ex A. Rich.* | Malvaceae | Hincini guracha | S |  |
| 3 | *Acemella caulirhiza* Del. | Asteraceae | Guticha | H |  |
| 4 | *Acacia abyssinica* Hochst.ex Benth | Fabaceae | Lafto | T |  |
| 5 | *Acalypha marissima* M.G.Gilbert*** | Euphorbiaceae | Gurgube qamalee | S |  |
| 6 | *Acanthus polystachius* Delile | Acanthaceae | Sokoru | S |  |
| 7 | *Achyranthes aspera* L. | Amaranthaceae | Maxxannee | H |  |
| 8 | *Achyrospermum schimperi* (Hochst. ex Briq.) Perkins | Lamiaceae | Mata bokkee | H |  |
| 9 | *Adiantum hispidulum* Sw | Adiantaceae | Gixoo | Fern |  |
| 10 | *Adiantum poiretii* Wikstr. | Adiantaceae |  | Fern |  |
| 11 | *Aeschynomene americana* L*.* | Fabaceae |  | H |  |
| 12 | *Aframomum alboviolaceum* (Ridl.) K.Schum | Zingiberaceae | Hogio | H |  |
| 13 | *Agelanthus dodoneifolius* (DC. )Polh.& Wiens | Loranthaceae |  | S |  |
| 14 | *Agratum conzoids* L*.* | Asteraceae | Udan awanesa | H |  |
| 15 | *Albiza grandibracteata* Taub | Fabaceae | Birbirii | T |  |
| 16 | *Albizia gummiffera* (J. F. Gmel.) C.A. Sm*.* | Fabaceae | Mukarbaa | T |  |
| 17 | *Albizia malacophylla* (A. Rich) Walp*.*Var*. ugandensis* | Fabaceae | Muke | T |  |
| 18 | *Allophylus abyssinicus* (Hochst.) Radik*.* | Sapindaceae | Molqoqoo | T |  |
| 19 | *Allophyylus macrobotrys* Gilg, | Sapindaceae | Gursadee | T |  |
| 20 | *Alysicarpus rugosus* (Willd.) DC *subsp. rugosus* | Fabaceae |  | H |  |
| 21 | *Ampelocissus schimperiana* (Hochst. exA. Rich.) Planch | Vitaceae | Naluu | Cl |  |
| 22 | *Antherotoma naudinii* Hook.f. | Melastomataceae |  | H |  |
| 23 | *Apodytes dimidiata* E. Mey. ex Am. | Icancinaceae | Wandabiyoo | T |  |
| 24 | *Athrixia rosmarinifolia* (Sch. Rip. ex Walp.) Olivo & Hiern | Asteraceae |  | H |  |
| 25 | *Bersama abyssinica* Fresen | Melianthaceae | Lolchiisaa | T |  |
| 26 | *Bidens macroptera* (Sch. Bip. ex Chiav.) Mesfin* | Asteraceae | Keloo | H |  |
| 27 | *Bidens ghedoensis* Mesfin* | Asteraceae | Keloo | H |  |
| 28 | *Bidens pilosa* L. | Asteraceae | Maxxannee | H |  |
| 29 | *Bridelia micrantha* (Hochst.) Baill*.* | Euphorbiaceae | Digaraba | S |  |
| 30 | *Bridelia scleroneura* Muell. Arg*.* | Euphorbiaceae | Muka saree | T |  |
| 31 | *Brillantaisia lamium* (Nees) Benth*.* | Acanthaceae |  | H |  |
| 32 | *Brucea antidysenterica* J.F.Mill. | Simaroubaceae | Qomonyoo | S |  |
| 33 | *Buddleja polystachya* Fresen*.* | Loganiaceae | Anfar | T |  |
| 34 | *Calpurnia aurea* (Ait.) Benth. | Fabaceae | Ceekaa | S |  |
| 35 | *Canthium oligocarpum* Hiern | Rubiaceae | Mixoo | S |  |
| 36 | *Capparis tomentosa* Lam. | Capparidaceae | Hrangamaa | S |  |
| 37 | *Cardamine trichocarpa* A. Rich | Brassicaceae |  | H |  |
| 38 | *Carissa spinarum* L. | Apocynaceae | Agamsa | S |  |
| 39 | *Cassipourea malosana* (Baker) Alston | Rhizophoraceae | Lokko bajii | T |  |
| 40 | *Cissus ruspolii* Gilg | Vitaceae | Hidaa reefa | Cl |  |
| 41 | *Celosia trigyna* L*.* | Amaranthaceae |  | H |  |
| 42 | *Celtis africana* Burm.f. | Ulmaceae | Cayii | T |  |
| 43 | *Centella asiatica* (L.) Urban | Apiaceae | Gurabaaloo | H |  |
| 44 | *Ceropegia glabripedicellata* De Wild | Asclepdaceae |  | H |  |
| 45 | *Chionanthus miwbraedii* (Gilg&Schelleab) Stearn | Oleaceae | Saxoo | T |  |
| 46 | *Chlorophytum gallabatense* Schweinf ex Baker | Anthericaceae |  | H |  |
| 47 | *Cissampelos pareira* L. | Menispermaceae | Hidda kalaalaa | Cl |  |
| 48 | *Citrus limon* (L) | Rutaceae | Lommi | S |  |
| 49 | *Clausena anisata* (Willd.) Benth. | Rutaceae | Ulumaayii | S |  |
| 50 | *Clematis hirsuta* Perr. & Guill | Ranunculaceae |  | Cl |  |
| 51 | *Clematis longicauda* Steud. ex A.Rich* | Ranunculaceae | Hida feetii | Cl |  |
| 52 | *Clematis simensis* Fresen. | Ranunculaceae | Hidaa feetii | Cl |  |
| 53 | *Clerodendrum myricoides* (Hochst.) Vatke | Lamiaceae | Marasissa | S |  |
| 54 | *Coffea arabica* L. | Rubiaceae | Buna | S |  |
| 55 | *Combretum collinum* Fresen. | Combretaceae | Dhanassa | T |  |
| 56 | *Combretum molle* R. Br. ex G. Don | Combretaceae | Adajabo | T |  |
| 57 | *Combretum paniculatum* Vent. | Combretaceae | Hidda baggee | T |  |
| 58 | *Commelina benghalensis* L. | Commelinaceae | Goroora | T |  |
| 59 | *Conyza sumatreosis* (Retz.) E.H. Walker | Asteraceae |  | H |  |
| 60 | *Cordia africana* Lam | Boraginaceae | Waddeessa | T |  |
| 61 | *Crassocephalum rubens* (Juss. ex Jacq.) S. Moore | Asteraceae |  | H |  |
| 62 | *Crepis foetida* L. | Asteraceae | Ananoo | H |  |
| 63 | *Crossoteryx febfebrifuga* (G. Don) Benth. | Rubiaceae | Qilimee | S |  |
| 64 | *Crotalaria lachnophora* Hochst. exA. Rich | Fabaceae |  | S |  |
| 65 | *Crotalaria rosenii* (Pox) Milne-Redh. ex Polhill**.* | Fabaceae | Gursaadee | H |  |
| 66 | *Crotalaria ononoides* Benth | Fabaceae |  | H |  |
| 67 | *Croton macrostachyus* Dell. | Euphorbiaceae | Bakanissa | T |  |
| 68 | *Cyathula prostrata* (L.) Blume var. *prostrata* | Amaranthaceae |  | H |  |
| 69 | *Cyathula uncinulata* (Schrad.) Schinz | Amaranthaceae | Dargu | H |  |
| 70 | *Cynodon nlemfuensis* Vanderyst | Poaceae | warrit | H |  |
| 71 | *Cynoglossum lanceolatum* Forssk | Boraginaceae |  | H |  |
| 72 | *Cyperus brevifolius* (Rottb.) Hasskn | Cyperaceae |  | H |  |
| 73 | *Cyperus fischerianus* A.Rich | Cyperaceae | Qunni | H |  |
| 74 | *Cyphostemma adenocaule*(Steud .ex A.Rich.) Desc. ex Wild &Drummond | Vitaceae | Hidda reefa | Cl |  |
| 75 | *Cyphostemma cyphopetalum (*Fresen.) Desc. ex Wild & Drummond | Vitaceae |  | H |  |
| 76 | *Dalbergia lactea* Vatke | Fabaceae | Amaltalo | S |  |
| 77 | *Digtaria abyssinica* (Hochst. ex A. Rich.) Stapf | Poaceae |  | H |  |
| 78 | *Dioscorea praehensilis* Benth. | Dioscoreaceae | Qochoo | C |  |
| 79 | *Diospyros abyssinica* (Hiern) F. White | Ebenaceae | Lokoo | T |  |
| 80 | *Diplolophium africanum* Turcz | Lamiaceae | Kamona dima | H |  |
| 81 | *Dodonea angustifolia* L. f. | Sapindaceae | Ittacha | S |  |
| 82 | *Dombeya torrida* (J.F.Gmel.) P. Bamps | Sterculiaceae | Daannisa | T |  |
| 83 | *Dracaena fragrans* (L.) Ker Gawl | Dracaenaceae | Afarfatu | S |  |
| 84 | *Dracaena steudneri* Engler | Dracaenaceae | afaraftu | T |  |
| 85 | *Drymaria cordata* (L.) Schultes | Caryophylllaceae |  | H |  |
| 86 | *Echinops giganteus* A. Rich* | Asteraceae | matabokkee | S |  |
| 87 | *Echinops longisetus* A. Rich*.* | Asteraceae | Qabaricho | S |  |
| 88 | *Ehretia cymosa* Thonn. | Boraginaceae | Ulaagaa | T |  |
| 89 | *Ekebergia capensis* Sparrm*.* | Meliaceae | Somboo | T |  |
| 90 | *Elephantopus mollis* Kunth | Asteraceae |  | H |  |
| 91 | *Eleusine indica* (L.) Gaertn | Fabaceae |  | H |  |
| 92 | *Entada abyssinica* Steud. ex A. Rich. | Fabaceae | Ambaltaa | T |  |
| 93 | *Erytherina abyssinica* Lam*. ex* DC | Fabaceae | walansu | T |  |
| 94 | *Erythrina burci* Schweinf | Euphorbiaceae | walensu | T |  |
| 95 | *Erythrococca trichogyne* (Muell.Arg.) Prain | Euphorbiaceae | Habacara | T |  |
| 96 | *Erythrophna septentrionalis* Verde | Sapindaceae | Damaee | T |  |
| 97 | *Euphorbia ampliphylla* Pax | Euphorbiaceae | Adaamii | S |  |
| 98 | *Euphorbia hirta* L. | Euphorbiaceae |  | H |  |
| 99 | *Euphorbia omariana* M .Gilbert* | Euphorbiaceae | Ribuquruphee | H |  |
| 100 | *Ficus exasperata* Vahl | Moraceae | Balansofi | T |  |
| 101 | *Ficus mucuso* Ficalho | Moraceae | Qilinxoo | T |  |
| 102 | *Ficus sur* Forssk. | Moraceae | Harbu | T |  |
| 103 | *Ficus sycomorus* L. | Moraceae | Odaa | T |  |
| 104 | *Ficus thonningii* Blume | Moraceae |  | T |  |
| 105 | *Ficus vasta* Forssk*.* | Moraceae | Qilxu | T |  |
| 106 | *Flacourtia indica* (Burm.f) Merr*.* | Flacourtiaceae | Akuukkuu | S |  |
| 107 | *Flueggea virosa* (Willd.) Voigt *subsp.virosa* | Euphorbiaceae | xaxessa | S |  |
| 108 | *Galinsoga parviflora* Cav | Asteraceae |  | H |  |
| 109 | *Gardenia ternifolia* Schumach. & Thonn | Rubiaceae | Gambeela | S |  |
| 110 | *Girardinia diversifolia* (Link) Friis | Urticaceae | Dobii | H |  |
| 111 | *Glinus lotoides* L. | Molluginaceae | wogret | H |  |
| 112 | *Gnidia glauca* (Fresen.) Gilg. | Thymelaeaceae | Qaqaroo | S |  |
| 113 | *Gouania longispicata* Engl | Rhamnaceae | Hida nalu | Cl |  |
| 114 | *Grewia bicolor* Juss. | Tiliaceae | Haroressa | S |  |
| 115 | *Grewia ferruginea* Hochst. ex A. Rich*.* | Tiliaceae | Dhoqonuu | S |  |
| 116 | *Guizotia scabra* (Vis.) Chiov*.* | Asteraceae | Tuffo | H |  |
| 117 | *Guizotia villosa* Sch. Bip* | Asteraceae | Hadaa | H |  |
| 118 | *Guizotia schimperi* Sch*. Bip. ex* Walp*.* | Asteraceae | Sichawo | H |  |
| 119 | *Haumaniastrum villosum* (Benth.) A.J. Paton | Lamiaceae |  | S |  |
| 120 | *Helinus mystacinus* (Ait.) E. Mey. ex Steud. | Rhamnaceae | Hoomachoo | H |  |
| 121 | *Hibiscus calyphyllus* Cavan. | Malvaceae |  | H |  |
| 122 | *Hibiscus noldeae* Bak.f | Malvaceae | Binto | S |  |
| 123 | *Hippocratea goetzei* Loes. | Celasteraceae | Hida gafarsa | Cl |  |
| 124 | *Hygrophila schulli* (Hamilt.) M.R. & S.M Almeida, | Acanthaceae | Matabokkee | H |  |
| 125 | *Hymenodictyon* floribundum (Hochst.&Steud.) Robinson | Rubiaceae | Gaarrii | T |  |
| 126 | *Hypericum quartinianum* A.Rich. | Guttiferae |  | S |  |
| 127 | *Hypoestes forskaolii* *(*Vahl*)* R.Br*.* | Acanthaceae |  | H |  |
| 128 | *Hypoestes triflora* (Forssk.) Roem. & Schult | Acanthaceae |  | H |  |
| 129 | *Impatiens hoschtetteri* Warb. | Balsaminaceae | Ansosila | H |  |
| 130 | *Indigofera arrecta* Hochst. Ex A. Rick | Fabaceae | Heennaa | S |  |
| 131 | *Indigofera spicata* Forssk* | Fabaceae |  | H |  |
| 132 | *Jasminum abyssinicum* Hochst. ex DC. | Oleaceae | Hidda Ichilbee | Cl |  |
| 133 | *Justicia schimperiana* (Hochst. ex Nees) T*.* Anders | Acanthaceae | Dumuga*,* | S |  |
| 134 | *Kalanchoe petitiana* A.Rich* | Crasulaceae | Bosoqqee | H |  |
| 135 | *Keetia gueinzii* (Sonn*.)* Bridson | Rubiaceae | Qosha aqmalee | S |  |
| 136 | *Kosteletzkya adoensis* (Hochst. ex A.Rich.) Mast | Malvaceae | Hincini | S |  |
| 137 | *Laggera crispata* (Vahl) Hepper & Wood | Asteraceae | Folii hayoo | H |  |
| 138 | *Landolphia buchananii* (Hall.f.) Stapf. | Apocynaceae | Hidda geeboo | Cl |  |
| 139 | *Lannea schimperi* (A. Rich.) Engl | Anacardiaceae | Daraku | T |  |
| 140 | *Lantana ukambensis* (Vatke) Verde | Verbenaceae | Mulu durba | S |  |
| 141 | *Leonotis ocymifolia* (Burm. f.) Iwarsson | Lamiaceae |  | S |  |
| 142 | *Lepisanthes senegalensis* (Juss.ex.poir) leenh | Sapindaceae | konoo | S |  |
| 143 | *Leucas martinicensis* (Jacq.) R.Br. | Lamiaceae |  | S |  |
| 144 | *Lippia adoensis* Hohst. ex Walp* | Verbenaceae | Kusaayee | S |  |
| 145 | *Macaranga capensis* (Baill*.)* Sim | Euphorbiaceae | allele | T |  |
| 146 | *Maesa lanceolata* Forssk | Myrsinaceae | Abayii | T |  |
| 147 | *Maytenus gracilipes* (Welw. ex 0liv.) Ecell | Celasteraceae | acaacii | T |  |
| 148 | *Maytenus obscura* (A. Rich.) Cu | Celasteraceae | kombolcha | T |  |
| 149 | *Maytenus undata* (Thumb.) Blakelock | Celasteraceae | qanqasha | S |  |
| 150 | *Momordica foetida* Schumach. | Cucubitaceae |  | CL |  |
| 151 | *Millettia ferruginea* (Hochst.) Bak* | Fabaceae | Sootalloo | T |  |
| 152 | *Mucuna melanocarpa* Hochst. ex A.Rich* | Fabaceae | machara | Cl |  |
| 153 | *Mukia maderaspatana* (L.) MJ. Roem. | Cucubitaceae |  | H |  |
| 154 | *Myrsine africana* L. | Myrsinaceae | Qacama | S |  |
| 155 | *Nicandra physaloides* (L.) Gaerin. | Solonaceae | Asangira | H |  |
| 156 | *Nuxia congesta* R.Br. ex Fresen. | Buddleiaceae | Qawwisa | T |  |
| 157 | *Ocimum urticifolium* Roth. | Lamiaceae | Ancabii addii | S |  |
| 158 | *Olea capensis subsp. macrocarpa* (c. H. Wright) Verde. in Bothalia | Oleaceae | Gagama | S |  |
| 159 | *Oplismenus hirtellus* (L.) P. Beauv. | Poaceae | marga gogoorii | H |  |
| 160 | *Otostegia tomentosa subsp.ambigens* A. Rich. | Lamiaceae | Tungit | S |  |
| 161 | *Oxyanthus speciosus subsp. stenocarpus* (K. Schum.)Bridson | Rubiaceae |  | S |  |
| 162 | *Oxytenanthera abyssinica* (A.Rich.) Munro | Poaceae | Shimala | H |  |
| 163 | *Paullinia pinnata* L. | Sapindaceae | Hidaa Dima | Cl |  |
| 164 | *Pavetta abyssinica* Fresen | Rubiaceae | Mixoo | S |  |
| 165 | *Pennisetum thumbergi* Kunt | Poaceae | Dafura | H |  |
| 166 | *Persicaria.nepalensis* (Meisn.) H.Gross | Polygalaceae |  | H |  |
| 167 | *Phoenix reclinata* Jacq. | Arecaceae | meexii | S |  |
| 168 | *Phyllanthus ovalifolius* Forssk | Euphorbiaceae |  | S |  |
| 169 | *Phytolacca dodecandra* L’ Herit. Li | Phytolaccaceae | Andoodee | S |  |
| 170 | *Piliostigma thonningii (*Schum*.)* Milne*-*Redh*.* | Fabaceae | Liluu | T |  |
| 171 | *Piper capense* L. f | Piperaceae | Tunjo | H |  |
| 172 | *Portulaca oleracea* L. | Portulacaceae |  | H |  |
| 173 | *Pouteria aolfi-friederici*( Engl)Baeh | Sapotaceae | qararoo | T |  |
| 174 | *Premna schimperi Engl* | Verbenaceae | Urgessa | S |  |
| 175 | *Prunus africana* (Hook. f.) Kalkm | Rosaceae | Hoomii | T |  |
| 176 | *Pteridium aquilinum* (L.) Kuhn | Athriaceae | Gixoo | Fern |  |
| 177 | *Pterolobium stellatum* (Forssk.) Brenan | Fabaceae | Harangama | S |  |
| 178 | *Pycnostachys abyssinica* Fresen*** | Lamiaceae |  |  |  |
| 179 | *Pycnostachys meyeri* Gurke | Lamiaceae |  | H |  |
| 180 | *Rhamnus prinoides* L’herit. | Rhamnaceae | Gesho | S |  |
| 181 | *Rihichssus tridentata*  (L. f.) Wild & Drummond | Vitaceae |  | Cl |  |
| 182 | *Rhus vulgaris* Meikle | Anacardiaceae | Xaxessa | S |  |
| 183 | *Rothmannra ureecelliformis* (Hiem) Bullock ex.Robym | Rubiaceae | Gadoo | T |  |
| 184 | *Rubus apetalus* Poir. | Rosaceae | Goraaa | S |  |
| 185 | *Rytigynia neglecta* (Hiem) Robyns | Rubiaceae | Mixoo | S |  |
| 186 | *Saba comonesis* (Bojer ex A.DC.) Pichon | Apocynaceae | Buru | C |  |
| 187 | *Salix mucronata* Willd. | Salicaceae | Alaltuu | T |  |
| 188 | *Sapium ellipticum* (Krauss) Pax. | Euphorbiaceae | Bosoqa | T |  |
| 189 | *Schrebera alata* (Hochst.)Welw. |  |  |  |  |
| 190 | *Schefflera abyssinica* (Hochst. ex Rich.) Harms | Araliaceae | Gatama | T |  |
| 191 | *Securidaca longepedunculata* Fresen. | Polygalaceae | Xamanaayii | T |  |
| 192 | *Senna occidentalis* (L)Link | Fabaceae |  | S |  |
| 193 | *Senna petersiana* (Bolle) Lock | Fabaceae | Ramso | S |  |
| 194 | *Sida rhombifolia* L. | Malvaceae | Karabaa | S |  |
| 195 | *Smilax anceps* Willd | Smilacaceae | Goraaa | Cl |  |
| 196 | *Sonchus bipontini* Asch. | Asteraceae |  | H |  |
| 197 | *Solanecio mannii* (Hook.) C. JefJrey | Asteraceae |  | H |  |
| 198 | *Solanecio angulantus* (Vahl) C. Jeffrey | Asteraceae |  | H |  |
| 199 | *Solanecio gigas* (Vatke) C. Jeffrey* | Asteraceae | Gomana shikoko | h |  |
| 200 | *Solanum anguivi* Lam | Solonaceae |  | S |  |
| 201 | *Solanum incanum* L*.* | Solonaceae | Hiddi | *S* |  |
| 202 | *Sphaeranthus suaveolens var.sphaeranthus suaveolens* (Forssk.) Dc | Asteraceae | Rashad | H |  |
| 203 | *Stephania abyssinica* (Qurt, Dill & A. Rich.)Walp | Menispermaceae | Hida kalal | Cl |  |
| 204 | *Stereospermum kunthianum* Cham. | Bignoniaceae | Botoroo | T |  |
| 205 | *Syzgium guineese (Willd.) DC.subsp. guineense* | Myrtaceae | Gossu | T |  |
| 206 | *Syzygium guineense*(Wild.)DC. *subsp. afromontanum* F.White | Myrtaceae | Badessa | T |  |
| 207 | *Tagestes minuta* L. | Asteraceae | Hurgoftuu | H |  |
| 208 | *Teclea nobilis* Del. | Rutaceae | Hadheessa | T |  |
| 209 | *Terminalia macroptera* Guill & Perr. | Combretaceae | Dabaqqaa | T |  |
| 210 | *Thalictrum rhynchocarpum* Dilland A.Rich | Ranunculaceae |  | h |  |
| 211 | *Tragia brevipes* Pax | Euphorbiaceae |  | H |  |
| 212 | *Tragia doryodes* M.Gilbert* | Euphorbiaceae | Gurgubbee | H |  |
| 213 | *Trema orientalis* (L) BI | Ulmaceae |  | T |  |
| 214 | *Trichilia dregeana* Sond. | Meliaceae | Ununuu | T |  |
| 215 | *Trichilia emetica* Vahl | Meliaceae | Qolaxii | T |  |
| 216 | *Trifolium rueppellianum* Fresen | Fabaceae | Sidissa | H |  |
| 217 | *Tristemma mauritianum* J.F Gmelin | Melastomataceae |  | H |  |
| 218 | *Triumfetta rhomboidca* Jaeq*.* | Tiliaceae | Hincini | H |  |
| 219 | *Urera hypselodendron* (A.Rich.) Wedd | Urticaceae | Lanqeessaa | Cl |  |
| 220 | *Vangueria apiculata* K. Schum | Rubiaceae | Mixoo | S |  |
| 221 | *Vepris dainellii* (Pichi-Serm.) Kokwaro* | Rutaceae | Hadessa | S |  |
| 222 | *Vernonia ischnophylia* Musch | Asteraceae | Soyoma haree | S |  |
| 223 | *Vernonia amygdalina* Del. | Asteraceae | Eebicha | T |  |
| 224 | *Vernonia auriculifera* Hiern | Asteraceae | Reejjii | S |  |
| 225 | *Vernonia hymenolepis* A. Rich | Asteraceae | Soyoma boyee | S |  |
| 226 | *Vernonia leopoldi* (Sch.Bip.ex Walp)Vatke *** | Asteraceae | Soyoma | S |  |
| 227 | *Vernonia theophrastifolia* Schweinf. ex Oliv. & Hiern | Asteraceae |  | S |  |
| 228 | *Veronica javanica* Blume*.* | Scrophulariaceae |  | H |  |
| 229 | *Vitex doniana* Sweet | Verbenaceae | Qoqora | T |  |
| 230 | *Warburgia ugandensis* Sprague | [Canellaceae](https://en.wikipedia.org/wiki/Canellaceae) | Befti | T |  |
| 231 | *Xanthium strumarium* L | Asteraceae |  | H |  |
| 232 | *Ximenia americana* L. | Olacaceae | Hudhaa | H |  |
| 233 | *Zehneria scabra* (Linn.f.) Sond. | Cucrbitaceae | Hidarefa | Cl |  |
| 234 | *Ziziphus spina-christi* (L.)Desf. | Rhamnaceae | Qoqora | T |  |
