# Supplementary material for: Topography and soil variables drive the plant community distribution pattern and species richness in the Arjo-Diga forest in western Ethiopia
Source: PLoS One. 2024 Aug 6;19(8):e0307888. doi: 10.1371/journal.pone.0307888 (PMC11302921; doi:10.1371/journal.pone.0307888)
Supplement: S2 Table — (DOCX) [file pone.0307888.s002.docx]

S2 Table. Value of the indicator species in identified plant communities and their significant p value.

| Indicator species | Community types | Indicator value | P value |
| --- | --- | --- | --- |
| *Ehretia cymosa* | 1 | 0.603 | 0.001*** |
| *Erythrococca trichogyne* | 1 | 0.254 | 0.022* |
| *Euphorbia ampliphylla* | 1 | 0.364 | 0.003* |
| *Pruns africana* | 1 | 0.353 | 0.002* |
| *Tecela noblis* | 1 | 0.202 | 0.05* |
| *Bersama abyssinica* | 2 | 0.284 | 0.033* |
| *Cyathula cylindrica* | 2 | 0.217 | 0.026* |
| *Flacourtia indica* | 2 | 0.282 | 0.04* |
| *Trichilia dregeana* | 2 | 0.551 | 0.004* |
| *Acaccia abyssinica* | 3 | 0.874 | 0.001*** |
| *Buddleja polystachya* | 3 | 0.24 | 0.022* |
| *Girardinia diversifolia* | 3 | 0.218 | 0.025* |
| *Grewia bicolar* | 3 | 0.366 | 0.003* |
| *Lippia adoensis* | 3 | 0.208 | 0.029* |
| *Millettia ferruginea* | 3 | 0.22 | 0.055* |
| *Vernonia auriculifera* | 3 | 0.279 | 0.032* |
| *Combertum collinum* | 4 | 0.276 | 0.044* |
| *Gnidia glauca* | 4 | 0.229 | 0.021* |
| *Maytenus obscura* | 4 | 0.568 | 0.003* |
| *Guizotia villosa* | 4 | 0.164 | 0.049* |
| *Combretum molle* | 5 | 0.262 | 0.01* |
| *Ficus exasperata* | 5 | 0.376 | 0.003* |
| *Ficus vasta* | 5 | 0.276 | 0.015* |
| *Flueggea virosa* | 5 | 0.425 | 0.002* |
| *Gardenia ternifolia* | 5 | 0.563 | 0.001*** |
| *Grewia ferruginea* | 5 | 0.203 | 0.055* |
| *Piliostigma thonningii* | 5 | 0.704 | 0.001*** |
| *Stereospermum kunthianum* | 5 | 0.341 | 0.005** |
| *Syzygium guineense.subsp.afromontanum* | 5 | 0.446 | 0.001*** |
| *Terminalia macroptera* | 5 | 0.599 | 0.001*** |

Significance codes * = (p < 0.5), ** = (p < 0.01), *** = (p < 0.001)
