# Supplementary material for: Topography and soil variables drive the plant community distribution pattern and species richness in the Arjo-Diga forest in western Ethiopia
Source: PLoS One. 2024 Aug 6;19(8):e0307888. doi: 10.1371/journal.pone.0307888 (PMC11302921; doi:10.1371/journal.pone.0307888)
Supplement: Attachment — Submitted filename: PONE-D-23-07720.pdf [file pone.0307888.s003.pdf]

PLOS ONE

Topography factors and soil variables drive the plant community distribution pattern and species richness in the Arjo Diga forest in western Ethiopia.

--Manuscript Draft--

Manuscript Number:	PONE-D-23-07720
Article Type:	Research Article
Full Title:	Topography factors and soil variables drive the plant community distribution pattern and species richness in the Arjo Diga forest in western Ethiopia.
Short Title:	Topography and soil drive plant community distribution pattern in the Arjo Diga forest western Ethiopia
Corresponding Author:	Tariku Teaw berihun, msc<br>Dilla University<br>Addis Ababa, ETHIOPIA
Keywords:	environmental variables, species diversity, plant communities
Abstract:	Studies on, species diversity and plant communities along environmental gradients are some of the bases for designing forest management plans. Therefore, this study focused on floristic diversity and the relationship between plant diversity, plant community and environmental variables in the Arjo-Diga forest. For the current study, seventy-two quadrats (30 m x 30 m (900 m2) were laid along altitudinal drops to record vegetation data. All trees and shrubs with a DBH of 2.5 cm and a height of 3 m in each plot were used for data enumeration. Herb and soil data were collected from five subplots of 1 m × 1 m (1 m2) laid at the four corners and center of each quadrat. The diversity indices (i.e. richness, diversity, and evenness) were computed using R.4.2 software. Sorensen's similarity coefficient was used to calculate the similarity between community types. The vegetation classification into community types was calculated using agglomerative hierarchical cluster analysis using R.4.2 software. Relationships between plant community types and environmental variables were analyzed using canonical correspondence analysis (CCA). A total of 234 species belonging to 183 genera and 73 families were collected and identified. The three dominant species-rich families were Asteraceae (29 species), followed by Fabaceae (23 species) and Euphorbiaceae (14 species). Of these, 61 species (26%) were trees, 70 species (30%) were shrubs, 84 species (36%) were herbs, and 19 species (8%) were climbers. Twenty (8.5%) species are endemic to Ethiopia and Eritrea flora. Five plant communities were identified: Maesa lanceolata – Ehretia cymosa, Trichilia dregeana – Flacourtia indica, Acacia abyssinica – Millettia ferruginea, Combertum collinum – Croton macrostachyus and Terminalia macroptera – Piliostigma thonninigii. Community type 4 showed the highest Shannon diversity (4.3) compared to the other communities. A high coefficient of similarity was observed between community types 2 and 4. The CCA ordination showed that the cumulative percentage of variance explained by the first two CCA axes was 72.3% of the variation in the species data. In addition, the CCA ordination diagram showed that plant community type 4 was explained by disturbance in CCA axis 1 and communities 1, 2, 3 and 5 by CEC, TN and altitude in CCA axis 2. Altitude and disturbance are negatively correlated with species richness and evenness. In summary, the Arjo Diga forest contained high species richness, great diversity and endemic species. However, anthropogenic factors such as man-made fires, human settlements, the expansion of farmland, deforestation and cattle grazing have a significant impact on species diversity and plant community formation. However, anthropogenic factors such as human-induced fire, human settlement and farmland expansion, forest clearing, and livestock grazing have significant impacts on species diversity and plant community formation. It is therefore an urgent task to create sustainable plans for the conservation of biodiversity and endemic species in forests.
Order of Authors:	Tariku Teaw berihun, msc
	Zemede Asfaw Woldemariam, Prof.
	Tamrat bekele GODE, prof
	Ermias Luleka1 Molla, associate prof.

Powered by Editorial Manager® and ProduXion Manager® from Aries Systems Corporation

Additional Information:	
**Question**	**Response**
**Financial Disclosure**<br><br>Enter a financial disclosure statement that describes the sources of funding for the work included in this submission. Review the submission guidelines for detailed requirements. View published research articles from *PLOS ONE* for specific examples.<br><br>This statement is required for submission and **will appear in the published article** if the submission is accepted. Please make sure it is accurate.<br><br>**Unfunded studies**<br>Enter: *The author(s) received no specific funding for this work.*<br><br>**Funded studies**<br>Enter a statement with the following details:<br>• Initials of the authors who received each award<br>• Grant numbers awarded to each author<br>• The full name of each funder<br>• URL of each funder website<br>• Did the sponsors or funders play any role in the study design, data collection and analysis, decision to publish, or preparation of the manuscript?<br>• **NO** - Include this sentence at the end of your statement: *The funders had no role in study design, data collection and analysis, decision to publish, or preparation of the manuscript.*<br>• **YES** - Specify the role(s) played.<br><br>* typeset	The author(s) received no specific funding for this work.
**Competing Interests**<br><br>Use the instructions below to enter a competing interest statement for this submission. On behalf of all authors, disclose any competing interests that could be perceived to bias this work—acknowledging all financial support and any other relevant financial or non-financial competing interests.	The authors have declared that no competing interests exist.

This statement is required for submission and **will appear in the published article** if the submission is accepted. Please make sure it is accurate and that any funding sources listed in your Funding Information later in the submission form are also declared in your Financial Disclosure statement.<br><br>View published research articles from *PLOS ONE* for specific examples.<br><br>**NO authors have competing interests**<br><br>Enter: *The authors have declared that no competing interests exist.*<br><br>**Authors with competing interests**<br><br>Enter competing interest details beginning with this statement:<br><br>*I have read the journal's policy and the authors of this manuscript have the following competing interests: [insert competing interests here]*<br><br>* typeset	
**Ethics Statement**<br><br>Enter an ethics statement for this submission. This statement is required if the study involved:<br><br>• Human participants<br>• Human specimens or tissue<br>• Vertebrate animals or cephalopods<br>• Vertebrate embryos or tissues<br>• Field research<br><br>Write "N/A" if the submission does not require an ethics statement.<br><br>General guidance is provided below. Consult the submission guidelines for detailed instructions. **Make sure that all information entered here is included in the**	"N/A" if the submission does not require an ethics statement.

**Methods section of the manuscript.**<br><br>**Format for specific study types**<br><br>**Human Subject Research (involving human participants and/or tissue)**<br>• Give the name of the institutional review board or ethics committee that approved the study<br>• Include the approval number and/or a statement indicating approval of this research<br>• Indicate the form of consent obtained (written/oral) or the reason that consent was not obtained (e.g. the data were analyzed anonymously)<br><br>**Animal Research (involving vertebrate animals, embryos or tissues)**<br>• Provide the name of the Institutional Animal Care and Use Committee (IACUC) or other relevant ethics board that reviewed the study protocol, and indicate whether they approved this research or granted a formal waiver of ethical approval<br>• Include an approval number if one was obtained<br>• If the study involved *non-human primates*, add *additional details* about animal welfare and steps taken to ameliorate suffering<br>• If anesthesia, euthanasia, or any kind of animal sacrifice is part of the study, include briefly which substances and/or methods were applied<br><br>**Field Research**<br><br>Include the following details if this study involves the collection of plant, animal, or other materials from a natural setting:<br>• Field permit number<br>• Name of the institution or relevant body that granted permission	
**Data Availability**<br><br>Authors are required to make all data underlying the findings described fully available, without restriction, and from the time of publication. PLOS allows rare exceptions to address legal and ethical concerns. See the PLOS Data Policy and FAQ for detailed information.	Yes - all data are fully available without restriction

Powered by Editorial Manager® and ProduXion Manager® from Aries Systems Corporation

A Data Availability Statement describing where the data can be found is required at submission. Your answers to this question constitute the Data Availability Statement and **will be published in the article**, if accepted.<br><br>**Important:** Stating 'data available on request from the author' is not sufficient. If your data are only available upon request, select 'No' for the first question and explain your exceptional situation in the text box.<br><br>Do the authors confirm that all data underlying the findings described in their manuscript are fully available without restriction?	
**Describe where the data may be found in full sentences. If you are copying our sample text, replace any instances of XXX with the appropriate details.**<br><br>• If the data are **held or will be held in a public repository**, include URLs, accession numbers or DOIs. If this information will only be available after acceptance, indicate this by ticking the box below. For example: *All XXX files are available from the XXX database (accession number(s) XXX, XXX.*).<br>• If the data are all contained **within the manuscript and/or Supporting Information files**, enter the following: *All relevant data are within the manuscript and its Supporting Information files.*<br>• If neither of these applies but you are able to provide **details of access elsewhere**, with or without limitations, please do so. For example:<br><br>*Data cannot be shared publicly because of [XXX]. Data are available from the XXX Institutional Data Access / Ethics Committee (contact via XXX) for researchers who meet the criteria for access to confidential data.*<br><br>*The data underlying the results*	All relevant data are within the manuscript and its Supporting Information files.

presented in the study are available from (include the name of the third party and contact information or URL).<br>• This text is appropriate if the data are owned by a third party and authors do not have permission to share the data.<br><br>* typeset	
Additional data availability information:	Tick here if the URLs/accession numbers/DOIs will be available only after acceptance of the manuscript for publication so that we can ensure their inclusion before publication.

Click here to access/download;Manuscript;submitted T+E+Z+tamirat.doc

Topography factors and soil variables drive the plant community distribution pattern and species richness in the Arjo Diga forest in western Ethiopia.

Tariku Berihun[1], Ermias Luleka[1], Tamrat Bekele[1] and Zemede Asfaw[2]1

[1]Department of Biology, Dilla University, Dilla Ethiopia

[1&2]Department of Plant Biology and Biodiversity Management, Addis Ababa University, Addis Ababa, Ethiopia

E-mail:berihun.tariku@yahoo.com2,ermias.lulekalm@aau.edu.et2,tambek07@gmail.com1, zemede.asfaw@aau.edu.et1

Corresponding Author. berihun.tariku@yahoo.com

Abstract

*Studies on, species diversity and plant communities along environmental gradients are some of the bases for designing forest management plans. Therefore, this study focused on floristic diversity and the relationship between plant diversity, plant community and environmental variables in the Arjo-Diga forest. For the current study, seventy-two quadrats (30 m x 30 m (900 m2) were laid along altitudinal drops to record vegetation data. All trees and shrubs with a DBH of 2.5 cm and a height of 3 m in each plot were used for data enumeration. Herb and soil data were collected from five subplots of 1 m × 1 m (1 m^2) laid at the four corners and center of each quadrat. The diversity indices (*i.e. *richness, diversity, and evenness) were computed using R.4.2 software. Sorensen's similarity coefficient was used to calculate the similarity between community types. The vegetation classification into community types was calculated using agglomerative hierarchical cluster analysis using R.4.2 software. Relationships between plant community types and environmental variables were analyzed using canonical correspondence analysis (CCA). A total of 234 species belonging to 183 genera and 73 families were collected and identified. The three dominant species-rich families were Asteraceae (29 species), followed by Fabaceae*

(23 species) and Euphorbiaceae (14 species). Of these, 61 species (26%) were trees, 70 species (30%) were shrubs, 84 species (36%) were herbs, and 19 species (8%) were climbers. Twenty (8.5%) species are endemic to Ethiopia and Eritrea flora. Five plant communities were identified: Maesa lanceolata – Ehretia cymosa, Trichilia dregeana – Flacourtia indica, Acacia abyssinica – Millettia ferruginea, Combertum collinum – Croton macrostachyus and Terminalia macroptera – Piliostigma thonninigii. Community type 4 showed the highest Shannon diversity (4.3) compared to the other communities. A high coefficient of similarity was observed between community types 2 and 4. The CCA ordination showed that the cumulative percentage of variance explained by the first two CCA axes was 72.3% of the variation in the species data. In addition, the CCA ordination diagram showed that plant community type 4 was explained by disturbance in CCA axis 1 and communities 1, 2, 3 and 5 by CEC, TN and altitude in CCA axis 2. Altitude and disturbance are negatively correlated with species richness and evenness. In summary, the Arjo Diga forest contained high species richness, great diversity and endemic species. However, anthropogenic factors such as man-made fires, human settlements, the expansion of farmland, deforestation and cattle grazing have a significant impact on species diversity and plant community formation. However, anthropogenic factors such as human-induced fire, human settlement and farmland expansion, forest clearing, and livestock grazing have significant impacts on species diversity and plant community formation. It is therefore an urgent task to create sustainable plans for the conservation of biodiversity and endemic species in forests.

Keywords: environmental variables, species diversity, plant communities

Introduction

Investigating the relationship between vegetation attributes (i.e., richness, diversity and evenness) and environmental variables is critical to designing conservation ecology [1]. The

interactions of various environmental factors can also lead to changes in habitat conditions. Therefore, environmental factors directly or indirectly affect species distribution and plant diversity. The composition, diversity and spatial distribution pattern of plant species are influenced by abiotic and biotic factors [2]. Plant and abiotic factors are in complex relationships, and many factors interact with each other. Therefore, research into the influence of abiotic factors on species distribution and plant community formation is urgently needed[3].

Most studies of environmental factors have focused on elevation, topography, and soil to study how environmental factors affect species composition and plant diversity in different ecosystems. In particular, elevation drastically alters abiotic factors such as water, temperature, soil composition and area, which in turn affect plant growth and development [4, 5]. Furthermore, elevation regulates the-response of plant communities to environmental variables at the regional/global scale [6]. A study by Pandey, Adhikari [7] found different patterns of species richness along elevation gradients. Several studies have shown that species richness patterns monotonically decrease from the lowest to highest elevations[8, 9] or monotonically increase (Baruch, 198). Studies have also shown hump-shaped patterns at mid-elevations[2, 10] However, some researchers have shown that species richness is low at mid-elevations [11]. This suggests that species richness along elevation gradients was not a general trend since many species show different phenotypic traits, such as different leaf characteristics, biomass, and phenology, along elevation gradients. As a result, patterns of plant species richness and distribution along elevation gradients often differ in ways that are important to conservationists.

Studies have also shown that soil nutrients play a crucial role in changing vegetation structure and plant diversity by creating heterogeneous habitats. In tropical forests, soil nutrient deficiencies have been reported to affect forest community structure, plant biomass, tree height and basal area [12]. On the other hand, forests with a high species richness at locations show high nutrient dynamics. The effects of soil TN and available phosphorus on plant community structure seemed clear [13]. Soil TN is a limiting factor for plant growth [14] and affects plant diversity and community composition. Other studies have suggested that species richness changes during high nitrogen deposition [15]. Soil CEC in tropical forests affects tree species richness [16, 17]. However, there are still knowledge gaps in explaining how soil CEC affects tree species richness in tropical forests.

Plant species composition, diversity, and distribution are also affected by anthropogenic disturbances such as agricultural expansion, settlement, livestock overgrazing, selective deforestation, and fire [18]. Disturbance has a positive effect on vegetation properties to a certain extent[19]. However, disturbance mostly has a negative impact on vegetation; as the relationship between vegetation attributes and disturbance is often negative [20, 21]. Under conditions of severe disturbance, species richness and diversity are low because most species cannot tolerate frequent destructive events. However, due to dominant competitors and fast colonizers, high species richness can be predicted at intermediate levels of disturbance. Therefore, understanding species responses to anthropogenic disturbances is important to enable effective conservation decisions, for example, through information on habitat protection and restoration to maintain important ecological phenomena such as species range thresholds.

Different authors have documented the results of studies on species diversity, structure, and regeneration status in different moist Afromontane forests in different parts of Ethiopia. However, many of their species are now threatened, endangered, or locally extinct due to habitat destruction, fragmentation, and overexploitation of forest products and habitats [22]. Understanding the interaction between species diversity and composition and the relationship between environmental factors and plant community types remains critical to the development of forest management strategies.

The Arjo Diga Forest was declared in 2014 as a biodiversity conservation incentive. Although the forest is under protection, it has been continuously exploited by the surrounding people for the expansion of agricultural land, timber, firewood gathering and charcoal production, and other purposes. There have been no previous studies of species composition, structure, or plant community analysis in these areas. The present study focuses on community analysis along environmental gradients and seeks to assess the impact of environmental factors on plant diversity (i.e., richness, diversity and evenness). Specifically, the objectives of this study were to (1) examine the floristic composition and diversity of the Arjo-Diga forest, (2) examine the impact of environmental variables on the plant diversity attributes (richness, diversity, and evenness), and (3) explore the relationship between plant community types and environmental variables.

Materials and methods

Study area

The Arjo-Diga forests are located in southwestern Ethiopia and cover a total area of 12,683.6 hectares with elevations ranging from 1,200 to 2,220 meters above sea level (Diga District Agricultural Office, 20017). The forest is located in Diga District, East Wollega Zone, Oromia Regional State, 12 km from Nekemte town, and 340 km from Addis Ababa (capital of Ethiopia). Geographically located at 9°59′00″ and 9°6′30″ north and 36°18′30″ and 36°24′30″ east (Fig. 1). It is bounded by three districts: Guto Gida, Chewaka, Sasiga, and Leka Dulecha. The topography is variable and ranges from flat, gentle slope, moderate slope, to steep slope. Slopes are 7-30% in the southwest and 30-60% in the northeast, so the area is more prone to intense erosion. According [Jaleta and Jebssa [23]] the study area has two agroecological zones: the lowlands (51.4%) and the midlands (48.6%). The midlands are steep and dominantly covered by forest.

Soil and Geology

Acrisols are the dominant soil [24] and heavily weathered acidic soil with low CEC. In addition, these soils have a low pH value and too little phosphorus. The dominant soil color was red in the midlands and black in the lowlands. Geologically, the study area features Precambrian rocks of high origin and migmatites [25].

Climate

The study area mainly experiences tepid to cool subhumid mid-highlands in the northeastern part and hot to warm humid lowlands in the northwest. The maximum temperature was 25.7°C, and the minimum temperature was 10.2°C (Fig. 2). The mean annual precipitation is approximately 19977 mm/year with a unimodal precipitation pattern. The monthly mean precipitation trend shows its maximum in July, June and August and is dry from November and extends into January.

Vegetation types

According to Friis et al. (2010), the Arjo-Diga forest vegetation type is a part of the moist Afromontane Forest and is characterized by broadleaf evergreen plant species. This vegetation is found at an altitude between 1500 and 2342 meters above sea level with characteristic emergent species, including *Pouteria adolf-friederici*. Other distinctive species in this forest are *Sapium ellipticum, Celtis africana, Euphorbia ampliphylla, Ficus sur, Schefflera abyssinica, Syzygium guineense ssp. afromontanum, Nuxia congesta, Galiniera saxifraga, Rytigynia Neglecta, Vepris dainellii, Rothmannia urcelliformi* and several *Albizia* species (Friis et al., 2010). *Combretum paniculatum, Landolphia buchananii and Urera hypselodendron* were the dominant woody climbers in the forest. The western part of the study area borders the Didessa Valley, which formed the Combretum-Terminalia vegetation

Methods of data collection

Vegetation data

A reconnaissance survey was conducted from October 2010 to January 2013 to select sampling sites and to decide on the possible number of transect lines to be laid down. Following a reconnaissance survey, nine transect lines were systematically laid along an elevation gradient from 1360 to 2220 m above sea level from the crest of the mountain to the base. Vegetation data are collected from a total of 72 quadrats along nine transects that are systematically constructed using the sampling approach described by [26]. Each plot was relocated every 100 m elevation along the transect lines. A plot 30 m x 30 m (900 m^2) and 5 m x 5 m (25 m^2) were laid out for the counting tree and shrub species. Herbaceous species are recorded within a 2 m x m 2 m (4 m2) subplot in the main plot, one in the center and four from each corner of the plot. Finally, the percentage cover of all wood species in each plot was estimated visually and later converted to a

1–9 modified Braun-Blanquet scale [27]. Samples in each plot were collected, pressed and dried for identification at the National Herbarium (ETH), Department of Plant Biology and Biodiversity Management Addis Ababa University. The plant species were identified by reference to the Flora of Ethiopia and Eritrea (volumes 1-7) and confirmed with authentic specimens housed in the National Herbarium of Ethiopia. All authenticated specimens have been deposited with the National Herbarium, ETH and Addis Ababa University.

Environmental Data

The elevation, geographic coordinates, and slope for each of the 72 quadrats were recorded using a handheld Garmin GPS H72 and clinometer, respectively. Soil samples were collected from 72 plots with a soil core sampler at a depth of 30 cm within 1 m x1 m $^{(1\ m2}$) subplots, one in the center and the other four at each corner of each sample quadrat. A total of 72 composite soil samples, each weighing approximately 1 kilogram, were taken to the Bedele soil laboratory. Samples were air dried, ground and passed through a 2 mm sieve to remove rock fragments and large root particles prior to analysis. Soil samples were analyzed at Bedele's soil laboratory for selected soil for selected soil chemistry properties, including pH, organic carbon (C_{org}), available phosphorus, total nitrogen (N_t) and cation exchange capacity (CEC). Soil organ carbon ($C_{org)}$ was determined by the dichromate oxidation method [28], and total soil nitrogen was analyzed by the Kjeldahl digestion and distillation method [29]. Soil pH was determined using a pH meter at a soil to liquid ratio of 1:2.5[30], available phosphorus was extracted by the Bray-1 method [31] and measured using a spectrophotometer, and cation exchange capacity (CEC) was determined using the following ammonium acetate method [32].

Furthermore, the ecological disturbance of each plot of the Arjo Diga forest by human disturbances, such as selective logging of wood species for firewood, charcoal production, and agricultural expansion, was subjectively assessed according to [33-35] and subjective scales were denoted as 0 = zero. 1=low, 2=moderate and 3=heavy.

Data analysis

Plant community classification

In this study, hierarchical agglomerative clustering analysis using R- 4.2.2 was performed to classify vegetation into community types. Wards' method was applied for cluster analysis to minimize variance within the clusters. Plant community types identified from the cluster analysis were further refined in a synoptic table in which species occurrences were summarized as synoptic cover-abundance values [27]. Two characteristic species with high synoptic cover-abundance values were used to name plant community types [36, 37]. Additionally, plant communities were named using indicator species analysis [38]. An indicator value was calculated and tested for significance using permutation tests for each grouped species (99 iterations). In this study, a species was considered an indicator of a group if its indicator value was significantly higher at $P < 0.05$.

Species diversity: The Shannon diversity index and Shannon's evenness were calculated to describe the species diversity, richness and evenness of the study area and plant communities using R software version 4.2.2(2022-10-31 ucrt). The Shannon–Wiener diversity index (H') is calculated using Equation (1):

$$H' = -\sum_{i=1}^{s} \mathrm{Pi} \ln \mathrm{Pi} \; (\mathrm{Eq.}\,1)$$

where H'= Shannon–Wiener diversity index; S=number of species, pi=proportion of the ith species and ln= natural logarithm of each pi value. The value of the Shannon diversity index is usually between 1.5 and 3.5 and rarely exceeds 4.5 when all species are represented by the same number of individuals[36]. Shannon's evenness index (E) was also calculated from the ratio of observed diversity to maximum diversity using Equation (2).

$$E = \frac{\mathrm{H}'}{\ln(\mathrm{S})} = \frac{H'}{\mathrm{H}'\mathrm{max}} \text{(Eq. 2)}$$

where Hmax = the maximum level of diversity within a given population, which is equal to ln (number of species). Values of E typically range from 0 to 1, with 1 representing a situation where all species are equally abundant [39]. Sørensen's similarity coefficient has been used to compare floristic similarity between two communities [36, 39]. It is often used because it gives more weight to species common to the quadrats rather than to species common to only one of the two sample quadrats. Sorensen's similarity coefficient (Ss) is calculated using Equation (3):

$$S_s = \frac{2a}{2a+b+c} \text{(Eq.3)}$$

where Ss = Sorensen's similarity coefficient, a = the number of species common to both samples, b = the number of species present in one of the samples being compared, and c = the number of species present in the other sample available. Sorensen similarity coefficient values range from 0 (complete dissimilarity) to 1 (complete similarity).

Ordination:

Ordination is a multivariate analysis that clearly shows the relationships among species, plots, plant communities, and environmental variables in a two-dimensional space using an ordination diagram[36]. In this study, detrended correspondence analysis (DCA) of the species data revealed that the longest first-axis gradient length was 4.2 SD (standard deviation units),

suggesting that canonical correspondence analysis (CCA) was appropriate. CCA is calculated using the CCA function and tested for significance using the ANOVA function. Forward and Monte Carlo permutation tests (99) were used to select variables that significantly ($P<0.05$) explained the variance in species composition. A total of 72 quadrats, 234 species and 8 environmental variables (elevation, slope, pH, available phosphorus, TN, CEC disturbance and SOC) were included in this analysis. In addition, the relationships between environmental variables and species diversity and richness were tested using a multiple linear regression model, which was significant at $p<0.05$. Pearson's correlation coefficient is calculated to find correlations between two environmental variables using SPSS version 20 windows.

Results

Floristic composition

A total of 234 species belonging to 183 genera and 73 families were recorded and documented in this study. The five dominant families with high species richness were Asteraceae 29 species, Fabaceae 23, Euphorbiaceae 14, Lamiaceae 12 and Rubiaceae 11 species. On the other hand, 8 families had between six and four species each (Table 1). Trees, shrubs, herbs and climbers comprised 61 (26%), 70 (30%), 84 (36%) and 19 (8%) species, respectively (Figure 3). Of these, 20 (8.5%) species are endemic to the flora of Ethiopia and Eritrea. Species such as *Acalypha marissima*, *Bidens ghedoensis*, *Bidens macroptera*, *Erythrina burci*, *Echinops longisetus*, *Crotalaria rosenii*, *Vepris dainellii* and *Vernonia leopoldi* were some of the endemic species in the floristic region of Wollega. In addition, there are approximately 13 (5.5%) species belonging to 10 families and 13 genera listed as a new record in the Wollega floral region (Table 2).

Table 1. Families with more than three genera and their representative number of species

Family (N=73)	No of genera (No=183)	No of species (N=234)
Asteraceae	17(10%)	29(12.4%)
Fabaceae	16(8.7%)	23 (9.8%)
Rubiaceae	11(7%)	11 (4.7%)
Euphorbiaceae	10(5.5%)	14 (6%)
Lamiaceae	10(8.7%)	12(5.1%)
Acanthaceae	6 (2.9%)	6(2.6%)
Poaceae	6(2.9%)	6(2.6%)
Malvaceae	4(2.2%)	5(2.1%)
Rhamnaceae	4(2.2%)	5(2.1%)
Rutaceae	4(2.2%)	4(1.7%)
Sapindaceae	4(2.2%)	6(2.6%)
Verbenaceae	4(2.2%)	4(1.7%)
Vitaceae	4(2.2%)	5(2.1%)

Table 2. New records of species in the Wollega floristic region,

NO	Species name	Family	Ha.	Floristic Region
**1**	*Crepis foetida*	Asteraceae	H	EW, TU,GD,SU,AR,HA
**2**	*Crotalaria rosenii*	Fabaceae	S	SU, AR, BA, KF, SD
**3**	*Cyperus distans*	Cyperaceae	H	AF, GD,SU,IL,KF,GG,SD
**4**	*Euphorbia ampliphylla*	Euphorbiaceae	T	TU, GD, GJ, WU, SU, IL, KF SD,HA
**5**	*Impatiens tinctoria*	Balsaminaceae	H	GD, SU, AR KF, SD, BA

**6**	*Kalanchoe petitiana*	Crassulaceae	H	EW, GD,WU,SU,AR,BA,HA
**7**	*Maytenus adat*	Celastraceae	T	SU, AR, SD GG
**8**	*Olea capensis subsp. Macrocarpa*	Oleaceae	T	GD, SU, AR, IL, KF, SD, BA
**9**	*Pycnostachys abyssinica*	Lamiaceae	S	IL ,KF, GG, SD
**10**	*Solanecio gigas*	Asteraceae	S	GD, GJ, WU, SU, AR, SD, IL, KF, BA, HA
**11**	*Solanum incanum*	Solanaceae	S	EW, EE, TU, AF, SU, GG, SD, BA, AR
**12**	*Tagetus minuta*	Asteraceae	H	EW, TU, GD, WU, SU, KF, SD, BA, HA
**13**	*Trifolium semiplosum*	Fabaceae	H	EW, TU, GD, WU, SU, AR, BA, SD

where Ha=habit, H=herb. S=shrub, T=tree

Identification of plant community types

In this study, hierarchical agglomerative cluster analysis using R.4.2.2 using the cluster and Vegan packages was used to classify vegetation into plant community types. The ratio and Ward's method were used to draw a dendrogram showing the association between five clusters based on percent species coverage data (Fig. 4). Therefore, the data matrix for this analysis contained 72 quadrates and 234 species clustered using agglomerative hierarchical clustering analysis. Plant community names were assigned based on one or two characteristic species showing a high synoptic mean abundance value and a significant indicator value (Table 4) according to [37]. In this study, a species was considered an indicator of a group if its indicator value was significant ($P < 0.05$) (Table 3). The descriptions of the five community types identified in Arjo-Diga Forest are given below.

262 Table 3. Value of indicator species among identified communities and their significant P value

Indicator species	Community types	Indicator value	P value
Ehretia cymosa	1	0.603	0.001***
Erythrococca trichogyne	1	0.254	0.022*
Euphorbia ampliphylla	1	0.364	0.003*
Pruns africana	1	0.353	0.002*
Tecela noblis	1	0.202	0.05*
Bersama abyssinica	2	0.284	0.033*
Cyathula cylindrica	2	0.217	0.026*
Flacourtia indica	2	0.282	0.04*
Trichilia dregeana	2	0.551	0.004*
Acaccia abyssinica	3	0.874	0.001***
Buddleja polystachya	3	0.24	0.022*
Girardinia diversifolia	3	0.218	0.025*
Grewia bicolar	3	0.366	0.003*
Lippia adoensis	3	0.208	0.029*
Millettia ferruginea	3	0.22	0.055*
Vernonia auriculifera	3	0.279	0.032*
Combertum collinum	4	0.276	0.044*
Gnidia glauca	4	0.229	0.021*
Maytenus obscura	4	0.568	0.003*
Guizotia villosa	4	0.164	0.049*

Combretum molle	5	0.262	0.01*
Ficuse exasperata	5	0.376	0.003*
Ficus vasta	5	0.276	0.015*
Flueggea virosa	5	0.425	0.002*
Gardenia ternifolia	5	0.563	0.001***
Grewia ferruginea	5	0.203	0.055*
Piliostigma thonningii	5	0.704	0.001***
Stereospermum kunthianum	5	0.341	0.005**
Syzygium guineense.subsp.afromontanum	5	0.446	0.001***
Terminalia macroptera	5	0.599	0.001***

Significance codes * = ($p < 0.5$), ** = ($p < 0.01$), *** = ($p < 0.001$)

Maesa lanceolata-Ehretia cymosa community type

This community type is located at the highest elevation of the study areas (1608-1992 m.a.s.l.) and included 8 quadrats and 110 species. The most dominant tree species were *Albizia gummifera, Celtis africana, Millettia ferruginea* and *Prunus africana*. The dominant species in the herbaceous layer include *Agratum conzoides, Bidens pilosa Achyranthus aspra, Digitaria abyssinica* and *Piper capense*. The common lianas/climbers of this community were *Clematis simensis, Dioscorea praehensilis, Phytolaca decandra, Rhamnus prinoides, Landolphia buchananii, Cyphostemma cyphopetalum* and *Urera hypselodendron*. This community contained five indicator species with significant indicator values ($P < 0.05$) (Table 3). This community contained the economically most important species, such as *Coffea arabica, Dioscorea*

praehensilis and *Piper capense. Pteridium aquilinum* is the only dominant fern species found in this community.

Trichilia dregeana – Ficus vasta community type

This community was found between 1537 and 1631 meters above sea level and included 23 quadrats and 148 species. *Syzygium guineense ssp. guineense, Ficus vasta* and *Croton macrostachyus* were the dominant tree species along with *Trichilia dregeana* and *Flacourtia indica.* Other associated tree species are *Apodytes dimidiata, Combertum collinum, Ficus mucuso, Warburgia ugandensis* and *Cordia africana*. It formed three indicator species, namely, *Cyathula cylindrical, Flacourtia indica* and *Bersama abyssinica,* with significant indicator values ($P<0.05$) (Table 3). *Maytenus gracilipes* and *Carisa spinarum* were the dominant shrub species. The herb layer was dominated by *Oplismenus hirtellus, Laggera crispata* and *Kalanchoe petitiana*. Lianas in this community include *Jasminum abyssinicum, Saba comonesis, Capparis tomentosa, Smilax anceps, Rubus apetalus* and *Combretum paniculatum.*

Acacia abyssinica-Millettia ferruginea community type

This community is spread over altitudes ranging from 1476 to 1792 m above sea level and includes 8 quadrats and 94 species. *Croton macrostachyus, Albizia gummifera* and *Buddleja polystachya* were the dominant tree species along with *Acacia abyssinica* and *Millettia ferruginea.* Other dominant tree species are *Combertum collinum, Cordia africana, Bridelia micrantha, Croton macrostachyus* and *Maesa lanceolata*. The shrub layer is dominated by *Maytenus gracilipes, Senna petersiana,* Grewia ferruginea, Vernonia auriculifera, Brucea antidysenterica and *Nuxia congesta*. The dominant species of the herb layer were *Sonchus bipontini, Tristemma mauritianum, Achyranthus aspra, Bidens macroptera* and *Oplismenus hirtellus*. The lianas/climbers of this community were *Paullinia pinnata* and *Cyphostemma*

adenocaule. This community formed seven indicator species with significant indicator values (P < 0.05) (Table 3).

Combertum collinum – Bersama abyssinica community type

This community spread at altitudes between 1630-1711 m.a.s.l. *Pouteria adolfi-friederic*i was the only emergent tree species found in this community. *Stereospermum kunthianum, Terminalia macroptera* and *Bridelia micrantha* were dominant tree species next to *Combertum collinum* and *Croton macrostachyus*. Other associated tree species are *Ficus sycomorus, Entada abyssinica, Ximenia americana, Albiza gummifera* and *Flueggea virosa*. The shrub species are dominated by *Clausena anisata, Vepris dainellii, Gardenia ternifolia a*nd *Acanthus polystachius*. Dominant species of the herb layer are *Haumaniastrum villosum, Helinus mystacinus, Indigofera spicata, Senna occidentalis, Pennisetum thumbergi* and *Crotalaria lachnophora*. *Clematis longicauda* and *Clematis simensis* are the most common climbers in this community. This community has three indicator species, namely, *Combertum collinum, Maytenus obscura, Gnidia glauca* and *Guizotia villosa,* with significant indicator values (P < 0.05) (Table 3).

5. *Terminalia macroptera-Piliostigma thonninigii* community type

This community is represented by 10 quadrats and 98 species in an altitude range of 1501 to 1672 meters above sea level. The dominant tree species were *Stereospermum kunthianum, Syzygium guineense subsp. afromontanum, Ficus exasperata, Gardenia ternifolia* and *Flueggea virosa*. Other dominant tree and shrub species are *Combretum molle, Ficus mucuso Albizia malacophylla, Pavetta abyssinica, Maytenus obscura, Ximenia americana, Oxythenteria abyssinica* and *Flacourtia indica*. The herb layer consisted of *Crotalaria ononoides, Aframomum alboviolaceum, Conyza sumatreosis, Alysicarpus rugosus* and *Guizotia villosa.* The most common climbers/vines of this community are *Ampelocissus schimperiana* and *Cissampelos*

pareira. There are also several indicator species with significant indicator values in this group, and the most important are *Gardenia ternifolia*, *Piliostigma thonningii*, *Syzygium guineense subsp. afromontanum* and *Terminalia macroptera* (Table 3).

Table 4. Synoptic mean cover value of plant species in the Arjo Diga forest

Species name	Community types (Cluster)				
	C1	C 2	C3	C4	C 5
Maesa lanceolata	**2.73**	1.87	1.37	0.85	1.8
Ehretia cymosa	**2.32**	0	0	0.1	0
Albizia gummifera	1.73	0.56	1.75	1.5	0
Cordia africana	1.17	1.13	1.33	0.55	1.32
Trichilia dregeana	1.16	**4.33**	1.08	1	3
Flacoutia indica	1.03	**2.18**	2.94	2.6	2.8
Ficus exasperata	0.11	2.9	1.78	2	1.98
Ficus vasta	0.54	**3.18**	0.65	0.25	1.45
Acaccia abyssinica	0.64	1.6	**5.31**	0.1	0
Millettia ferruginea	0.09	1.34	**3.35**	0.15	1
Stereospermum kunthianum	0	1.3	2.33	1.75	3
Apodytes dimidiata	0	1.23	2.24	1.77	0.5
Ficus thonningii	1.09	0	1.67	0.5	0.3
Maytenus gracilipes	0.63	1.34	1.37	0.15	0
Combretum collinum	0.18	2.43	0	**4.54**	0.6
Croton macrostachyus	0.17	1.91	0.34	**2.31**	3.1

Bersama abyssinica	1.63	1.86	0.62	**2.65**	0.9
Combretum molle	0	0.08	0	2.01	1.3
Bridelia macrantha	0.09	1.73	1.67	1.65	0.9
Terminalia macroptera	0	0.56	3.37	1.65	**4.42**
Piliostigma thonningi	0.09	0	2	0.45	**3.34**
Hymenodictylon floribundum	0	0	0.12	0.2	**2.13**
Ximenia americana	0	0.73	1.3	2.1	1
Warburgia ugandensis	0.09	1.17	0.65	1.54	0.4

Richness, Diversity, and Evenness of plant communities

The diversity analysis (i.e., species richness, diversity and evenness) for each plant community type was calculated using the free statistical software R. 4.2.2. The results showed that community 4 had the highest species richness and Shannon diversity index, followed by communities 2, 1 and 5, while community 3 had the lowest species richness and Shannon diversity. On the other hand, community 2, followed by communities 5 and 1, has the highest evenness. Community 4 had the lowest Shannon evenness value (Table 4).

Table 4. Diversity analysis for each of the five plant community types in Arjo-Diga Forest.

Community types	Species richness	Shannon diversity (H')	Shannon- Evenness(J)
Community 1	110	4.1	0.87
Community 2	148	4.3	0.86
Community 3	94	3.9	0.89
Community 4	162	4.4	0.85

Community 5	98	4	0.88

Similarities between plant communities

The plant community similarity results showed that the highest similarity value was calculated between communities 2 and 4, while the lowest community similarity was calculated between communities 3 and 5 (Table 5).

Table 5. Similarities between five types of plant communities in Arjo Diga forest

Community types	CI	C2	C3	C4	C5
C1	1				
C2	**0.57**	1			
C3	0.34	0.36	1		
C4	0.45	**0.79**	0.39	1	
C5	0.35	0.42	0.22	**0.68**	1

C1=*Maesa lanceolata-Ehretia cymosa*, C2=*Trichilia dregeana – Flacourtia indica*, C3=*Acacia abyssinica-Millettia ferruginea*, C4=*Combertum collinum – Croton macrostachyus*, and C5= *Terminalia macroptera- Piliostigma thonninigii*

Impact of environmental variables on plant commuity plant diversity,

As can be seen in Figure 7, different types of communities were affected by elevation, slope, CEC, TN, OC, pH and Av. P. The impact of Phosphorus, OC and Altitude on Community 5 is minimal, despite the presence of outliers. However, it had a significant impact on Community 1. Community 3 was heavily impacted by CEC and TN; Community 5, however, was less affected. Disturbance and pH had a significant impact on community 5 but had a lesser impact on community 3. Slope has a fairly consistent pattern in its effects.

Regression analysis showed that species richness diversity and evenness showed significant differences ($p<0.05$) along environmental gradients (Fig. 5). Regression analysis showed that species richness ($R^2 = 0.07$, $P < 0.024$) (Fig. a) and Shannon diversity ($R^2 = 0.05$, $P < 0.05$) (Fig. b) decreased significantly with increasing altitude. Similarly, TN was negatively associated ($R^2=0.04$, $P<0.05$) with species richness. For example, when TN deposition was greater than 0.2%, species richness decreased **(**Fig. e). On the other hand, slope a showed a significantly negative relationship with Shannon diversity ($R^2 = 0.0122$, $P < 0.009$) (Fig. g) and evenness ($R^2=0.08$, $P< 0.01$) (Fig. H). However, disturbance showed a significant positive relationship with species richness ($R^2=0.11$, $P<0.003$) (Fig. c) and Shannon diversity ($R^2=0.13$, $P<0.001$) (Fig. d).

In the study area, five different types of plant communities were found. As can be seen in Figure 4, different community types were affected by altitude, slope, CEC, TN, OC, pH, and Av. P. The effect of phosphorus, OC, and altitude on Community 5 is minimal, despite the presence of outliers. On Community 1, nevertheless, it had a significant impact. Community 3 was heavily impacted by CEC and TN; however, community 5 was less affected. Disturbance and pH had a significant impact on community 5, but had a smaller one on community 3. Slope has a somewhat consistent pattern in its effects.

Figure 7. The impact of environmental vraiables on plant community

Relationship between community types and environmental factors

CCA ordination

The result of the CCA ordination of 72 quadrates and five environmental variables, presented in Table 6 and Fig. 5, shows the relationship between environmental variables and plant community types and axes. A negative or positive association between the environmental variables and the axis is indicated by the direction of the arrows in the ordination diagram. However, the length of the arrow shows the strength of the association and how it affects them. The results of the Adonis test showed that attitude, total nitrogen and CEC had a statistically significant relationship with community composition and distribution patterns (Table 6). Of all environmental variables, attitude describes 45.24% of the variance in community distributions. The eigenvalues for the first two axes are 0.3504 and 0.2346, respectively. The cumulative percent variance explained by the first two axes accounted for 72.3% of the species-environment variation (Table 7). The distribution of species composition in the plant community was best associated with environmental factors such as altitude (83%), disturbances (62.8%), total nitrogen (32.2%), phosphorus (19.7%) and CEC (13.4%). Table 6. Results of the function Adonis test of environmental variables

Variables	Df	**Sum Of Sqs.**	**R^2**	**F**	**Pr(>F)**
Altitude(m)	1	1.2540	0.04524	3.3734	0.001***
Slope (°)	1	0.4280	0.01544	1.1513	0.270
Disturbance (%)	1	1.2771	0.04608	1.1452	0.172
SOC (%)	1	0.2935	0.01059	0.7861	0.825
TN (%)	1	0.4328	0.01562	1.1643	0.01*
P (ppm)	1	0.2775	0.01039	0.8138	0.79
pH	1	0.3925	0.01416	1.0560	0.409

CEC (cmol kg^{-1})	1	0.5130	0.01851	1.3799	0.05 **
Residuals	63	19.8009	0.84495		
Total	71	27.7161	1.00000		

Significance Level: 0 '***', 0.001 '**', 0.01 '*', 0.05 '

The first CCA axis was significantly negatively correlated with altitude (r=-0.99), TN (r=-0.95), CEC (r=-0.998), and available phosphorus (r=-0.78671) and significantly positively correlated with disorders (r = 0.33740). The second axis was positively associated with CEC and disorders (Table 6). The CCA ordination of the Arjo-Diga forest study plots formed five community types based on species composition. These five community types were separated following the arrows of the environmental variables. The CCA ordination (Fig. 5) showed that community 4 was separated from communities 1, 2, 3, 4 and 5 in the first CCA axis by disturbance. In contrast, in the second CCA axis, communities 1, 2 and 3 are separated from communities 4 and 5 by elevation, CEC, total nitrogen, slope, and soil organic carbon.

Table 6 Monte Carlo permutation test of the relationship between environmental factors and plant community types

Variables	CCA1	CCA2	r2	Pr(>r)
Altitude	-0.99966	-0.02625	0.8302	0.000 ***
Disturbance	0.33740	0.94136	0.6280	0.000***
Total nitrogen	-0.99945	-0.03321	0.3218	0.000***
Phosphorus	-0.78671	-0.61732	0.1973	0.001**
CEC	-0.99983	0.01831	0.1344	0.01 *
Eigenvalue	0.3504	0.2346		

Proportion Explained	0.2710	0.1814		
Cumulative Proportion	0.2710	0.4524		

Significance Level: 0 ‘***’, 0.001 ‘**’, 0.01 ‘*’, 0.05 ‘.’

~~Figure 5. CCA ordination biplot of 72 quadrats and five environmental variables and plant community~~

Table 7. Results of the function Adonis test of environmental variables

Variables	Df	**Sum Of Sqs.**	**R^2**	**F**	**Pr(>F)**
Altitude(m)	1	1.2540	0.04524	3.3734	0.001***
Slope (°)	1	0.4280	0.01544	1.1513	0.270
Disturbance (%)	1	1.2771	0.04608	1.1452	0.172
SOC (%)	1	0.2935	0.01059	0.7861	0.825
TN (%)	1	0.4328	0.01562	1.1643	0.01*
P (ppm)	1	0.2775	0.01039	0.8138	0.79
pH	1	0.3925	0.01416	1.0560	0.409
CEC (cmol kg^{-1})	1	0.5130	0.01851	1.3799	0.05 **
Residuals	63	19.8009	0.84495		
Total	71	27.7161	1.00000		

Significance Level: 0 ‘***’, 0.001 ‘**’, 0.01 ‘*’, 0.05 ‘.

The results of the post hoc mean comparison between environmental variables and plant community types are presented in Table 8. Community type 1 was found at a higher altitude (2011.8±0.3), while community type 5 was found at a lower (15285±0) altitude. The highest disturbance value (2±0.5) was recorded in community type 5, while the lowest disturbance value

(1.4±1.13) was recorded in community 3. The highest CEC value (40.3±6) was recorded in community type 2, while the lowest CEC value (31.2±10.7) was recorded in community type 5. The maximum (4.19) value of soil organic carbon was recorded in community type 2, while the minimum (2.85) value was observed in community type 5. The pH value of the soils varied between them from 4.8±0.5 to 5.1±0.4 (Table 8). Accordingly, community type 4 was found in very strongly acidic soil, while communities 1, 2, 3 and 5 were found in strongly acidic soil.

Table 8. Post hoc mean comparison between environmental variables and plant community types.

Environmental variable	Plant Community Types				
	C1	C2	C3	C4	C5
Altitude(m)	2011.8±0.3^{d}	1761.4±1.2^{a}	1917.4±2.1c	1648.1±.2.5^{b}	15285±0.5^{b}
Slope (%)	21.8±.3.6^{bc}	19.8±11bc	22.7±1.3^{c}	21.1±5a	20.3±1^{a}
Disturbance	1.85±1^{ab}	2±0.6^{ab}	1.4±1.13^{a}	1.57±0.7^{a}	2±0.5ab
pH	5.1±0.4^{a}	5.08±0.7a	5.02±0.5^{a}	4.8±0.5^{b}	5.1±0.3^{a}
CEC	38.8±7.3^{c}	40.3±6^{b}	39.8±8^{b}	32.9^{a}	31.2±10.7^{a}
SOC	3.6^{b}	4.19^{a}	3.19^{b}	4.08^{a}	2.85^{c}

Values in rows and columns with different letters are significantly different ($P < 0.05$).

Correlation between environmental variables, diversity and richness

Slope, disturbance, CEC, TN, and readily accessible phosphorus were all positively and significantly correlated with altitude, according to the Pearson correlation (Table 9). Unexpectedly, the slope and species evenness revealed a negative association. Negative correlations were discovered between the disturbance and SOC, TN, available phosphorus, CEC, species richness, and evenness. Positive correlations between SOC CEC and available

phosphorus were found. The relationship between CEC and available phosphorus was found to be significantly inverse relationships. On the other hand, there was a significant correlation between Shannon diversity and species richness and evenness. The diversity index and all other environmental variables were negatively correlated with soil pH.

Table 9. Pearson correlation between environmental variables and the diversity index.

	**Alt.**	**Slope**	**Dis.**	**SOC**	**TN**	**Avil.P**	**PH**	**CEC**	**H**	**S**	**E**
**Alt.**											
**Slope**	0.18*										
**Dis.**	0.10*	-0.07									
**SOC**	0.19*	-0.23	-0.17*								
**TN**	0.46***	-0.00	-0.26**	-0.17							
**Avil.P**	0.25*	-0.04	-0.28*	0.07*	-0.11						
**PH**	-0.05	-0.13	-0.06	-0.03	-0.08						
**CEC**	0.24***	0.16	-0.27**	-0.16*	-0.00*	0.01*	0.00				
**H'**	0.01*	-0.08	0.12*	0.12	0.08	0.04	-0.1	0.09			
**S**	-0.04*	0.1	-0.08**	-0.12	-0.14	-0.03	0.08	-0.07	0.98***		
**E**	-0.02*	-0.14*	-0.12*	-0.18	0.07	0.01	0.05	-0.03	0.78***	-0.74**	

Note: *** $p < 0.001$, ** $p < 0.01$, * $p < 0.05$ * Alt. = altitude, Dis. = Disturbance, Avil. P= available phosphorus, S = species richness, H′=Shannon diversity, and E= evenness.

Discussion

Species composition of the Arjo-Diga forest

It is not fair to directly compare the species composition and diversity of one forest to another, as there are differences in aspect, elevation, and heterogeneity of habitat[10] and edaphic factors [40]. However, they provide a generalized picture of the species composition and diversity of the

forest in the study area. The results of the present study are compared with results from other moist Afromontane forests in Ethiopia. As a result, the total number of species collected in this study (234 species) was higher than that in other similar studies conducted in the moist Afromontane forest of Ethiopia. Examples include the following forests: Masha forest (130 species; [41]; Jibat forest (183 species [42]; Komto forest, 180 species [43]; Belete forest, 157 species [44]; Gelesha forest, 157 species [45], Gendo forest, 168 species [46], and Agama forest, 162 species [47]. On the other hand, the total number of species in the study area was slightly lower than the number of species recorded in the Bonga forest (243 species) [48]. The number of sample plots and anthropogenic disturbances could explain the differences in species composition between different forest areas [49].

In the study area, seven families contributed 38% of the total number of species in the forest. The families Asteraceae, Fabaceae, Rubiaceae, Euphorbiaceae, Lamiaceae, Acanthaceae, and Poaceae were the most species-rich families in the Arjo-Diga Forest, as reported in the flora of Ethiopia and Eritrea [50]. Similarly, other authors [48] also reported that these families were the most species-rich families in the moist Afromontane Forest of southwestern Ethiopia. The dominance of these families in the study area was due to their adaptation and colonization of different ecological niches due to their efficient mechanisms of pollination, dispersal, and germination. For example, species in the family Asteraceae have an attractive flower color that allows the plant to be easily pollinated by a variety of insects [51]. Herbs account for 36% of the species identified among growth forms. This, associated with the occurrence of canopy gaps caused by anthropogenic disturbance, may result in herbaceous species becoming more

prevalent[52]. Forest disturbance in the form of selective tree felling favors the growth of herbaceous species in forest undergrowth.

The study shows that the Arjo-Diga forest accounts for 20 (8.54%) of the endemic plant species. This number of species is higher than that reported in studies in the Harenna Forest, Berhane-Kontir Forest, Wofwasha Forest, Bonga Forest, Yayu Forest, and Agama Forest[53-55]. This study shows that the large number of endemic species in the study forest is related to various climatic factors prevalent in the area. For example, precipitation and temperature are major contributors to the existence of endemic flora in the area. In addition, the geographic location of the area also leads to the organization of taxa with unique biological characteristics.

Vegetation classification

From the results of the hierarchical cluster analysis, five types of plant communities were identified in the study forest. The plant communities were named after two or more dominant plant species were selected based on their synoptic mean cover values. The dominant plant species were those with the highest mean cover value for a given community. When compared to the other communities, plant communities type I (*Maesa lanceolata-Ehretia cymosa*) and type III (*Acacia abyssinica-Millettia ferruginea*) had the fewest species. This is due to anthropogenic disturbances such as livestock grazing, fire, charcoal production, and firewood collection in the sampled plots. This finding is consistent with the findings of [56], who found that selective cutting of economically significant woody species, as well as livestock grazing, reduces species richness and diversity. This community type also includes economically important tree species such as *Albizia gummifera*, *Millettia ferruginea*, used as coffee shade, *Ficus thonningi*, used as forage, and *Prunus africana*, used as firewood and agricultural implantation. These results

support previous research showing that trees in forests help household members in a variety of ways due to their proximity to forests [57-59]. According to local sources (personal communication with elders), human impact has been observed in the Arjo Diga forest for many years, and most of what is now the forest area was probably formerly farmland and human settlements. This implies that some significant conservation work has been undertaken to reclaim the forest.

Community type II (*Trichilia dregeana- Ficus vasta*) showed higher species richness than the other communities, which is attributed to the suitability of the habitat for plant growth and the presence of rivers and streams in the sampled plots. Similarly, Addi, Soromessa [55] found that the presence of rivers in sample plots and less anthropogenic impact may have contributed to the highest species richness and diversity in plant community composition. On the other hand, community type IV (*Combertum collinum- Bersama abyssinica*), *Apodytes dimidiata*, *Sapium ellipticum*, and *Syzygium guineense ssp. guineense* are distinctive species in the middle canopy of the Arjo-Diga Forest. In addition, this community type included the main species that provide traditional medicine, edible fruits, honey bees, and household materials for the local people. For example, *Schefflera abyssinica* and *Pouteria adolfi-friederici* are dominant emerging tree species for honey bee production, and *Croton macrostachyus, Erythrococca burci, Securridaca longepedunculata*, and *Entada abyssinica* are used for traditional medicine, while Allophylus *abyssinicus, Rhus vulgaris*, *Syzygium guineense* and *Ximenia americana* have been used to provide edible fruits for the local people. A similar investigation was reported in the Yayu forest, Sheko forest, and Bonga forest by [60, 61].

Community type V (*Terminalia macroptera- Piliostigma thonninigii*) consisted of edible tree species such as *Syzygium guineense subsp. afromontanum, Ximenia americana and Gardenia ternifolia* are used as food by many animals and humans. *Stereospermum kunthianum* and *Calpurnia aurea* were used for traditional medicine by the local people in the study area. In addition, this community contained economically important species such as *Aframomum corrorima, Piper capense, and Dioscorea praehensilis,* which are used for traditional medicine and food, respectively. However, they have been influenced by human activities such as logging, coffee plantations, agricultural expansion, and settlement. Therefore, this study suggests that ecosystem conservation ensures the preservation of useful plant species on site by using sustainable harvesting methods to take plants from the forest for any purpose.

Species diversity, richness, evenness and similarity between species of plant communities

In this study, the values of species richness, diversity and evenness in the identified plant communities varied. For example, community four had high species richness and diversity compared to other types of communities. High species diversity and richness in this community could be associated with high solar radiation (conducive to high productivity), high rainfall, and low seasonal variability[62]. However, community 3 had the lowest species richness and diversity due to the proximity of human settlements and high grazing intensity. In addition, it was observed that man-made fires and the expansion of agricultural land were most common where this community was found. Similarly, Gole [63] in the southwestern Afromontane forest and Gurmessa, Soromessa [43] in the Komta moist Afromontane forestalso reported that grazing, agricultural expansion, and human settlement may have significantly reduced species richness and plant community diversity. Species evenness gives the relative abundance of species in quadrates. Low evenness was observed in community four, indicating dominance of some

species, such as *Trichilia dregeana, Pouteria adolfi-friedericii* and *Vepris dainellii*. The possible reason for the low evenness value in this community has been attributed to excessive disturbance and overexploitation of multipurpose species used for timber. However, a high evenness score was found in community 5, suggesting recurring coexistence of species in all plots rather than low dominance of a single species. The evenness values suggest that when the evenness value is high in a given forest, the location of protected areas may not be as important, as opposed to a low forest evenness value.

The similarity/difference between forest communities maintains forest biodiversity and shapes forest composition and structure [64]. Measuring the similarity between forest communities is essential to assess species changes caused by selective tree harvesting on species composition [65]. The reason lies in the different abundances of species and the different intensities of disturbance between them. This study agreed with a study by Fikadu, Melesse [66] and Birhanu, Bekele [67], who reported that environmental factors such as slope, topography, soil and climate have significant effects on vegetation distribution patterns.

. Plant community-environment relationship

The different plant community distribution patterns in the study area reflect the combined effects of altitude, total nitrogen (TN), cation exchange capacity (CEC), and disturbances. Based on the results of CCA, altitude is a significant variable in determining species distribution and community formation in the Arjo-Diga forest. Other studies conducted in Ethiopia found that elevation differences in Ethiopian landscapes affect species composition and plant community distribution patterns in forest vegetation [68-70]. Altitude also plays a crucial role in affecting temperature, humidity, solar radiation, and atmospheric pressure, thereby affecting plant growth and development and vegetation distribution.

Furthermore, our results showed that soil variables such as total nitrogen (TN) and cation exchange capacity (CEC) were significantly associated with plant community formation in the study area. These results suggest that TN and CEC are the most important soil variables in determining floristic composition, diversity, and plant community distribution. For example, soil TN explained significant variation in species in community types 1 and 3 (Fig. 5). These results could be due to the positive effects of total nitrogen on certain highly competitive plants, which competitively exclude weaker species [71]. This result is very similar to a study conducted by Ricart [72], which found a negative trend in species diversity with TN in forests in Pennsylvania. Stevens et al. (2011) also showed that plant community composition changes with TN deposition in European acidic grassland studies. The soil CEC also explained significant variation in the species composition of communities 1 and 2 in the study area. Similar to our results, Dalle, Maas [73] found that CEC affects species distribution in a plant community by varying soil fertility in the soil. Furthermore, Bekele [74] found that CEC had a positive and significant effect on species composition on the central plateau of Shewa, Ethiopia. This study suggested that both the chemical and physical properties of soil significantly affect plant community structure.

In addition, disturbances also significantly explained differences in species composition between plant communities. In this study, we observed that community 5 was the most disturbed plant community due to its proximity to adjacent agricultural activities and human settlements. The species richness of communities 1, 2, and 4 increased at moderately disturbed sites. These results are consistent with the "intermediate disturbance hypothesis"[75], which predicted a peak in species richness at the intermediate level of disturbance. This suggests that species richness is

maximized when ecological disturbances are neither too rare nor too frequent. This finding is consistent with Tamirat and Lulekal [76], who reported that moderately disturbed sites have high species composition and diversity.

Correlation among environmental variables,

Pearson correlation analysis revealed that altitude, TN, SOC, P and CEC were significantly and positively associated (Table 8). The present study shows that altitude is positively correlated with SOC. Anthropogenic disturbances are stronger at lower altitudes (1200-1540 meters above sea level) and smaller at higher altitudes (1700-2200 meters above sea level) and decrease temperature and high precipitation at higher altitudes. These could be some of the many reasons for the increasing trend of SOC along an altitudinal gradient. Our results confirmed those of [Devi and Sherpa [77], 78], who claimed that SOC in the Himalayan forest is positively correlated with altitude. However, Shapkota and Kafle [79] discovered that in Nepal's national park forest soils, the SOC decreased with increasing altitude due to decreased decomposition related to organic carbon production and long-term accumulation. Therefore, this study confirms that altitude is an effective indicator of forest soil organic carbon content.

In addition, the present study showed that slope and SOC were negatively correlated. This is because the slope affects the distribution of SOC by accelerating/slowing SOC degradation by affecting the activity of microorganisms through their influence on soil temperature and water content. In addition, soil erosion is more severe on steeper slopes, which removes organic carbon from the soil, resulting in a reduced SOC. Similar relationships have been reported in northern Ethiopia by [80]. However, in western Ethiopia, Yohannes et al. (2015) reported that there are no correlations between slopes and SOC. In addition, the CEC has shown a positive correlation with

altitude. This is likely due to denser vegetation at higher elevations producing more organic matter[81]. As a result, adding organic matter to the soil can increase its CEC since this organic colloid absorbs cations more effectively than clay colloids. Similar results were reported by [82] in a tea plantation area in Indonesia. In addition, CEC and SOC have a negative relationship. SOM's resistance to decomposition is weakened, leading to a reduction in CEC[83]. Furthermore, we observed positive correlations between SOC and TN, consistent with the results of [84]. Furthermore, TN had a positive correlation with SOC since TN affects SOC accumulation and soil carbon cycles. Additionally, there is a negative correlation between soil pH and soil organic carbon (SOC) because soil pH is important in controlling the diversity of microbial communities that control how quickly soil organic carbon is broken down [85]. Therefore, it is important to learn about soil organic carbon, CEC, total nitrogen and soil pH and the relationship between them and other soil properties.

Conclusion

In this study, the relationship between plant communities and environmental factors (i.e., altitude, slope, soil nutrients and disturbance) was highlighted. We also examined how environmental factors influenced the species richness, diversity and evenness of plant communities in the Arjo-Diga forest in western Ethiopia. The results show that the study area has a high species richness along with endemic species, which requires immediate protection of the forest before species are irrevocably lost. The species richness, diversity and evenness in the individual plant communities varied. The perceived variation in diversity attributes in plant communities could be related to environmental variables such as soil nutrients, disturbance, habitat heterogeneity, and topography. Among the identified plant communities, the *Acacia*

abyssinica-Millettia ferruginea community exhibited the lowest species richness and diversity indices. This calls for immediate conservation action. Among the environmental variables, altitude significantly influenced species diversity and plant community distribution as well as the correlation and regression among other environmental variables. At lower altitudes, the smallest numbers of species were recorded due to high levels of anthropogenic disturbance, such as firewood, selective logging, and extraction of non timber forest products from the forest for income generation over the higher altitudes. As a result the study suggests that the pattern of community formation and vegetation distribution is largely influenced by altitude. In addition, soil nutrients such as soil organic carbon, total nitrogen, available phosphorus, and cation exchange capacity affect the species composition and diversity of the plant community. Furthermore, anthropogenic disturbance, including grazing, selective cutting and fire, also influenced the plant diversity pattern of species distribution. Based on this result, it is recommended to conduct a study on the impact of climate change on plant community formation and species distribution by ecological modeling. In addition, the influence of slope aspect on floristic composition and diversity and the effect of land use change on vegetation cover were suggested, which was not the subject of this study.

Acknowledgements

The authors thank Dilla University for giving the first author the opportunity to continue his doctoral studies. Funding from Dilla University and Addis Ababa University is also recognized. We would also like to thank the Diga District Department of Agriculture for providing the

necessary information for this study. We also thank all the staff of the Ethiopian National Herbarium for their assistance in identifying the flora. We would also like to express our gratitude to the local authorities and community for their kind support and cooperation throughout the data collection

References

1. Dakhil, M.A., et al., *Richness patterns of endemic and threatened conifers in south-west China: topographic-soil fertility explanation.* Environmental Research Letters, 2021. **16**(3): p. 034017.
2. Gebrehiwot, K., et al., *Elevational changes in vascular plants richness, diversity, and distribution pattern in Abune Yosef mountain range, Northern Ethiopia.* Plant diversity, 2019. **41**(4): p. 220-228.
3. Naud, L., et al., *Altitude effects on spatial components of vascular plant diversity in a subarctic mountain tundra.* Ecology and evolution, 2019. **9**(8): p. 4783-4795.
4. Costanza, J.K., et al., *Classifying forest inventory data into species-based forest community types at broad extents: exploring tradeoffs among supervised and unsupervised approaches.* Forest Ecosystems, 2018. **5**(1).
5. Nepali, B.R., J. Skartveit, and C.B. Baniya, *Impacts of slope aspects on altitudinal species richness and species composition of Narapani-Masina landscape, Arghakhanchi, West Nepal.* Journal of Asia-Pacific Biodiversity, 2021. **14**(3): p. 415-424.
6. Liu, Y., et al., *Effects of contemporary environment and Quaternary climate change on drylands plant diversity differ between growth forms.* Ecography, 2019. **42**(2): p. 334-345.
7. Pandey, K., Y. Adhikari, and M. Weber, *Structure, composition and diversity of forest along the altitudinal gradient in the Himalayas, Nepal.* Appl Ecol Environ Res, 2016. **14**(2): p. 235-251.
8. Gairola, S., et al., *Species richness and diversity along an altitudinal gradient in moist temperate forest of Garhwal Himalaya.* Am J Sci, 2009. **5**: p. 119-128.

9. Trigas, P., et al. *Elevational gradient of vascular plant species richness and endemism in Crete*. in *Poster 248 at the Fifth Biennial Conference of the International Biogeography Society, Irakleion, Crete, Greece*. 2011.

10. Ahmad, M., et al., *Patterns of plant communities along vertical gradient in Dhauladhar Mountains in Lesser Himalayas in North-Western India.* Science of The Total Environment, 2020. **716**: p. 136919.

11. Bennie, J., et al., *Influence of slope and aspect on long-term vegetation change in British chalk grasslands.* Journal of ecology, 2006. **94**(2): p. 355-368.

12. Fadrique, B. and J. Homeier, *Elevation and topography influence community structure, biomass and host tree interactions of lianas in tropical montane forests of southern Ecuador.* Journal of vegetation science, 2016. **27**(5): p. 958-968.

13. Waring, B.G., J.M. Becknell, and J.S. Powers, *Nitrogen, phosphorus, and cation use efficiency in stands of regenerating tropical dry forest.* Oecologia, 2015. **178**(3): p. 887-897.

14. Fisher, J.B., et al., *Nutrient limitation in rainforests and cloud forests along a 3,000-m elevation gradient in the Peruvian Andes.* Oecologia, 2013. **172**(3): p. 889-902.

15. Stevens, C.J., et al., *Impact of nitrogen deposition on the species richness of grasslands.* Science, 2004. **303**(5665): p. 1876-1879.

16. Huston, M., *Soil nutrients and tree species richness in Costa Rican forests.* Journal of Biogeography, 1980: p. 147-157.

17. Nadeau, M.B. and T.P. Sullivan, *Relationships between plant biodiversity and soil fertility in a mature tropical forest, Costa Rica.* International Journal of Forestry Research, 2015. **2015**.

18. Zhu, Y., et al., *The effects of crop tree thinning intensity on the ability of dominant tree species to sequester carbon in a temperate deciduous mixed forest, northeastern China.* Forest Ecology and Management, 2022. **505**: p. 119893.

19. Shrestha, K.B., et al., *Effect of anthropogenic disturbance on plant species diversity in oak forests in Nepal, Central Himalaya.* International Journal of Biodiversity Science, Ecosystem Services & Management, 2013. **9**(1): p. 21-29.

20. Zhang, J., et al., *Long-term grazing effects on vegetation characteristics and soil properties in a semiarid grassland, northern China.* Environmental Monitoring and Assessment, 2017. **189**: p. 1-13.

21. Willig, M. and S. Presley, *Biodiversity and disturbance.* The encyclopedia of the Anthropocene, 2018. **3**: p. 45-51.

22. Gebretsadik, T., *Causes for biodiversity loss in Ethiopia: A review from conservation perspective.* South Agricultural Research Institute (SARI), Hawassa Agricultural Research center, 2016. **6**(11).

23. Jaleta, G. and H. Jebssa *The impact of large scale agriculture on forest and wildlife in Diga Woreda, Western Ethiopia.* Asian Journal of Agriculture, 2017. **1**(02): p. 100-113.

24. Batjes, N., *Revised soil parameter estimates for the soil types of the world.* Soil Use and Management, 2002. **18**(3): p. 232-235.

25. Mohr, P.A., *The geology of Ethiopia*. 1971: Haile Selassie I University Press.

26. Mueller-Dombois, D. and H. Ellenberg, *Aims and methods of vegetation ecology*. 1974: Wiley.

27. Van der Maarel, E., *Transformation of cover-abundance values in phytosociology and its effects on community similarity.* Vegetatio, 1979. **39**(2): p. 97-114.

28. Nelson, D.a. and L.E. Sommers, *Total carbon, organic carbon, and organic matter.* Methods of soil analysis: Part 2 chemical and microbiological properties, 1983. **9**: p. 539-579.

29. Skjemstad, J. and R. Reeve, *The determination of nitrogen in soils by rapid high-temperature Kjeldahl digestion and autoanalysis.* Communications in Soil Science and Plant Analysis, 1976. **7**(3): p. 229-239.

30. Ryti, R., *On the determination of soil pH.* Agricultural and Food Science, 1965. **37**(1): p. 51-60.

31. Gutierrez Boem, F.H., G. Rubio, and D. Barbero, *Soil phosphorus extracted by Bray 1 and Mehlich 3 soil tests as affected by the soil/solution ratio in Mollisols.* Communications in soil science and plant analysis, 2011. **42**(2): p. 220-230.

32. Chapman, H., *Cation-exchange capacity.* Methods of soil analysis: Part 2 Chemical and microbiological properties, 1965. **9**: p. 891-901.

33. Hadera, G., *A study on the ecology and management of the Dessa forest in the northeastern escarpment of Ethiopia.* Unpublished MSc Thesis Addis Ababa University, 2000.

34. Yeshitela, K. and T. Bekele, *Plant community analysis and ecology of Afromontane and transitional rainforest vegetation of southwestern Ethiopia.* SINET: Ethiopian Journal of Science, 2002. **25**(2): p. 155-175.

35. Woldemichael, L.K., T. Bekele, and S. Nemomissa, *Vegetation composition in Hugumbirda-Gratkhassu national forest priority area, South Tigray.* Momona Ethiopian Journal of Science, 2010. **2**(2): p. 27-48.

36. Kent, M., *Vegetation description and data analysis: a practical approach*. 2011: John Wiley & Sons.

37. Woldu, Z., *Comprehensive and ecological data analysis: Basics, concepts and methods.* Addis Ababa University, Addis Ababa, 2017. **408**.

38. Hussain, M., et al., *Assessment of Plant communities and identification of indicator species of an ecotonal forest zone at durand line, district Kurram, Pakistan.* Appl. Ecol. Environ. Res, 2019. **17**(3): p. 6375-6396.

39. Sorensen, T.A., *A method of establishing groups of equal amplitude in plant sociology based on similarity of species content and its application to analyses of the vegetation on Danish commons.* Biol. Skar., 1948. **5**: p. 1-34.

40. Hofhansl, F., et al., *Climatic and edaphic controls over tropical forest diversity and vegetation carbon storage.* Scientific Reports, 2020. **10**(1): p. 1-11.

41. Assefa, A., S. Demissew, and Z. Woldu, *Floristic composition, structure and regeneration status of M asha forest, south-west E thiopia.* African Journal of Ecology, 2014. **52**(2): p. 151-162.

42. Burju, T., K. Hundera, and E. Kelbessa, *Floristic composition and structural analysis of Jibat humid afromontane forest, west Shewa zone, Oromia national regional state, Ethiopia.* Ethiopian Journal of Education and Sciences, 2013. **8**(2): p. 11-34.

43. Gurmessa, F., T. Soromessa, and E. Kelbessa, *Floristic Composition and Community Analysis of Komto Afromontane Moist Forest, East Wollega Zone, West Ethiopia.* Science, Technology and Arts Research Journal, 2013. **2**(2): p. 58-69.

44. Gebrehiwot, K. and K. Hundera, *Species composition, plant community structure and natural regeneration status of Belete moist evergreen montane forest, Oromia regional state, Southwestern Ethiopia.* Momona Ethiopian Journal of Science, 2014. **6**(1): p. 97-101.

45. Bilew, A., H. Kitessa, and A. Balcha, *Floristic composition and structural analysis of Gelesha forest, Gambella regional State, Southwest Ethiopia.* Journal of Ecology and the Natural Environment, 2015. **7**(7): p. 218-227.

46. Gemechu, T., E. Kelbessa, and T. Soromessa, *Floristic Composition and Community analysis of Gendo Moist Montane Forest of East Wellega, Western Ethiopia.* Journal of Natural Sciences Research, 2015. **5**: p. 15.

47. Admassu, A., et al., *Floristic composition and plant community types of Agama Forest, an â€ œAfromontane Forestâ€ in Southwest Ethiopia.* Journal of Ecology and the Natural Environment, 2016. **8**(5): p. 55-69.

48. Kelbessa, E. and T. Soromessa, *Interfaces of regeneration, structure, diversity and uses of some plant species in Bonga Forest: A reservoir for wild coffee gene pool.* SINET: Ethiopian Journal of Science, 2008. **31**(2): p. 121-134.

49. Song, X., et al., *Different environmental factors drive tree species diversity along elevation gradients in three climatic zones in Yunnan, southern China.* Plant Diversity, 2021. **43**(6): p. 433-443.

50. Kelbessa, E. and S. Demissew, *Diversity of vascular plant taxa of the flora of Ethiopia and Eritrea.* Ethiopian Journal of Biological Sciences, 2014. **13**(Supp.)): p. 37-45.

51. Gupta, R.K., *Features of Afro-Alpine Plant Ecology.(Acta Phytogeographica Succica 49)*. 1965, JSTOR.

52. Garg, S., R.K. Joshi, and S.C. Garkoti, *Effect of tree canopy on species composition, diversity and biomass of herbaceous vegetation and soil characteristics in semi-arid forests of the Aravalli hills.* 2020.

53. Teketay, D. and T. Bekele, *Floristic composition of Wof-Washa natural forest, Central Ethiopia: Implications for the conservation of biodiversity.* Feddes Repertorium, 1995. **106**(1-5): p. 127-147.

54. Wakjira, F.S., *Biodiversity and ecology of Afromontane rainforests with wild Coffea arabica L. populations in Ethiopia*. 2006: Cuvillier Verlag.

55. Addi, A., T. Soromessa, and T. Bareke, *Floristic composition and vegetation community analysis of Gesha and Sayilem Forest in Kaffa Zone south western Ethiopia.* Biodiversitas Journal of Biological Diversity, 2020. **21**(7).

56. Mulugeta, Y., T. Bekele, and E. Kelbessa, *Floristic Composition, Species Diversity and Vegetation Structure of Gera Moist Montane Forest, Jimma Zone of Oromia National Regional State, Southwest Ethiopia.* Ethiopian Journal of Biological Sciences, 2015. **14**(1): p. 45-68.

57. Muleta, D., et al., *Socioeconomic benefits of shade trees in coffee production systems in Bonga and Yayuhurumu districts, southwestern Ethiopia: farmers' perceptions.* Ethiopian Journal of Education and Sciences, 2011. **7**(1): p. 39-55.

58. Hundera, K., *Shade tree selection and management practices by farmers in traditional coffee production systems in Jimma Zone, Southwest Ethiopia.* Ethiopian Journal of education and sciences, 2016. **11**(2): p. 91-105.

59. Gemechu, H.W., D. Lemessa, and D.B. Jiru, *A comparative analysis of indigenous and exotic tree species management practices in agricultural landscapes of Southwest Ethiopia.* Trees, Forests and People, 2021. **4**: p. 100059.

60. Senbeta, F., et al., *Plant diversity, vegetation structure and relationship between plant communities and environmental variables in the Afromontane Forests of Ethiopia.* SINET: Ethiopian Journal of Science, 2014. **37**(2): p. 113-130.

61. Alebachew, G.W., T.A. Abejew, and Z. Tesema, *Honeybee Colony Management Practices and Identification of Honeybee Floras in Eastern Amhara, Ethiopia.* Journal of Apiculture, 2022. **2**: p. 151-161.

62. Gao, J. and Y. Carmel, *Can the intermediate disturbance hypothesis explain grazing–diversity relations at a global scale?* Oikos, 2020. **129**(4): p. 493-502.

63. Gole, T.W., *Vegetation of the Yayu forest in SW Ethiopia: impacts of human use and implicatiions for in situ conservation of wild Coffea arabica L. Populations*. Vol. 10. 2003: Cuvillier Verlag.

64. Socolar, J.B., et al., *How should beta-diversity inform biodiversity conservation?* Trends in ecology & evolution, 2016. **31**(1): p. 67-80.

65. Hao, M., et al., *Assessing biological dissimilarities between five forest communities.* Forest Ecosystems, 2019. **6**(1): p. 1-8.

66. Fikadu, E., M. Melesse, and A. Wendawek, *Floristic composition, diversity and vegetation structure of woody plant communities in Boda dry evergreen Montane Forest, West Showa, Ethiopia.* International Journal of Biodiversity and Conservation, 2014. **6**(5): p. 382-391.

67. Birhanu, L., et al., *Relationships between topographic factors, soil and plant communities in a dry Afromontane forest patches of Northwestern Ethiopia.* PloS one, 2021. **16**(3): p. e0247966.

68. Kebede, M., et al., *Soil seed bank and seedlings bank composition and diversity of Wondo Genet moist Afromontane forest South Central Ethiopia.* International Journal of Botany, 2012. **8**(4): p. 170-180.

69. Teshome, M., Z. Asfaw, and M. Mohammed, *Diversity Influences Tree Aboveground Carbon Stocks Through Functional Diversity and Functional Dominance in the Dry Evergreen Afromontane Forest of Hararghe Highland, Southeast Ethiopia.* 2020.

70. Dibaba, A., T. Soromessa, and B. Warkineh, *Plant community analysis along environmental gradients in moist afromontane forest of Gerba Dima, South-western Ethiopia.* BMC Ecology and Evolution, 2022. **22**(1): p. 12.

71. Djordjević, V., et al., *Patterns of distribution, abundance and composition of forest terrestrial orchids.* Biodiversity and Conservation, 2020. **29**(14): p. 4111-4134.

72. Ricart, R.D., *Drivers of Plant Diversity and Distribution in a Northern Hardwood Forest: Interacting Effects of Biotic and Abiotic Factors*. 2019: The Ohio State University.

73. Dalle, G., B. Maas, and J. Isselstein, *Plant communities and their species diversity in the semi-arid rangelands of Borana lowlands, southern Oromia, Ethiopia.* Community Ecology, 2005. **6**(2): p. 167-176.

74. Bekele, T., *Phytosociology and ecology of a humid Afromontane forest on the central plateau of Ethiopia.* Journal of Vegetation Science, 1994. **5**(1): p. 87-98.

75. Collins, S.L. and S.M. Glenn, *Intermediate disturbance and its relationship to within-and between-patch dynamics.* New Zealand Journal of Ecology, 1997: p. 103-110.

76. Tamirat, T. and E. Lulekal, *DIVERSITY OF PLANT SPECIES IN GODERE FOREST, SOUTH-WESTERN ETHIOPIA: IMPLICATION FOR IN-SITU CONSERVATION.* Journal of Global Ecology and Environment, 2021: p. 1-13.

77. Devi, S.B. and S.S.S.S. Sherpa, *Soil carbon and nitrogen stocks along the altitudinal gradient of the Darjeeling Himalayas, India.* Environmental monitoring and assessment, 2019. **191**: p. 1-18.

78. Kumar, A., et al., *Forest soil nutrient stocks along altitudinal range of Uttarakhand Himalayas: An aid to Nature Based Climate Solutions.* Catena, 2021. **207**: p. 105667.

79. Shapkota, J. and G. Kafle, *Variation in soil organic carbon under different forest types in Shivapuri Nagarjun National Park, Nepal.* Scientifica, 2021. **2021**.

80. Gebrehiwot, K., et al., *Soil organic carbon stock in Abune Yosef afroalpine and sub-afroalpine vegetation, northern Ethiopia.* Ecological Processes, 2018. **7**(1): p. 1-9.

81. Kilambo, D.L., et al., *Effect of soils properties on the quality of compact Arabica hybrids in Tanzania.* American Journal of Research Communication, 2015. **3**(1): p. 15-19.

82. Zhang, X.-N., et al., *Influence of edaphic factors on plant distribution and diversity in the arid area of Xinjiang, Northwest China.* Arid land research and Management, 2018. **32**(1): p. 38-56.

83. Fang, K., et al., *Decreased soil cation exchange capacity across northern China's grasslands over the last three decades.* Journal of Geophysical Research: Biogeosciences, 2017. **122**(11): p. 3088-3097.

84. Feng, J. and B. Zhu, *A global meta-analysis of soil respiration and its components in response to phosphorus addition.* Soil Biology and Biochemistry, 2019. **135**: p. 38-47.

85. Tripathi, B.M., et al., *Soil pH mediates the balance between stochastic and deterministic assembly of bacteria.* The ISME journal, 2018. **12**(4): p. 1072-1083.

Figure Click here to access/download;Figure;Figure 1 vegetatio.docx

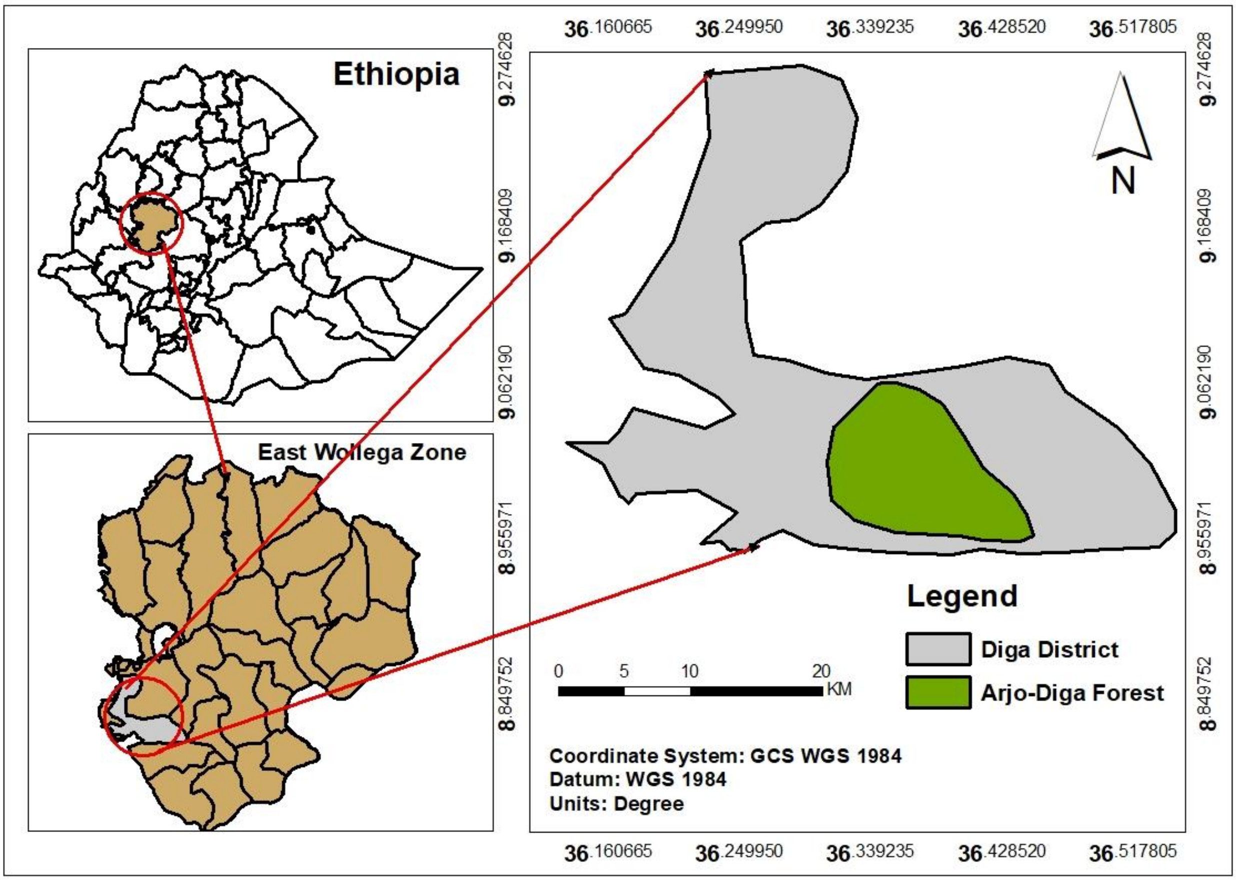

Figure 1. Map of the study area

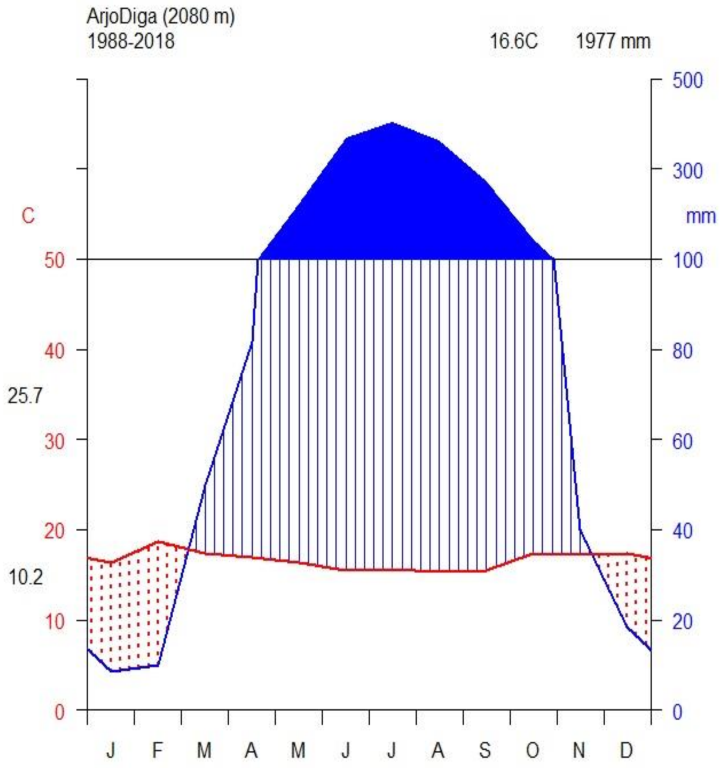

Fig.2. Climate diagram of the study area

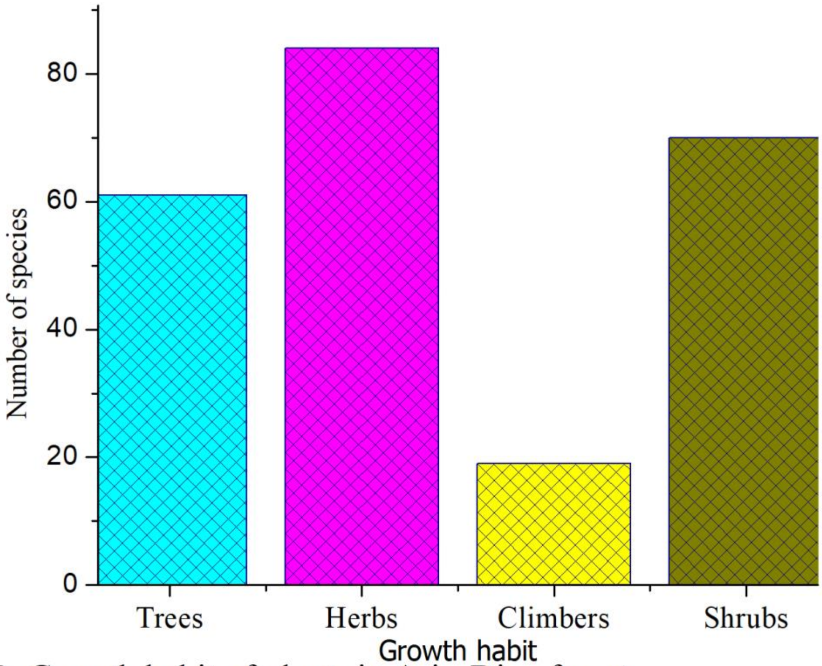

Fig.3. Growth habit of plants in Arjo-Diga forest

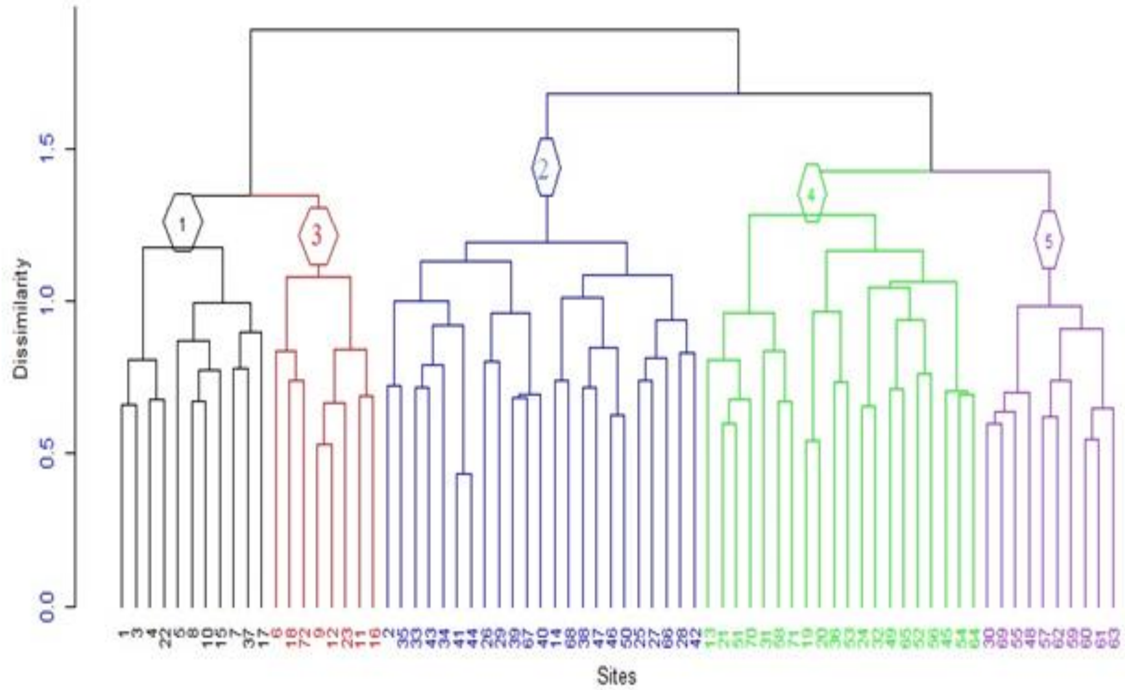

Fig. 4 Dendrogram of agglomerative hierarchical clustering using wards methods showing five community types of the Arjo-Diga Forest

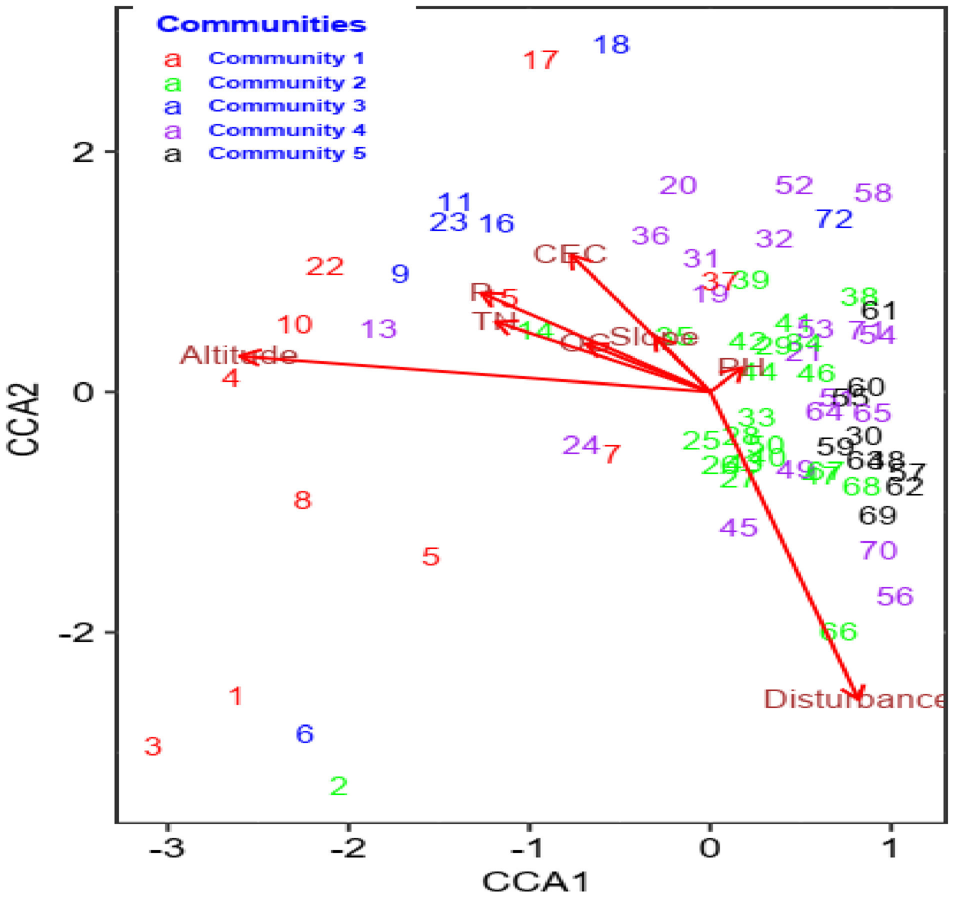

Fig. 5. CCA ordination showing 72 quadrate, five environmental variables and plant community

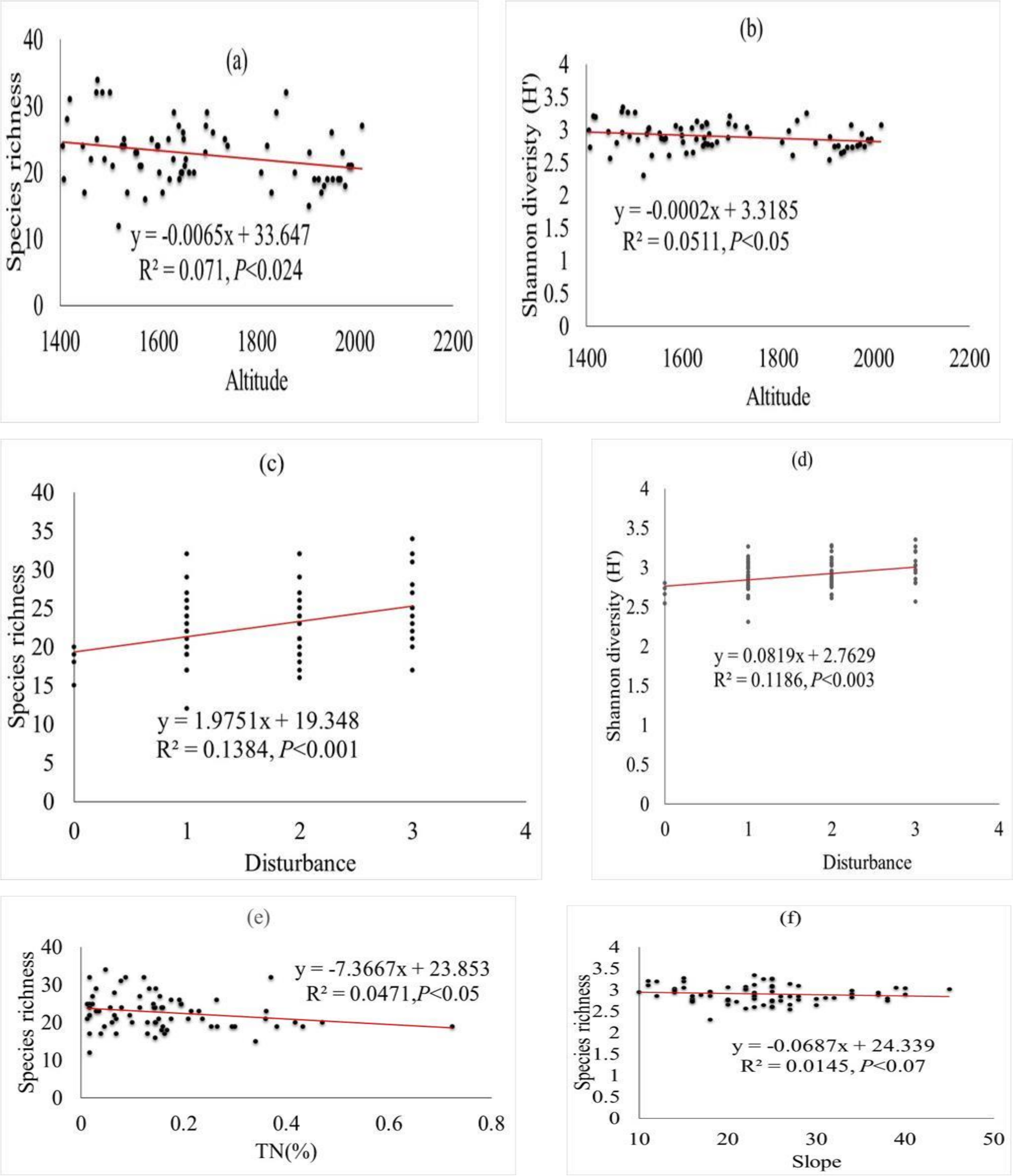
(a)
Species richness
Altitude
y = -0.0065x + 33.647
R² = 0.071, P<0.024
(b)
Shannon diveristy (H')
Altitude
y = -0.0002x + 3.3185
R² = 0.0511, P<0.05
(c)
Species richness
Disturbance
y = 1.9751x + 19.348
R² = 0.1384, P<0.001
(d)
Shannon diversity (H')
Disturbance
y = 0.0819x + 2.7629
R² = 0.1186, P<0.003
(e)
Species richness
TN(%)
y = -7.3667x + 23.853
R² = 0.0471,P<0.05
(f)
Species richness
Slope
y = -0.0687x + 24.339
R² = 0.0145, P<0.07

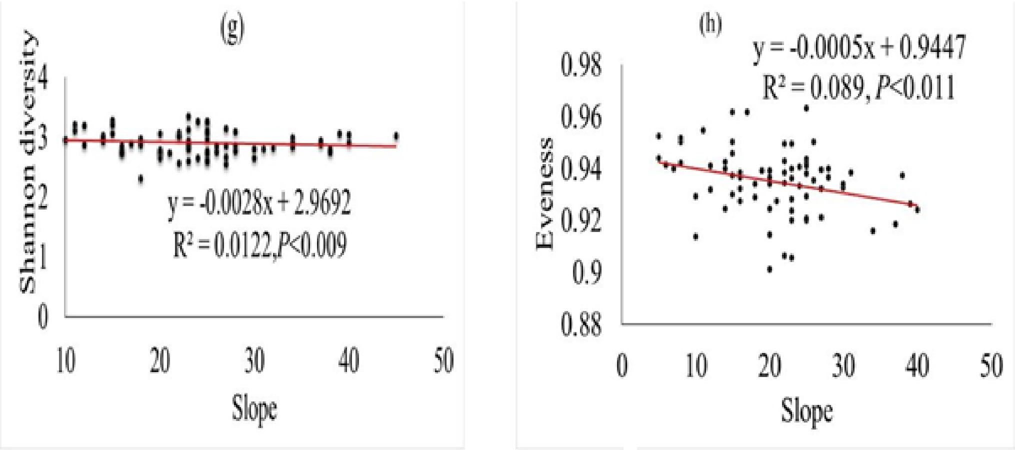

Fig.6.Regression analysis along altitude, slope, TN, & disturbance.Species richness (a,c,e & f), Shannon diversity (b,d & g), and evenness (h).

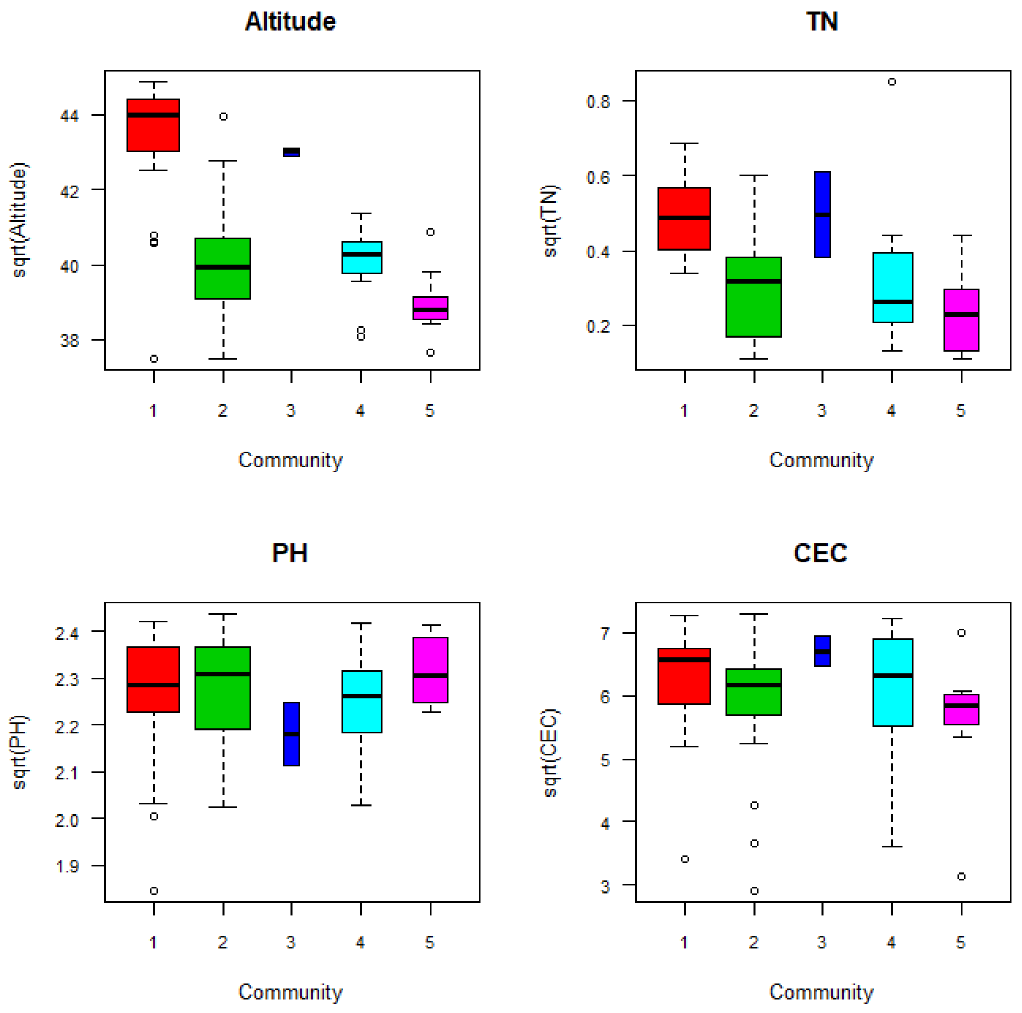
Altitude
sqrt(Altitude)
44
42
40
38
1
2
3
4
5
Community
TN
sqrt(TN)
0.8
0.6
0.4
0.2
1
2
3
4
5
Community
PH
sqrt(PH)
2.4
2.3
2.2
2.1
2.0
1.9
1
2
3
4
5
Community
CEC
sqrt(CEC)
7
6
5
4
3
1
2
3
4
5
Community

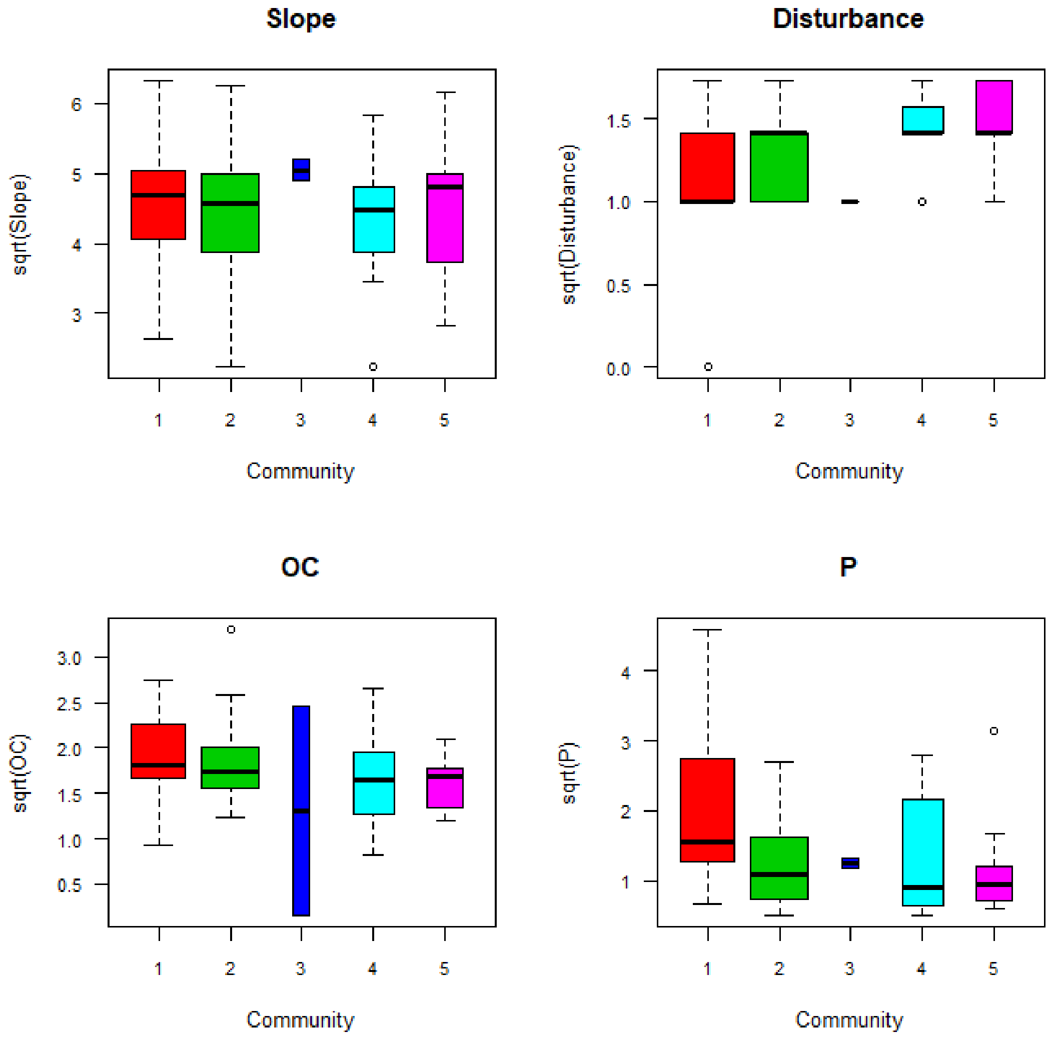

Fig. 7. Impact of environmental variables o plat community types

Click here to access/download
Supporting Information
suuplemetry.docx
