# Supplementary material for: Topography and soil variables drive the plant community distribution pattern and species richness in the Arjo-Diga forest in western Ethiopia
Source: PLoS One. 2024 Aug 6;19(8):e0307888. doi: 10.1371/journal.pone.0307888 (PMC11302921; doi:10.1371/journal.pone.0307888)
Supplement: Attachment — Submitted filename: PONE-D-23-07720_R3_Editor Comments.pdf [file pone.0307888.s009.pdf]

PLOS ONE

# Topography factors and soil variables drive the plant community distribution pattern and species richness in the Arjo Diga forest in western Ethiopia.

--Manuscript Draft--

| | |
|---|---|
| **Manuscript Number:** | PONE-D-23-07720R3 |
| **Article Type:** | Research Article |
| **Full Title:** | Topography factors and soil variables drive the plant community distribution pattern and species richness in the Arjo Diga forest in western Ethiopia. |
| **Short Title:** | Topography and soil drive plant community distribution pattern in the Arjo Diga forest western Ethiopia |
| **Corresponding Author:** | Tariku Berihun<br>Dilla University<br>Addis Ababa, ETHIOPIA |
| **Keywords:** | environmental variables, species diversity, plant communities |
| **Abstract:** | Studies on, species diversity and plant communities along environmental gradients are some of the bases for designing forest management plans. Therefore, this study focused on floristic diversity and the relationship between plant diversity, plant community and environmental variables in the Arjo-Diga forest. For the current study, seventy-two quadrats (30 m x 30 m (900 m2) were laid along altitudinal drops to record vegetation data. All trees and shrubs with a DBH of 2.5 cm and a height of 3 m in each plot were used for data enumeration. Herb and soil data were collected from five subplots of 1 m × 1 m (1 m2) laid at the four corners and center of each quadrat. The diversity indices (i.e. richness, diversity, and evenness) were computed using R.4.2 software. Sorensen's similarity coefficient was used to calculate the similarity between community types. The vegetation classification into community types was calculated using agglomerative hierarchical cluster analysis using R.4.2 software. Relationships between plant community types and environmental variables were analyzed using canonical correspondence analysis (CCA). A total of 234 species belonging to 183 genera and 73 families were collected and identified. The three dominant species-rich families were Asteraceae (29 species), followed by Fabaceae (23 species) and Euphorbiaceae (14 species). Of these, 61 species (26%) were trees, 70 species (30%) were shrubs, 84 species (36%) were herbs, and 19 species (8%) were climbers. Twenty (8.5%) species are endemic to Ethiopia and Eritrea flora. Five plant communities were identified: Maesa lanceolata – Ehretia cymosa, Trichilia dregeana – Flacourtia indica, Acacia abyssinica – Millettia ferruginea, Combertum collinum – Croton macrostachyus and Terminalia macroptera – Piliostigma thonninigii. Community type 4 showed the highest Shannon diversity (4.3) compared to the other communities. A high coefficient of similarity was observed between community types 2 and 4. The CCA ordination showed that the cumulative percentage of variance explained by the first two CCA axes was 72.3% of the variation in the species data. In addition, the CCA ordination diagram showed that plant community type 4 was explained by disturbance in CCA axis 1 and communities 1, 2, 3 and 5 by CEC, TN and altitude in CCA axis 2. Altitude and disturbance are negatively correlated with species richness and evenness. In summary, the Arjo Diga forest contained high species richness, great diversity and endemic species. However, anthropogenic factors such as man-made fires, human settlements, the expansion of farmland, deforestation and cattle grazing have a significant impact on species diversity and plant community formation. However, anthropogenic factors such as human-induced fire, human settlement and farmland expansion, forest clearing, and livestock grazing have significant impacts on species diversity and plant community formation. It is therefore an urgent task to create sustainable plans for the conservation of biodiversity and endemic species in forests. |
| **Order of Authors:** | Tariku Teaw berihun, msc |
| | Zemede Asfaw Woldemariam, Prof. |
| | Tamrat bekele GODE, prof |
| | Ermias Luleka1 Molla, associate prof. |

Powered by Editorial Manager® and ProduXion Manager® from Aries Systems Corporation

| | | |
|---|---|---|
| **Opposed Reviewers:** | Melese bekele wodim, Msc<br>Resercher, Ethiopian Biotechnology Institute: Bio and Emerging Technology Institute<br>melesebekeles@gmail.com<br>he is not an expert in my field of studies |
| | mehar Girmay berhe, Msc<br>Resercher, Ministry of Energy and Water: Ministry of Energy<br>meharigrm@gmail.com<br>he not expert in my area of study |
| | aimiro Likeew Mosee, Msc<br>Resercher, Dilla University<br>aimro2005@gmail.com<br>he has poor background in field of studies |
| **Response to Reviewers:** | We have submitted the revised manuscript as per the comments provided by the editors,and reviewers . Thank you for your guidance and feedback throughout the review process. We appreciate the opportunity to improve our manuscript and address the concerns raised |
| **Additional Information:** | |
| **Question** | **Response** |
| **Financial Disclosure**<br><br>Enter a financial disclosure statement that describes the sources of funding for the work included in this submission. Review the submission guidelines for detailed requirements. View published research articles from *PLOS ONE* for specific examples.<br><br>This statement is required for submission and **will appear in the published article** if the submission is accepted. Please make sure it is accurate.<br><br>**Unfunded studies**<br>Enter: *The author(s) received no specific funding for this work.*<br><br>**Funded studies**<br>Enter a statement with the following details:<br>• Initials of the authors who received each award<br>• Grant numbers awarded to each author<br>• The full name of each funder<br>• URL of each funder website<br>• Did the sponsors or funders play any role in the study design, data collection and analysis, decision to publish, or preparation of the manuscript?<br>• **NO** - Include this sentence at the end of your statement: *The funders had no role in study design, data collection and analysis, decision to publish, or preparation of the manuscript.*<br>• **YES** - Specify the role(s) played. | The author(s) received no specific funding for this work. |

| | |
|---|---|
| * typeset | |
| **Competing Interests**<br><br>Use the instructions below to enter a competing interest statement for this submission. On behalf of all authors, disclose any competing interests that could be perceived to bias this work—acknowledging all financial support and any other relevant financial or non-financial competing interests.<br><br>This statement is required for submission and **will appear in the published article** if the submission is accepted. Please make sure it is accurate and that any funding sources listed in your Funding Information later in the submission form are also declared in your Financial Disclosure statement.<br><br>View published research articles from *PLOS ONE* for specific examples.<br><br>**NO authors have competing interests**<br><br>Enter: *The authors have declared that no competing interests exist.*<br><br>**Authors with competing interests**<br><br>Enter competing interest details beginning with this statement:<br><br>*I have read the journal's policy and the authors of this manuscript have the following competing interests: [insert competing interests here]*<br><br>* typeset | The authors have declared that no competing interests exist. |
| **Ethics Statement**<br><br>Enter an ethics statement for this submission. This statement is required if the study involved: | "N/A" if the submission does not require an ethics statement. |

- Human participants
- Human specimens or tissue
- Vertebrate animals or cephalopods
- Vertebrate embryos or tissues
- Field research

Write "N/A" if the submission does not require an ethics statement.

General guidance is provided below. Consult the submission guidelines for detailed instructions. **Make sure that all information entered here is included in the Methods section of the manuscript.**

Powered by Editorial Manager® and ProduXion Manager® from Aries Systems Corporation

| | |
|---|---|
| **Format for specific study types**<br><br>**Human Subject Research (involving human participants and/or tissue)**<br>• Give the name of the institutional review board or ethics committee that approved the study<br>• Include the approval number and/or a statement indicating approval of this research<br>• Indicate the form of consent obtained (written/oral) or the reason that consent was not obtained (e.g. the data were analyzed anonymously)<br><br>**Animal Research (involving vertebrate animals, embryos or tissues)**<br>• Provide the name of the Institutional Animal Care and Use Committee (IACUC) or other relevant ethics board that reviewed the study protocol, and indicate whether they approved this research or granted a formal waiver of ethical approval<br>• Include an approval number if one was obtained<br>• If the study involved *non-human primates*, add *additional details* about animal welfare and steps taken to ameliorate suffering<br>• If anesthesia, euthanasia, or any kind of animal sacrifice is part of the study, include briefly which substances and/or methods were applied<br><br>**Field Research**<br><br>Include the following details if this study involves the collection of plant, animal, or other materials from a natural setting:<br>• Field permit number<br>• Name of the institution or relevant body that granted permission | |
| **Data Availability**<br><br>Authors are required to make all data underlying the findings described fully available, without restriction, and from the time of publication. PLOS allows rare exceptions to address legal and ethical concerns. See the PLOS Data Policy and FAQ for detailed information. | Yes - all data are fully available without restriction |

| | |
|---|---|
| A Data Availability Statement describing where the data can be found is required at submission. Your answers to this question constitute the Data Availability Statement and **will be published in the article**, if accepted.<br><br>**Important:** Stating 'data available on request from the author' is not sufficient. If your data are only available upon request, select 'No' for the first question and explain your exceptional situation in the text box.<br><br>Do the authors confirm that all data underlying the findings described in their manuscript are fully available without restriction? | |
| **Describe where the data may be found in full sentences. If you are copying our sample text, replace any instances of XXX with the appropriate details.**<br><br>• If the data are **held or will be held in a public repository**, include URLs, accession numbers or DOIs. If this information will only be available after acceptance, indicate this by ticking the box below. For example: *All XXX files are available from the XXX database (accession number(s) XXX, XXX.)*.<br>• If the data are all contained **within the manuscript and/or Supporting Information files**, enter the following: *All relevant data are within the manuscript and its Supporting Information files.*<br>• If neither of these applies but you are able to provide **details of access elsewhere**, with or without limitations, please do so. For example:<br><br>*Data cannot be shared publicly because of [XXX]. Data are available from the XXX Institutional Data Access / Ethics Committee (contact via XXX) for researchers who meet the criteria for access to confidential data.*<br><br>*The data underlying the results presented in the study are available from (include the name of the third party* | All relevant data are within the manuscript and its Supporting Information files. |

| | |
|---|---|
| *and contact information or URL).*<br>• This text is appropriate if the data are owned by a third party and authors do not have permission to share the data.<br><br>* typeset | |
| Additional data availability information: | Tick here if the URLs/accession numbers/DOIs will be available only after acceptance of the manuscript for publication so that we can ensure their inclusion before publication. |

*Powered by Editorial Manager® and ProduXion Manager® from Aries Systems Corporation*

**Cover letters**

Dear editor, Bhoj Kumar Acharya, PhD

Academic Editor of PLOS ONE

I am pleased to submit my manuscript entitled "Topography Factors and Soil Variables Drives Plant Community Distribution Pattern and Species Richness in Arjo-Diga Forest in Western Ethiopia" to the journal PLOS ONE for publication. As researchers and ecologists, we believe this paper is consistent with the aims and scope of the journal and contributes to the growing body of knowledge on the role of environmental variables in shaping plant community distribution and diversity metrics such as diversity, richness, and uniformity in vegetation ecology

The study examined the influence of various environmental variables, including elevation, slope, TN, SOC, CEC, soil pH, available phosphorus and disturbances, on plant community structure and distribution. The results of the study indicated that several of these factors had a significant impact on the plant community and species composition.

Specifically, the study found that altitude, CEC, TN, and disturbances are key factors affecting plant community structure and distribution. The altitude of the study area plays a crucial role in shaping the plant community, probably due to its influence on temperature and precipitation patterns. CEC, a measure of the soil's ability to exchange positively charged ions with the soil solution, was also found to be an important factor influencing tree species composition in plant community.

In addition, the study found that TN, an important nutrient for plant growth, has a significant impact on plant community structure and distribution. The availability of TN in the soil can affect which plant species can thrive in a given area. Finally, the study concluded that disturbances such as human activities such selective cutting, fuel wood collection and agricultural expansion had a significant impacts on the plant community and its diversity, likely through their impact on soil properties and nutrient availability. In addition, soil nutrients such as PH, TN, and CEC also show a significant positive association with altitude. However, altitude and disturbance are negatively correlated with species richness and abundance.

I have thoroughly read and understood the PLOS ONE journal submission guidelines and ensured that my manuscript meets all required requirements. I am confident that our study will meet the journal's strict standards and will make an important contribution to the scientific community. We also answered all questions and comments from the editor and two reviewers sent on May 18, 2023. We have responded the editors' and two reviewers' comments attached through journal submission guidelines as: Editor's Response, Reviewer 1 Response, and Reviewer 2 Response

We certify that this paper consists of original, unpublished work which is not under consideration for publication elsewhere. All authors have approved the manuscript and agree to its submission to PLOS ONE. Finally we assured that we don’t receive any fund for doing this research article.

Thank you for considering my manuscript, and I look forward to hearing from you soon.

Sincerely,

Thank you for your consideration!

Sincerely,

Tariku Berihun
Corresponding author

Manuscript

Click here to access/download;Manuscript;Revized manuscript without tracked changes.doc

# *Topography factors and soil variables drive the plant community distribution pattern and species richness in Arjo-Diga Forest in Western Ethiopia.*

Tariku Berihun*¶[1], Tamrat Bekele¶[2], Ermias Lulekal¶[2], and Zemede Asfaw¶[2]

[1]Department of Biology, Dilla University, Dilla, Ethiopia

[2]Department of Plant Biology and Biodiversity Management, Addis Ababa University, Addis Ababa, Ethiopia

*Corresponding Author. E-mail: berihun.tariku@yahoo.com

¶These authors contributed equally to this work

**Abstract**

This study aimed to examine the influence of topography and soil variables on plant community distribution patterns and species richness in the Arjo-Diga Forest. A total of 72 plots, each measuring 900 m$^2$, were established along altitudinal gradients to collect vegetation data. Agglomerative cluster analysis and Canonical Correspondence Analysis (CCA) using R software were employed to identify plant communities and analyze the relationship between plant community types and environmental variables. In this study, Asteraceae, Fabaceae, and Euphorbiaceae were the dominant families, represented by 17 genera and 29 species (12.4%), 16 genera and 23 species (9.8%), and 10 genera and 14 species (6%), respectively. The agglomerative hierarchical cluster analysis identified five plant communities: *Maesa lanceolata-Ehretia cymosa, Trichilia dregeana-Flacourtia indica, Acacia abyssinica-Millettia ferruginea, Combretum collinum-Croton macrostachyus*, and *Terminalia macroptera-Piliostigma thonninigii.* The CCA results demonstrated significant relationships between species distribution variations and environmental factors, including altitude, disturbance, total nitrogen, and cation exchange capacity (CEC). The findings suggest that species richness and diversity in each plant

community (communities 1, 2, 3, 4, and 5) are directly influenced by altitude, cation exchange capacity, total nitrogen, slope, and disturbances, with altitude being the most influential factor. The study also revealed the significant impacts of anthropogenic factors such as human settlement, expansion of agricultural land, and livestock grazing on plant communities and species distribution patterns. Therefore, the study highlights the urgent need for sustainable practices to protect species diversity and endemic species in forest ecosystems. In conclusion, this study provides valuable insights into the floristic diversity and plant community types in the Arjo-Diga forest. It emphasizes the importance of sustainable forest management plans that consider the impact of human activities on species diversity and the formation of plant communities.

Keywords: Environmental variables, anthropogenic factors, species diversity, plant communities

# Introduction

Investigating the relationship between vegetation attributes, such as richness, diversity, and evenness, and environmental factors is crucial for designing effective conservation activities [1]. The interactions among various environmental factors can lead to changes in habitat conditions, which in turn directly or indirectly affect species distribution and plant diversity worldwide. The composition, diversity, and spatial distribution patterns of plant species are influenced by both abiotic and biotic factors [2]. Understanding the complex relationship between plants and abiotic factors is essential, and further research into their influence on species distribution and the formation of plant communities is urgently needed [3].

Most studies of environmental factors have focused on elevation, topography, and soil to study how environmental factors affect species composition and plant diversity in different ecosystems. Altitude, in particular, drastically alters abiotic factors such as water, temperature, soil composition, and area, which in turn affect plant growth and development [4, 5]. Altitude also regulates the response of plant communities to environmental factors on global scale [6]. A study by Pandey et al. [7] found different patterns of species richness along different elevation gradients. Several studies have shown that species richness patterns decrease monotonically from lowest to highest elevation [8, 9] or monotonically increase. Hump-shaped patterns have also been reported at mid-elevations [2, 10]. However, some researchers have shown that species richness is low at mid-elevations [11]. These contrasting results suggest that species richness along elevation gradients is not a general trend since many species exhibit different phenotypic traits, such as different leaf characteristics, biomass, and phenology. As a result, patterns of species richness and distribution of plant species along altitudinal gradients often differ in ways that are important to conservationists.

Soil nutrients play a crucial role in shaping vegetation structure and plant diversity by creating heterogeneous habitats. Deficiencies in soil nutrients have been reported to impact various aspects of forest ecosystems in tropical forests, including community structure, plant biomass, tree height, and basal area [12]. Conversely, forests with high species richness often exhibit high nutrient dynamics in certain locations. The influence of soil factors, such as total nitrogen (TN) and available phosphorus, on plant community structure appears to be well-established [13]. Total nitrogen (TN), in particular, acts as a limiting factor for plant growth [14] and significantly influences plant diversity and community composition. Additionally, studies have demonstrated

that species richness can be influenced by high nitrogen deposition [15]. In tropical forests, soil cation exchange capacity (CEC) has been found to impact tree species richness [16, 17]. However, there are still knowledge gaps that need to be addressed in order to fully understand how soil cation exchange capacity (CEC) affects tree species richness in tropical forests.

Species composition, diversity, and distribution are also influenced by anthropogenic disturbances such as agricultural expansion, settlement, overgrazing of livestock, selective cutting, and fires [18]. Disturbance has a positive effect on vegetation properties to a certain extent [19]. However, disturbances usually have a negative effect on vegetation since the relationship between vegetation features and disturbances is often negative [20, 21]. Under conditions of severe disturbance, species richness and diversity are low because most species cannot tolerate frequent destructive events. However, due to dominant competitors and fast colonizers, high species richness can be predicted at moderate levels of disturbance [22]. It is important to understand how species respond to human-caused disturbances to make informed conservation decisions. This understanding enables effective actions such as habitat conservation and restoration necessary to maintain critical ecological phenomena such as species distribution limits.[23].

Various authors have documented the results of studies on biodiversity, structure, and regeneration status in different moist Afromontane forests in different parts of Ethiopia. However, many of their species are threatened, endangered, or locally extinct due to habitat destruction, fragmentation, and overexploitation of forest products and habitats [24]. Understanding the interaction between species diversity and composition and the relationship

between environmental factors and plant community types remains critical to the development of forest management strategies.

The Arjo-Diga forest is a complex ecosystem influenced by a variety of local and regional environmental factors. Factors such as soil nutrients, disturbance and microclimate are critical at the local scale and have significant impacts on the distribution pattern of species in the region. Soil properties and disturbances can affect species distribution and composition, while human activities such as logging and grazing have negative impacts on species diversity and composition. Similar results have been reported in other parts of southwestern Ethiopia [25, 26] and demonstrate the key role of soil nutrients such as total nitrogen, cation exchange capacity and soil organic carbon in floristic variation. In addition, natural disturbances, such as fires, can create opportunities for new species to emerge [27, 28]. It is important to understand that these factors are important for forest management to promote healthy and diverse plant communities and mitigate the negative impacts of human activities.

The Arjo-Diga forest, facing degradation from both human and man-made factors, was designated as a biodiversity conservation area in 2014. Despite its protected status, the forest continues to be exploited by the surrounding population for activities such as agricultural expansion, timber extraction, and charcoal production. Unfortunately, no studies have been conducted to assess species composition or analyze plant communities in these areas. Therefore, the present study aims to fill this knowledge gap by conducting a plant community analysis across environmental gradients and investigating the influence of environmental factors on plant diversity, including richness, diversity, and evenness.

Specifically, the objectives of this study were (1) to examine the floristic composition and diversity of the Arjo-Diga forest, (2) to assess the impact of environmental variables on plant diversity characteristics (such as richness, diversity, and evenness), and (3) to explore the relationship between plant community types and environmental variables.

# Materials and methods

## Study area

The Arjo-Diga forests are situated in southwestern Ethiopia and encompass a total area of 12,683.6 hectares. The elevation in the region ranges from 1,200 to 2,220 meters above sea level (m.a.s.l.). Specifically, the forests are located in Diga District, within the East Wollega Zone of the Oromia Regional State. They are positioned 12 km away from Nekemte town and approximately 340 km from Addis Ababa, the capital of Ethiopia. Geographically, the forests are situated between 9°59′00″ and 9°6′30″ north latitude and 36°18′30″ and 36°24′30″ east longitude (Fig 1). The forests are bounded by three districts: Guto Gida, Chewaka, Sasiga, and Leka Dulecha.

The topography of the area exhibits variability, ranging from flat and gentle slopes to moderate and steep slopes. Slope gradients range from 7% to 30% in the southwest and from 30% to 60% in the northeast, making the region more susceptible to severe erosion. According to [29], the study area comprises two agroecological zones: the lowlands (51.4%) and the midlands (48.6%). The midlands are characterized by steep slopes and are predominantly covered by forests.

**Fig 1. Map of Africa showing, Ethiopia, the Oromia region and the study district (Source: OCHA, 2021(https://data.humdata.org/dataset/cod-ab-eth))**

## Soil and Geology

Acriosols are the dominant soil types in the study area These soils are heavily weathered and acidic, with a low cation exchange capacity [30]. They are characterized by a low pH value and insufficient phosphorus content. The dominant soil colors observed were red in the midlands and black in the lowlands. Geologically, the study area comprises Precambrian rocks of high origin, including migmatites [31].

## Climate

The study area mainly experiences tepid to cool subhumid mid-highlands in the northeastern part and hot to warm humid lowlands in the northwestern part. The maximum temperature was 25.7 °C, and the minimum temperature was 0.2 °C (Fig 2). The mean annual precipitation is approximately 1977 mm/year with a unimodal precipitation pattern. The monthly mean precipitation trend shows its maximum in July, June and August and is dry from November and extends into January.

**Fig 2. Climate diagram of study area**

### Vegetation types

The vegetation type of the Arjo-Diga forest is part of the humid–Afromontane forest and is characterized by broadleaf evergreen species[32].This vegetation is found at an altitude between 1500 and 2342 m.a.s.l. with characteristic emergent species, including *Pouteria adolf-friederici*. Other distinctive species in this forest are *Sapium ellipticum*, *Celtis africana*, *Euphorbia ampliphylla*, *Ficus sur*, *Schefflera abyssinica*, *Syzygium guineense ssp. afromontanum*, *Nuxia congesta*, *Galiniera saxifraga*, *Rytigynia Neglecta*, *Vepris dainellii*, *Rothmannia urcelliformi* and several *Albizia* species [32]. *Combretum paniculatum*, *Landolphia buchananii* and *Urera*

*hypselodendron* were the dominant woody climbers in the forest. The western part of the study area borders the Didessa valley, which forms the *Combretum-Terminalia* vegetation.

# Methods of data collection

## Vegetation data collection

A reconnaissance survey was conducted from October 2010 to January 2013 to select sampling sites and to decide on the possible number of transect lines to be laid down. After a reconnaissance survey, nine transect lines were systematically laid along an altitudinal gradient from 1360 to 2220 m.a.s.l. from the crest of the base. A total of 72 sampling plots, each measuring 900 m2 (30 m x 30 m), were sampled to record woody species Vegetation data were collected from a total of 72 plots along nine transects that were systematically constructed using the sampling approach described by [33]. Each plot was relocated every 100 meters along the transect lines. Of the 72 plots, a single plot measuring 30 m x 30 m (900 $m^2$) was created to record tree species. Within each of the main plots, five 5 m x 5 m (25 $m^2$) subplots were created to record shrubs species. Within each 25 $m^2$ subplot, five 2 m x 2 m (4 $m^2$) subplots were created, one in the center and four at the corner of the plot, to record herbaceous species. Finally, the percentage cover of each species in each plot was visually estimated and later converted to a 1–9 modified Braun-Blanquet scale [34]. Voucher specimens in each plot were collected, coded, pressed, and dried. Using the Flora of Ethiopia and Eritrea (volumes 1–8), all voucher specimens was identified and have been deposited in the National Herbarium of Ethiopia, Addis Ababa University.

## Environmental Data

Altitude, latitude and longitude for each plot were recorded using Garmin GPS H72. The slope of sample plot was measured using Suunto clinometer. Soil samples were taken from these 72 sample plot for soil analysis .The soil samples were taken a depth of 0 to 30 cm. Soil samples were taken from each plot at five locations, one in the center and four in the corner, and these mixed to obtain a composite sample and then one sample was taken for analysis. Samples were air dried, ground, and passed through a 2 mm sieve to remove rock fragments and large root particles prior to analysis. Soil samples were analyzed at the Bedele's soil laboratory for selected soil chemistry including soil pH, organic carbon, available phosphorus, total nitrogen and cation exchange capacity Soil organic carbon was determined using the dichromate oxidation method [35] and total soil nitrogen was analyzed using the Kjeldahl digestion and distillation method [36]. Soil pH was determined using a pH meter at a soil- to- liquid ratio of 1:2.5 [37], available phosphorus was extracted using the Bray-1 method [38] and using a spectrophotometer and CEC was determined using the following ammonium acetate method [39].

In each main plot, we examined various forms of human disturbance, including firewood collection, the presence of beehives, charcoal production, timber extraction, footpath creation, and expansion of agricultural land through deforestation, the presence of coffee cultivation, tree debarking, and grazing. To assess the level of human intervention, we recorded indicators such as the presence or absence of stumps, logs, grazing intensity, and signs of firewood collection. We estimated the intensity of forest grazing by visually observing its impact on livestock, including manure droppings and grass clippings. Additionally, we examined the distance between each plot and the forest edge, as well as the distance to nearby houses. To categorize the different types of disturbances, we qualitatively ranked them based on the remaining evidence

observed in each plot. We assigned ratings of relatively absent (indicating no disorder), low, medium, or high, with corresponding values of 0, 1, 2, and 3, respectively, for each level of disturbance[40, 41]. By summing the results for each plot, we created an overall ranking of human disturbance in each community. Higher ranks indicate higher levels of human disturbance, while lower ranks indicate lower levels of disturbance.

### Ordination

For this study, we employed Detrended Correspondence Analysis (DCA) as the chosen ordination method to analyze vegetation data. The selection of the best ordination methods was based on the criteria that if the longest axis length of the DCA exceeds 4, it indicates heterogeneity [42]. In this study, the longest axis length observed was 4.2 SD, confirming the presence of heterogeneous data. Consequently, we decided to utilize a unimodal technique and opted for canonical correspondence analysis (CCA) to illustrate the relationships between plant communities, plots, and environmental variables The study considered a total of 72 plots, 234 species, and 8 environmental variables, which included elevation, slope, pH, available phosphorus, total nitrogen (TN), cation exchange capacity (CEC), disturbances, and soil organic carbon (SOC).

## Data analysis

### Plant community classification

Cluster analysis is a useful tool for grouping observations, such as plots, based on their floristic similarities, specifically the number of plant species present in each plot [43]. In this study, a data matrix consisting of 72 plots and 234 plant species was employed for hierarchical cluster analysis, which allows for the grouping of plant species based on their similarities. For the

cluster analysis in this study, an agglomerative hierarchical approach was utilized, employing Ward's method and a similarity ratio. This method helps classify vegetation into distinct plant community types based on species abundance. We utilized the free statistical software R version 4.2.22 [44] along with the Cluster and Dendextend packages [45] to perform the cluster analysis.

An indicator species analysis (ISA) was conducted using the indicator value (Indval) approach to identify diagnostic plant species for the classification of plant community types. To perform this analysis, several packages were utilized, including Vegan, Cluster, and Labdsv [46].The indicator values of each species were evaluated based on the probability of indicator values across specific iterations. In this analysis, plant species that exhibited a statistically significant indicator value at $p<0.05$ were considered indicator species for a particular plant community type. Ultimately, two of the most dominant plant species within each cluster were selected to assign names to the community types.

## Species diversity

The Shannon diversity index and Shannon evenness were calculated to assess the species diversity, richness, and evenness of the study area and plant communities[47].

$$H' = -\sum_{i=1}^{s} \text{Pi} \ln \text{Pi} \qquad (1)$$

where H'= Shannon diversity index; S=number of species, pi=proportion of ith species. ln= the natural logarithm.

The Shannon evenness index (J) was calculated by using:

$$J = \frac{H'}{H'\text{max}} \qquad (2)$$

where $H'$= Shannon–Wiener diversity index and Hmax = lns, where s is the number of species in the plot.

Sorensen's similarity coefficient (Ss) was used to compare the similarity between two communities [48, 49].

$$Ss = \frac{2a}{(2a+b+c)} \quad (3)$$

where 'a' represents the number of species that are present in both communities, 'b' represents the number of species unique to the first community, and 'c' represents the number of species unique to the second community[50].

## Ordination

Ordination is a multivariate analysis method in ecology that uses an ordination plot to study the relationships among species, plots, plant communities, and environmental variables in a two-dimensional space [42]. Pearson's correlation coefficient was used to calculate correlations between environmental variables using SPSS version 20 software [51]. For this analysis, canonical correspondence analysis (CCA) was used to test relationships between species, plant communities, and environmental variables. In addition, multiple linear regression models were used to test the relationship between environmental variables and diversity attributes at $p<0.05$. Two-way ANOVA followed by a post hoc Tukey HSD test was used to determine if there were significant mean differences between plant communities and environmental variables

# Results

## Floristic composition

A total of 234 species, belonging to 183 genera and 73 families, were documented in this study. The five families with the highest species richness were Asteraceae (29 species), Fabaceae (23 species), Euphorbiaceae (14 species), Lamiaceae (12 species), and Rubiaceae (11 species). In contrast, eight families each had between four and six species (Table 1).The recorded species were classified into different growth forms, including trees, shrubs, herbs, and climbers. Among them, trees accounted for 61 species (26%), shrubs comprised 70 species (30%), herbs constituted 84 species (36%), and climbers represented 19 species (8%) (Fig 3).

Out of the total identified plant species, 20 species (8.5%) were endemic to the flora of Ethiopia and Eritrea (S1 Appendix). Among these species, *Crotalaria rosenii, Impatiens tinctoria, Kalanchoe petitiana, Pycnostachys abyssinica*, and *Solanecio gigas* were some of the newly recorded endemic species in the Wollega floristic region (Table 2). Furthermore, there were approximately 13 species (5.5%) from 10 families and 13 genera that were newly recorded in the Wollega floristic region (Table 2).

**Table 1.Families with more than three genera and their representative number of species**

| Family (N=73) | No of genera (No=183) | No of species (N=234) |
|---|---|---|
| Asteraceae | 17(10%) | 29(12.4%) |
| Fabaceae | 16(8.7%) | 23 (9.8%) |
| Rubiaceae | 11(7%) | 11 (4.7%) |
| Euphorbiaceae | 10(5.5%) | 14 (6%) |

| | | |
|---|---|---|
| Lamiaceae | 10(8.7%) | 12(5.1%) |
| Acanthaceae | 6 (2.9%) | 6(2.6%) |
| Poaceae | 6(2.9%) | 6(2.6%) |
| Malvaceae | 4(2.2%) | 5(2.1%) |
| Rhamnaceae | 4(2.2%) | 5(2.1%) |
| Rutaceae | 4(2.2%) | 4(1.7%) |
| Sapindaceae | 4(2.2%) | 6(2.6%) |
| Verbenaceae | 4(2.2%) | 4(1.7%) |
| Vitaceae | 4(2.2%) | 5(2.1%) |

**Fig 3. Growth habit of plant**

**Table 2. Newly discovered species and endemic species in the Wollega floristic region**

| NO | Species name | Family | Ha. | Floristic Region |
|---|---|---|---|---|
| 1 | *Crepis foetida* | Asteraceae | H | EW, TU,GD,SU,AR,HA |
| 2 | *Crotalaria rosenii** | Fabaceae | S | SU, AR, BA, KF, SD |
| 3 | *Cyperus distans* | Cyperaceae | H | AF, GD,SU,IL,KF,GG,SD |
| 4 | *Euphorbia ampliphylla* | Euphorbiaceae | T | TU, GD, GJ, WU, SU, IL, KF SD,HA |
| 5 | *Impatiens tinctoria** | Balsaminaceae | H | GD, SU, AR KF, SD, BA |
| 6 | *Kalanchoe petitiana** | Crassulaceae | H | EW, GD,WU,SU,AR,BA,HA |
| 7 | *Maytenus arbutifolia (A. Rich.) Wilczek* | Celastraceae | T | SU, AR, SD GG |
| 8 | *Olea capensis subsp.* | Oleaceae | T | GD, SU, AR, IL, KF, SD, BA |

| | | | | |
|---|---|---|---|---|
| | *Macrocarpa* | | | |
| 9 | *Pycnostachys abyssinica** | Lamiaceae | S | IL ,KF, GG, SD |
| 10 | *Solanecio gigas** | Asteraceae | S | GD, GJ, WU, SU, AR, SD, IL, KF, BA, HA |
| 11 | *Solanum incanum* | Solanaceae | S | EW, EE, TU, AF, SU, GG, SD, BA, AR |
| 12 | *Tagetus minuta* | Asteraceae | H | EW, TU, GD, WU, SU, KF, SD, BA, HA |
| 13 | *Trifolium semiplosum* | Fabaceae | H | EW, TU, GD, WU, SU, AR, BA, SD |

where Ha=habit, H=herb. S=shrub, T=tree, and * stands for newly discovered endemic species.

## Plant community types

In this study, hierarchical cluster analysis was employed to identify five distinct plant community types (Fig 4). The dendrogram was constructed using the ratio and Ward's method, illustrating the relationships between the five clusters based on percent species coverage data. The analysis involved clustering a data matrix consisting of 72 plots and 234 plant species using agglomerative hierarchical cluster analysis. Each community type was named after one or two dominant indicator woody species, chosen based on their significant indicator values and synoptic mean abundances (Table 4). In this study, a species was considered an indicator of a particular group if its indicator value was statistically significant ($p < 0.05$) (Table 3). The identified plant communities in this study were *Maesa lanceolata-Ehretia cymosa*, *Trichilia dregeana-Ficus vasta*, *Acacia abyssinica-Millettia ferruginea*, *Combretum collinum-Bersama abyssinica*, and *Terminalia macroptera-Piliostigma thonningii*

**Fig 4. Dendrogram generated from agglomerative hierarchical cluster analysis of vegetation data.1= community type 1, 2= community type 2, 3= community type 3, 4= community type 4, and 5=community type 5.**

**Table 3. Value of the indicator species in identified plant communities and their significant p value.**

Formatted: Font: Bold

| Indicator species | Community types | Indicator value | P value |
|---|---|---|---|
| *Ehretia cymosa* | 1 | 0.603 | 0.001*** |
| *Erythrococca trichogyne* | 1 | 0.254 | 0.022* |
| *Euphorbia ampliphylla* | 1 | 0.364 | 0.003* |
| *Pruns africana* | 1 | 0.353 | 0.002* |
| *Tecela noblis* | 1 | 0.202 | 0.05* |
| *Bersama abyssinica* | 2 | 0.284 | 0.033* |
| *Cyathula cylindrica* | 2 | 0.217 | 0.026* |
| *Flacourtia indica* | 2 | 0.282 | 0.04* |
| *Trichilia dregeana* | 2 | 0.551 | 0.004* |
| *Acaccia abyssinica* | 3 | 0.874 | 0.001*** |
| *Buddleja polystachya* | 3 | 0.24 | 0.022* |
| *Girardinia diversifolia* | 3 | 0.218 | 0.025* |
| *Grewia bicolar* | 3 | 0.366 | 0.003* |
| *Lippia adoensis* | 3 | 0.208 | 0.029* |
| *Millettia ferruginea* | 3 | 0.22 | 0.055* |
| *Vernonia auriculifera* | 3 | 0.279 | 0.032* |
| *Combertum collinum* | 4 | 0.276 | 0.044* |
| *Gnidia glauca* | 4 | 0.229 | 0.021* |
| *Maytenus obscura* | 4 | 0.568 | 0.003* |

| | | | |
|---|---|---|---|
| *Guizotia villosa* | 4 | 0.164 | 0.049* |
| *Combretum molle* | 5 | 0.262 | 0.01* |
| *Ficus exasperata* | 5 | 0.376 | 0.003* |
| *Ficus vasta* | 5 | 0.276 | 0.015* |
| *Flueggea virosa* | 5 | 0.425 | 0.002* |
| *Gardenia ternifolia* | 5 | 0.563 | 0.001*** |
| *Grewia ferruginea* | 5 | 0.203 | 0.055* |
| *Piliostigma thonningii* | 5 | 0.704 | 0.001*** |
| *Stereospermum kunthianum* | 5 | 0.341 | 0.005** |
| *Syzygium guineense.subsp.afromontanum* | 5 | 0.446 | 0.001*** |
| *Terminalia macroptera* | 5 | 0.599 | 0.001*** |

Significance codes * = ($p < 0.5$), ** = ($p < 0.01$), *** = ($p < 0.001$)

1. *Maesa lanceolata-Ehretia cymosa* community type

This particular community type is situated at the highest elevation within the study areas, ranging from 1608 to 1992 m.a.s.l. It encompasses eight plots and comprises a total of 110 species. Among the observed tree species, notable dominants include *Albizia gummifera, Celtis africana, Millettia ferruginea,* and *Prunus africana*. In the herbaceous layer, the dominant species consist of *Agratum conzoides, Bidens pilosa, Achyranthus aspra*, and *Digitaria abyssinica*. Common lianas/climbers found in this community include *Clematis simensis, Dioscorea praehensilis, Phytolaca decandra, Rhamnus prinoides, Landolphia buchananii, Cyphostemma cyphopetalum*, and *Urera hypselodendron*. The community exhibits five indicator species with significant indicator values ($p < 0.05$) (Table 3). Additionally, this community is characterized by the presence of economically important species such as *Coffea arabica, Dioscorea praehensilis*, and

*Piper capense*. *Pteridium aquilinum*, a dominant fern species, exclusively occurs within this community.

2. *Trichilia dregeana – Ficus vasta* community type

This community was found between 1537 and 1631 m.a.s.l and included 23 plots and 148 species. *Syzygium guineense subsp. guineense*, *Ficus vasta* and *Croton macrostachyus* were the dominant tree species along with *Trichilia dregeana* and *Flacourtia indica.* Other associated tree species are *Apodytes dimidiata, Combertum collinum, Ficus mucuso, Warburgia ugandensis* and *Cordia africana*. It formed three indicator species, namely, *Cyathula cylindrical*, *Flacourtia indica* and *Bersama abyssinica,* with significant indicator values ($p<0.05$) (Table 3). *Maytenus gracilipes* and *Carisa spinarum* were the dominant shrub species. The herb layer was dominated by *Oplismenus hirtellus, Laggera crispata* and *Kalanchoe petitiana*. Lianas in this community include *Jasminum abyssinicum, Saba comonesis, Capparis tomentosa, Smilax anceps, Rubus apetalus* and *Combretum paniculatum.*

3. *Acacia abyssinica-Millettia ferruginea* community type

This community is spread over altitudes ranging from 1476 to 1792 m.a.s.l. and includes eight plots and 94 species. The dominant tree species in the community are *Croton macrostachyus, Albizia gummifera, Buddleja polystachya, Acacia abyssinica,* and *Millettia ferruginea*. Other prominent tree species include *Combertum collinum, Cordia africana, Bridelia micrantha, Croton macrostachyus*, and *Maesa lanceolata*. The shrub layer is dominated by species such as *Maytenus gracilipes, Senna petersiana, Grewia ferruginea, Vernonia auriculifera, Brucea antidysenterica,* and *Nuxia congesta*. The herb layer is characterized by dominant species including *Sonchus bipontini, Tristemma mauritianum, Achyranthus aspra, Bidens macroptera*, and *Oplismenus hirtellus*. In terms of lianas and climbers, the community is characterized by

*Paullinia pinnata* and *Cyphostemma adenocaule*. Additionally, this community exhibits seven indicator species with significant indicator values (p < 0.05) (Table 3).

4. *Combertum collinum – Bersama abyssinica* community type

This community spread at altitudes between 1630-1711 m.a.s.l. *Pouteria adolfi-friederic*i was the only emergent tree species found in this community. *Stereospermum kunthianum, Terminalia macroptera* and *Bridelia micrantha* were dominant tree species next to *Combertum collinum* and *Croton macrostachyus*. Other associated tree species are *Ficus sycomorus, Entada abyssinica, Ximenia americana, Albiza gummifera* and *Flueggea virosa*. The shrub species are dominated by *Clausena anisata, Vepris dainellii, Gardenia ternifolia a*nd *Acanthus polystachius*. Dominant species of the herb layer are *Haumaniastrum villosum, Helinus mystacinus, Indigofera spicata, Senna occidentalis, Pennisetum thumbergi* and *Crotalaria lachnophora*. *Clematis longicauda* and *Clematis simensis* are the most common climbers in this community. This community has three indicator species, namely, *Combertum collinum, Maytenus obscura, Gnidia glauca* and *Guizotia villosa*, with significant indicator values (p < 0.05) (Table 3).

5. *Terminalia macroptera-Piliostigma thonninigii* community type

This community is represented by 10 plots and 98 species, situated within an altitude range of 1501 to 1672 m.a.s.l. The dominant tree species in this community include *Stereospermum kunthianum, Syzygium guineense subsp. afromontanum, Ficus exasperata, Gardenia ternifolia,* and *Flueggea virosa.* Other prominent tree and shrub species found in the community are *Combretum molle, Ficus mucuso, Albizia malacophylla, Pavetta abyssinica, Maytenus obscura, Ximenia americana, Oxythenteria abyssinica,* and *Flacourtia indica*. The herb layer is comprised of species such as *Crotalaria ononoides, Aframomum alboviolaceum, Conyza sumatreosis*, *Alysicarpus rugosus*, and *Guizotia villosa*. Among the climbers/vines present in this community,

*Ampelocissus schimperiana and Cissampelos pareira* are the most common. Additionally, several indicator species with significant indicator values have been identified within this community. The most important of these are *Gardenia ternifolia, Piliostigma thonningii, Syzygium guineense subsp. afromontanum*, and *Terminalia macroptera*, as indicated in Table 4.

**Table 4. Synoptic mean cover value of plant species in the Arjo-Diga forest**

| Species name | Community types | | | | |
|---|---|---|---|---|---|
| | C1 | C 2 | C3 | C4 | C 5 |
| *Maesa lanceolata* | 2.73 | 1.87 | 1.37 | 0.85 | 1.8 |
| *Ehretia cymosa* | 2.32 | 0 | 0 | 0.1 | 0 |
| *Albizia gummifera* | 1.73 | 0.56 | 1.75 | 1.5 | 0 |
| *Cordia africana* | 1.17 | 1.13 | 1.33 | 0.55 | 1.32 |
| *Trichilia dregeana* | 1.16 | 4.33 | 1.08 | 1 | 3 |
| *Flacoutia indica* | 1.03 | 2.18 | 2.94 | 2.6 | 2.8 |
| *Ficus exasperata* | 0.11 | 2.9 | 1.78 | 2 | 1.98 |
| *Ficus vasta* | 0.54 | 3.18 | 0.65 | 0.25 | 1.45 |
| *Acaccia abyssinica* | 0.64 | 1.6 | 5.31 | 0.1 | 0 |
| *Millettia ferruginea* | 0.09 | 1.34 | 3.35 | 0.15 | 1 |
| *Stereospermum kunthianum* | 0 | 1.3 | 2.33 | 1.75 | 3 |
| *Apodytes dimidiata* | 0 | 1.23 | 2.24 | 1.77 | 0.5 |
| *Ficus thonningii* | 1.09 | 0 | 1.67 | 0.5 | 0.3 |
| *Maytenus gracilipes* | 0.63 | 1.34 | 1.37 | 0.15 | 0 |
| *Combretum collinum* | 0.18 | 2.43 | 0 | 4.54 | 0.6 |

| | | | | | |
|---|---|---|---|---|---|
| *Croton macrostachyus* | 0.17 | 1.91 | 0.34 | 2.31 | 3.1 |
| *Bersama abyssinica* | 1.63 | 1.86 | 0.62 | 2.65 | 0.9 |
| *Combretum molle* | 0 | 0.08 | 0 | 2.01 | 1.3 |
| *Bridelia macrantha* | 0.09 | 1.73 | 1.67 | 1.65 | 0.9 |
| *Terminalia macroptera* | 0 | 0.56 | 3.37 | 1.65 | 4.42 |
| *Piliostigma thonningi* | 0.09 | 0 | 2 | 0.45 | 3.34 |
| *Hymenodictylon floribundum* | 0 | 0 | 0.12 | 0.2 | 2.13 |
| *Ximenia americana* | 0 | 0.73 | 1.3 | 2.1 | 1 |
| *Warburgia ugandensis* | 0.09 | 1.17 | 0.65 | 1.54 | 0.4 |

## Richness, diversity, and evenness of plant communities

The diversity analysis, including species richness, diversity, and evenness, was conducted for each plant community type. The results revealed that community 4 exhibited the highest species richness and Shannon diversity index, followed by communities 2, 1, and 5. Conversely, community 3 displayed the lowest species richness and Shannon diversity among the analyzed communities. In terms of evenness, community 2 demonstrated the highest evenness, followed by communities 5 and 1. On the other hand, community 4 exhibited the lowest Shannon evenness value, indicating a less balanced distribution of species (Table 5).

**Table 5. Diversity analysis for each of the five plant community types in Arjo-Diga forest.**

| Community types | Species richness | Shannon diversity (H') | Shannon- Evenness (J) |
|---|---|---|---|
| Community 1 | 110 | 4.1 | 0.87 |
| Community 2 | 148 | 4.3 | 0.86 |
| Community 3 | 94 | 3.9 | 0.89 |
| Community 4 | 162 | 4.4 | 0.85 |
| Community 5 | 98 | 4 | 0.88 |

## Similarities between plant communities

According to the results of the plant community similarity analysis, the highest degree of similarity was found between communities 2 and 4. In contrast, the lowest level of similarity was observed between communities 3 and 5 (as indicated in Table 6).

**Table 6.** **Similarities between five types of plant communities in Arjo Diga forest**

| Community types | CI | C2 | C3 | C4 | C5 |
|---|---|---|---|---|---|
| C1 | 1 | | | | |
| C2 | 0.57 | 1 | | | |
| C3 | 0.34 | 0.36 | 1 | | |
| C4 | 0.45 | 0.79 | 0.39 | 1 | |
| C5 | 0.35 | 0.42 | 0.22 | 0.68 | 1 |

C1= *Maesa lanceolata-Ehretia cymosa*, C2=*Trichilia dregeana – Flacourtia indica*, C3=*Acacia abyssinica-Millettia ferruginea*, C4= *Combertum collinum – Crotonc macrostachyus*, and C5= *Terminalia macroptera- Piliostigma thonninigii*

## Relationship between community types and environmental variables

.The results of the CCA ordination, presented in Fig 5, depict the relationship between environmental variables, plant community types, and axes. The direction of the arrows in the ordination diagram indicates a positive or negative association between the environmental variables and the axis. The length of the arrow represents the strength of the association and its impact. According to the results of the Adonis test (Table 7), altitude, total nitrogen, and cation exchange capacity exhibited a statistically significant association with community composition and distribution patterns. Altitude, in particular, explained 45.24% of the variance in community distribution, surpassing other environmental variables. The eigenvalues for the first two axes were 0.35 and 0.23, respectively. The cumulative percentage of variance explained by these two axes accounted for 72.31% of the species-environment variation (Table 8).The distribution of species composition within the plant community was found to be strongly associated with environmental factors such as altitude (83%), disturbances (62.83%), total nitrogen (32.21%), phosphorus (19.72%), and cation exchange capacity (13.43%).

**Fig 5. The CCA ordination diagram displays 72 plots, five environmental variables, and five plant communities**

**Table 7. Results of the Adonis functional test of the environmental variables (significant environmental variables are presented by asterisks with their p values).**

| Environmental variables | Df | Sum of Squares | $R^2$ | F | Pr(>F) |
|---|---|---|---|---|---|
| Altitude (m. a. s. l.) | 1 | 1.2540 | 0.0452 | 3.3734 | 0.001*** |
| Slope (°) | 1 | 0.4280 | 0.0154 | 1.1513 | 0.270 |
| Disturbance (%) | 1 | 1.2771 | 0.0460 | 1.1452 | 0.172 |
| SOC (%) | 1 | 0.2935 | 0.0105 | 0.7861 | 0.825 |
| TN (%) | 1 | 0.4328 | 0.0156 | 1.1643 | 0.01* |
| **Av.**P (ppm) | 1 | 0.2775 | 0.0103 | 0.8138 | 0.79 |
| pH | 1 | 0.3925 | 0.0141 | 1.0560 | 0.409 |
| CEC (cmol $kg^{-1}$) | 1 | 0.5130 | 0.0185 | 1.3799 | 0.05 ** |
| Residuals | 63 | 19.80091 | 0.8449 | | |
| Total | 71 | 27.7161 | 1.0000 | | |

Significance level: 0 '***', 0.001 '**', 0.01 '*', 0.05

The first CCA axis exhibited significant negative correlations with altitude (r = -0.99), TN (r = -0.95), CEC (r = -0.99), and available phosphorus (r = -0.78), while showing a significant positive correlation with disturbances (r = 0.33). On the other hand, the second axis displayed positive associations with CEC and disturbances (Table 8). The CCA ordination of the Arjo-Diga forest study plots revealed the formation of five community types based on species composition. These community types were distinguished by following the arrows of the environmental variables. In the CCA ordination (Fig 5), community 4 was separated from communities 1, 2, 3, and 5 in the first CCA axis by disturbances. Conversely, in the second CCA axis, communities 1, 2, and 3

were separated from communities 4 and 5 by elevation, CEC, total nitrogen, slope, and soil organic carbon.

**Table 8. Monte Carlo permutation test of the relationship between environmental factors and plant community types**

| Environmental variables | CCA1 | CCA2 | $r^2$ | Pr(>r) |
|---|---|---|---|---|
| Altitude | -0.9996 | -0.0262 | 0.8302 | 0.000 *** |
| Disturbance | 0.3374 | 0.9413 | 0.6280 | 0.000*** |
| Total nitrogen | -0.9994 | -0.0332 | 0.3218 | 0.000*** |
| Phosphorus | -0.7867 | -0.6173 | 0.1973 | 0.001** |
| CEC | -0.9998 | 0.0183 | 0.1344 | 0.01 * |
| Eigenvalue | 0.3504 | 0.2346 | | |
| Proportion Explained | 0.2710 | 0.1814 | | |
| Cumulative Proportion | 0.2710 | 0.4524 | | |

Significance Level: 0 '***', 0.001 '**', 0.01 '*', 0.05 '.

The results of the post hoc mean comparison between environmental variables and plant community types are presented in Table 9. Community type 1 was found at a higher altitude (2011.8±0.3), while community type 5 was found at a lower (15285±0) altitude. The highest disturbance value (2±0.5) was recorded in community type 5, while the lowest disturbance value (1.4±1.13) was recorded in community 3. The highest CEC value (40.3±6) was recorded in community type 2, while the lowest CEC value (31.2±10.7) was recorded in community type 5. The maximum (4.19) value of soil organic carbon was recorded in community type 2, while the minimum (2.85) value was observed in community type 5. The pH value of the soils varied

between them from 4.8±0.5 to 5.1±0.4 (Table 9). Accordingly, community type 4 was found in very strongly acidic soil, while communities 1, 2, 3 and 5 were found in strongly acidic soil.

**Table 9. Mean comparison between environmental variables, and plant community types.**

| Environmental variable | Plant Community Types | | | | |
|---|---|---|---|---|---|
| | C1 | C2 | C3 | C4 | C5 |
| Altitude(m) | 2011.8±0.3[d] | 1761.4±1.2[a] | 1917.4±2.1c | 1648.1±.2.5[b] | 15285±0.5[b] |
| Slope (%) | 21.8±.3.6[bc] | 19.8±11bc | 22.7±1.3[c] | 21.1±5a | 20.3±1[a] |
| Disturbance | 1.85±1[ab] | 2±0.6[ab] | 1.4±1.13[a] | 1.57±0.7[a] | 2±0.5ab |
| pH | 5.1±0.4[a] | 5.08±0.7a | 5.02±0.5[a] | 4.8±0.5[b] | 5.1±0.3[a] |
| CEC | 38.8±7.3[c] | 40.3±6[b] | 39.8±8[b] | 32.9[a] | 31.2±10.7[a] |
| SOC | 3.6[b] | 4.19[a] | 3.19[b] | 4.08[a] | 2.85[c] |

Values in rows and columns with different letters are significantly different ($p < 0.05$).

## Influence of environmental variables on plant community and plant diversity

Regression analysis revealed significant differences ($p<0.05$) in species richness, diversity, and evenness along environmental gradients (Fig 6). Specifically, the analysis showed that species richness ($R^2 = 0.07$, $p< 0.05$) (Fig 6A) and Shannon diversity ($R^2 = 0.05$, $p < 0.05$) (Fig 6B) decreased significantly with increasing altitude. Furthermore, species richness exhibited a negative association with TN ($R^2=0.04$, $p<0.05$), indicating that higher TN deposition led to a decrease in species richness (Fig 6E). Conversely, the slope demonstrated a significant negative association with Shannon diversity ($R^2 = 0.0122$, $p < 0.01$) (Fig 6G) and evenness ($R^2=0.08$, $p< 0.01$) (Fig 6H), suggesting that steeper slopes were associated with lower diversity and evenness.

Interestingly, disturbance showed a significant positive association with species richness ($R^2$=0.11, p<0.003) (Fig 6C) and Shannon diversity ($R^2$=0.13, p<0.001) (Fig 6D), indicating that areas with higher disturbance levels exhibited higher species richness and diversity.

**Fig 6. Illustrates the regression analysis conducted to examine the relationships between altitude, slope, TN, and disturbance with species richness (Figs 6A, 6C, 6E, and 6F), Shannon diversity (Figs 6B, 6D, and 6G), and evenness (Fig 6H).**

As depicted in Fig 7, the different types of plant communities were influenced by several environmental factors, including altitude (Fig 7A, slope (Fig 7E, CEC (Fig 7D), TN), OC (Fig 7G), pH (Fig 7C), and available phosphorus (Fig 7H). The impact of available phosphorus, OC, and altitude on Community 5 was minimal, although some outliers were observed. However, these factors had a significant influence on Community 1. Community 3 showed a strong association with CEC and TN, while Community 5 exhibited a lesser sensitivity to these variables. Disturbance and pH had a notable effect on Community 5, but their impact was relatively lower on Community 3. Slope demonstrated a fairly consistent pattern in its effects across all communities (Fig 7E).

**Fig 7. Illustrates the influence of environmental variables on different plant community types: Community type 1: *Maesa lanceolata-Ehretia cymosa*, Community type 2: *Trichilia dregeana-Ficus vasta*, Community type 3: *Acacia abyssinica-Millettia ferruginea*, Community type 4: *Combertum collinum-Bersama abyssinica*, and Community type 5: *Terminalia macroptera-Piliostigma thonninigii***

## Pearson correlation between environmental variables, diversity, richness, and evenness

According to the Pearson correlation analysis (Table 9), several variables exhibited significant positive correlations with altitude, including slope, disturbance, CEC, TN, and Av. P. Unexpectedly, slope and species evenness showed a negative association. Negative correlations

were found between disturbance and SOC, TN, Av. P, CEC, species richness, and evenness. This suggests that higher disturbance levels were associated with lower values of these variables. Positive correlations were found between SOC, CEC and Av.P, indicating that higher levels of these variables were associated with each other. Additionally, a significant inverse relationship was observed between CEC and Av. P. On the other hand, there was a significant association between Shannon diversity and species richness and evenness. The diversity index and all other environmental variables were negatively correlated with soil pH in indicating that higher pH levels were associated with lower diversity and environmental values.

Table 10. Pearson correlation coefficients between environmental factors, diversity index in Diga district, western Ethiopia

| | Alt. | Slope | Dis. | SOC | TN | Av.P | pH | CEC | H' | S | E |
|---|---|---|---|---|---|---|---|---|---|---|---|
| Alt. | | | | | | | | | | | |
| Slope | 0.18* | | | | | | | | | | |
| Dis. | 0.10* | -0.07 | | | | | | | | | |
| SOC | 0.19* | -0.23 | -0.17* | | | | | | | | |
| TN | 0.46*** | -0.00 | -0.26** | -0.17 | | | | | | | |
| Av.P | 0.25* | -0.04 | -0.28* | 0.07* | -0.11 | | | | | | |
| pH | -0.05 | -0.13 | -0.06 | -0.03 | -0.08 | | | | | | |
| CEC | 0.24*** | 0.16 | -0.27** | -0.16* | -0.00* | 0.01* | 0.00 | | | | |
| H' | 0.01* | -0.08 | 0.12* | 0.12 | 0.08 | 0.04 | -0.1 | 0.09 | | | |
| S | -0.04* | 0.1 | -0.08** | -0.12 | -0.14 | -0.03 | 0.08 | -0.07 | 0.98*** | | |
| E | -0.02* | -0.14* | -0.12* | -0.18 | 0.07 | 0.01 | 0.05 | -0.03 | 0.78*** | -0.74** | |

*. Correlation is significance at the 0.05 level (2-tailed),

**. Correlation is significant at the 0.01 level (2-tailed),

Alt. = altitude, Dis. = Disturbance, Av.P= available phosphorus, SOC= Soil organic carbon, TN= Total nitrogen, CEC= Cation exchange capacity H′= Shannon diversity, S = species richness, and E= evenness.

# Discussion

## Species richness of the Arjo-Diga forest

The Arjo-Diga Forest is rich in species richness consisting of economically important species such as *Coffea arabica* and important timber trees namely *Cordia africana*, *Pouteria adolfi friederici*, and *Prunus africana* However, direct comparisons of species richness between forests may not be meaningful due to differences in sample sizes and study objectives [52]. But the Arjo-Diga Forest consisted of 234 species which is higher than Masha forest (130 species [53]; Jibat forest, 183 species [54]; Komto forest, 180 species [55]; Belete forest, 157 species [56]; Gelesha forest, 157 species [57], Gendo forest, 168 species [58], Agama forest, 162 species [59] and Gerba Dima forest,180 species [26]. This could be attributed to its extensive topographic gradient. On the other hand, the total number of species is slightly less than the number of species recorded in the Bonga forest (243 species) [60]. This could be attributed to the number of sample plots, geographical location and disturbance history, as well as climatic factors, which could illustrate the differences in species richness between different forest areas [61].

Asteraceae, Fabaceae, Rubiaceae, Euphorbiaceae, Lamiaceae, Acanthaceae, and Poaceae contributed 38% of the total number of species in the study forest. These dominant families have also been reported as the seven most species-rich families in the flora of Ethiopia and Eritrea [62] and other moist Afromontane forests of southwestern Ethiopia[26, 55, 60]. The dominance of these families in the study area is consistent with general dominance in the flora area and

tropical forests. This is due to their adaptation and colonization of different ecological niches based on their efficient pollination, dispersal, and germination mechanisms. For example, species from Asteraceae have an attractive flower color that allows the plant to be easily pollinated by a variety of insects [63].

Among the identified growth forms, herbs account for 36% of the species. The spread of herb could be attributed to the presence of canopy gaps  caused by anthropogenic disturbance, which may leads to an increase in the prevalence of herbaceous species t[64]. Additionally, forest disturbances in the form of selective tree clear-cutting promote the growth of herbaceous species in the forest understory. Similarly, study conducted in moist Afromontane forests of Ethiopia by[26, 55, 65] also reported a significant presence of herbs, alongside other growth forms such as trees and shrubs.

Arjo-Diga forest is the home to 20 (8.54%) of the endemic plant species, which is .a higher numbered compared to the endemic species found in Harenna Forest, Berhane-Kontir Forest, Wofwasha Forest, Bonga Forest, Yayu Forest, and Agama Forest[27, 66, 67]. This This can be attributed to extreme micro-climate condition, including moisture  precipitation, temperature and spatial heterogeneity, which contribute to the presence of endemic species [68]. Additionally, the diverse topography of the study forest, characterized by hills, valleys, ridges, and plateaus, plays a crucial role in supporting the existence of endemic species.

## Plant community

The hierarchical cluster analysis conducted on the Arjo-Diga forest revealed the presence of five distinct plant communities. These communities were identified by selecting two or more dominant plant species based on their synoptic mean cover values. The dominant plant species were determined by their highest mean cover value within each community. Compared to the

other communities, plant communities type 1 (*Maesa lanceolata-Ehretia cymosa*) and type 3 (*Acacia abyssinica-Millettia ferruginea*) exhibited the lowest species diversity. This is can be attributed to anthropogenic disturbances such as cattle grazing, fire, charcoal production, and firewood collection in the plots studied. This finding is consistent with the findings of [69], who reported that selective logging of economically important woody species and livestock grazing can decrease species richness and diversity. These community type also include economically important tree species such as *Albizia gummifera* and *Millettia ferruginea*, used as coffee shade; *Ficus thonningi*, used as forage; and *Prunus africana,* used as firewood and agricultural tools. These results support previous research indicating that trees in forests provide various benefits to household due to their proximity [70-72]. According to local sources (personal communication with elders), human impacts on the Arjo-Diga forest have been observed for many years, and a significant effort have been made to restore and conserve the forest.

The type 2 community (*Trichilia dregeana- Ficus vasta*) showed higher species richness than the other communities, which is attributed to the suitability of the habitat for plant growth and the presence of rivers and streams in the studied plots. Similarly,[67] found that the presence of rivers in sample plots and lower anthropogenic influence may have contributed to the highest species richness and diversity in plant community composition. On the other hand, community type 4 (*Combertum collinum- Bersama abyssinica*), *Apodytes dimidiata*, *Sapium ellipticum,* and *Syzygium guineense ssp. guineense* are distinctive species in the middle canopy of the Arjo-Diga forest. In addition, this community type included the main species that provided traditional medicine, edible fruits, honey bees, and household materials to the local population. For example, *Schefflera abyssinica* and *Pouteria adolfi-friederici* are dominant emerging tree species for honey bee production, and *Croton macrostachyus*, *Erythrococca burci*, *Securridaca*

*longepedunculata*, and *Entada abyssinica* are used for traditional medicine, while *Allophylus abyssinicus*, *Rhus vulgaris*, *Syzygium guineense* and *Ximenia americana* are used to provide local people with edible fruit. A similar investigation was reported in Yayu Forest, Sheko Forest, and Bonga Forest by [73, 74].

Community type 5 (*Terminalia macroptera- Piliostigma thonninigii*) consisted of edible tree species such as *Syzygium guineense subsp. afromontanum*, *Ximenia americana* and *Gardenia ternifolia* are used as food by many animals and humans. *Stereospermum kunthianum* and *Calpurnia aurea* were used for traditional medicine by the local people in the study area. In addition, economically important species such as *Aframomum corrorima*, *Piper capense*, and *Dioscorea praehensilis* were present in this community and are used for traditional medicine and food. However, they have been influenced by human activities such as coffee plantations, agricultural expansion, and human settlement. Therefore, this study suggests that protecting the ecosystem ensures the conservation of beneficial plant species on site by using sustainable harvesting methods to take plants from the forest for any purpose.

## Species diversity, richness, and evenness in identified plant communities.

The results of this study indicate slight differences in species richness, diversity, and evenness among the five plant communities. Community types 2 and 4 exhibit relatively high species richness and diversity, while community type 3 has the lowest values. These differences may be attributed to factors such as altitude, human impacts, grazing, and other biotic and abiotic variables among the five community types. Community types 2 and 4 have the highest species diversity and richness because they are situated at moderate altitudes, which create favorable conditions for rapid resource acquisition and growth of most plants [26, 75]. On the other hand, community type 3, located in close proximity to human settlements primarily between 1476 to

1792 m.a.s.l, experiences lower species richness and diversity. Similar studies conducted elsewhere have also reported reduced plant diversity and richness in forests near settlements [76]. Additionally, altitude and climatic factors such as temperature, precipitation, humidity, and seasonal fluctuations play a role in determining species richness and diversity [55, 77]. Studies conducted in the southwestern Afromontane forest [26] and the Komta moist Afromontane forest [55] have also observed that anthropogenic disturbances including grazing, agricultural land expansion, and human settlement, can diminish species richness and diversity. Community type 4 demonstrates low plant evenness due to the presence of disturbances and overexploitation of multipurpose species used for timber and other purposes. This leads to the dominance of certain species such as *Trichilia dregeana, Pouteria adolfi-friedericii,* and *Vepris dainellii* within community type 4. Conversely, the highest plant evenness is observed in community type 5, indicating recurring coexistence of species across all plots rather than the low dominance of a single species. Hence, the significance of evenness values suggests that in forests, high evenness in specific locations within protected areas may not be as critical as in forests with low evenness.

The similarity/difference between forest communities maintains forest biodiversity and shapes forest composition and structure [78]. Measuring similarity between forest communities is important to assess species changes attributed to species composition through selective tree harvesting [79]. The reason lies in the different abundances of species and the different intensities of disturbance between them. This study was consistent with a study by [80], who reported that environmental factors such as slope, topography, soil and climate have significant effects on vegetation distribution patterns.

## Relationship between environmental variables and plant community

In a given study area, the distribution patterns of plant communities are often influenced by a combination of factors, including altitude, slope, soil nutrients, and disturbances. Specifically, in the study area of the Arjo-Diga forest, the distribution of plant communities is influenced by altitude, total nitrogen (TN), cation exchange capacity (CEC), and disturbances. The results of Canonical Correspondence Analysis (CCA) indicate that altitude is a significant variable in determining the distribution and formation of plant communities in the Arjo-Diga forest. Similar findings have been reported in other studies conducted in Ethiopia, highlighting the impact of elevation variations on the distribution patterns of forest vegetation [26, 81, 82]. By focusing on these factors, conservation efforts can effectively contribute to the preservation and restoration of plant communities in the study area. This, in turn, promotes biodiversity and ensures the overall health of the ecosystem.

Furthermore, our results showed that soil variables such as total nitrogen (TN) and cation exchange capacity (CEC) were significantly associated with plant community formation in the study area. These results suggest that TN and CEC are the most important soil variables in determining plant community distribution. For example, the soil TN explained the significant variation in species composition in community types 1 and 3 (Fig 5). These results could be due to the positive effects of TN on certain strongly competing plants, which forecloses species in competition [83]. This result is very similar to a study by [84], who found a negative trend in species diversity in the plant community with TN in forests in Pennsylvania. Similarly, a study by (2011) showed that plant community composition can be influenced by total nitrogen (TN) deposition in European acidic grassland ecosystems. The research found that changes in TN deposition resulted in significant changes in plant community composition. Soil cation exchange

capacity (CEC) was also found to be a major factor in explaining the differences in species composition between communities 1 and 2 in the Arjo-Diga forest. Similar to our results [85], CEC affects tree species in a plant community by varying soil fertility in the soil. In addition,[86] found that CEC had a positive and significant impact on the species composition of the plant community on the central plateau of Shewa, Ethiopia. The results of this study suggest that the structure of plant communities is significantly influenced by soil chemical and physical properties.

In addition, disturbances also significantly explained differences in species composition between plant communities. In this study, we found that community 5 was the most disturbed plant community due to its proximity to adjacent agricultural activities and human settlements. The study showed that the species richness of communities 1, 2, and 4 increased in the studied plot with a moderate level of disturbance. These results are consistent with the "intermediate disturbance hypothesis" [87], which suggests that species richness is maximized when ecological disturbances occur at intermediate frequency. This finding is consistent with [88], who reported that plant communities at moderately disturbed sites have high species composition and diversity.

**Relationship between environmental variables and diversity metrics such as species diversity, species richness and evenness**

In ecological studies, it is crucial to measure diversity metrics such as species diversity, richness, and evenness to gain insight into community dynamics and their response to biotic and abiotic factors [47, 89].

In this study, topographic factors and disturbances are important determinants of species diversity, species richness, and evenness distribution [90]. In our study, altitude had a significant impact on the species richness and diversity of the Arjo-Diga forest. Species richness and species diversity showed a significant relationship with altitude [8, 9], therefore, species richness and species diversity decrease linearly with increasing altitude. This is consistent with other studies in which species richness and species diversity decreased monotonically along elevation gradients[91]. This shows the strong dependence of species richness and diversity on altitude. Our results are consistent with several previous studies in Ethiopia [84, 92, 93]. At high altitudes, the combination of extreme environmental conditions and selective filters leads to a reduction in species richness and species diversity. Only the plant species best adapted to these conditions can survive and reproduce.

The present study found a significant relationship between slope gradient and species diversity and evenness. We observed that the Shannon–Wiener index (H) and evenness began to decrease at the slope gradient level (30-50°), which was due to the deterioration of soil nutrient spatial conditions and root distribution with increasing slope gradient (see Fig. 6). Therefore, slope is considered to play a crucial role in determining species diversity and evenness, as argued by various authors, such as [94, 95]. Other studies, such as [95, 96], have shown that slope gradients have a significant impact on species diversity and evenness by altering important ecological factors such as humidity, heat, light intensity and soil conditions. However, high species diversity and evenness have been observed on steep slopes in Semen Mountain National Park [80], as there is less human disturbance on steep slopes.

Disturbance plays a crucial role in shaping species diversity, richness, and evenness in the current study. The research findings indicate that species richness and diversity exhibit an increasing trend with rising levels of human disturbance, reaching a peak at a certain threshold. However, beyond this threshold, species richness and diversity start to decline (Fig 6). This pattern is attributed, in part, to the emergence of disturbance-tolerant and fast-growing ruderal species during moderate disturbances. These results are consistent with findings from other studies [97-99], which consistently demonstrate that species richness and biodiversity are generally higher under moderate disturbance levels but tend to decrease under severe disturbance conditions.

In addition, in the studied forest coffee plantation and management practices, a reduction in tree species richness and diversity were observed. This is because people tend to select smaller trees for logging, while larger trees are needed for shading coffee under the canopy. Similar studies by [55, 97, 98] in southwestern Afromontane forests have reported that the selective removal of economically important tree species and grazing by livestock could contribute to low species richness and diversity in a given forest. Additionally, significant edge effects were observed, with various forms of human disturbance being more noticeable closer to agricultural edges in the study forest. This was expected, as people in the study area heavily rely on the forest for ecosystem services such as wild coffee and spice plantations like *Piper capense* and *Aframomum corrorima*. These findings align with [99, 100], who reported that people living near forests are largely dependent on them for provisioning ecosystem services such as wild coffee, spices, firewood, and honey.

## Correlation between environmental variables

Pearson correlation analysis showed that altitude is positively correlated with soil organic carbon (SOC), with SOC increasing with altitude. The increase in SOC with altitude is likely due to a combination of factors, including temperature, precipitation, topography, and vegetation cover, all of which may contribute to organic matter accumulation [105]. Our results are consistent with results [106, 107] reporting that the SOC in the Himalayan forest is positively correlated with altitude. However, a study by [108] found that the SOC in Nepalease National Park forest decreased with increasing altitude. This could be due to lower decomposition associated with organic carbon production and long-term accumulation. Therefore, this study confirms that altitude is an effective indicator of forest soil organic carbon content.

Moreover, a positive correlation was observed between Cation Exchange Capacity (CEC) and altitude. This association could be explained by the higher vegetation density at higher altitudes, which leads to increased organic matter production and, consequently, higher CEC[109]. Similarly, a study on tea plantations in Indonesia reported that the addition of organic matter to the soil can improve soil CEC[110]. However, some authors[111] have reported a negative correlation between CEC and SOC, as decreased decomposability of Soil Organic Matter (SOM) leads to a decrease in CEC.SOC and total nitrogen (TN) exhibit a positive correlation due to their common origin from organic matter. Nitrogen is released as SOM decomposes, resulting in higher TN content in soils with high SOC. These results align with the findings of [112].Furthermore, there is a negative correlation between soil pH and SOC, as soil pH plays a crucial role in regulating the diversity of microbial communities that affect the rate of SOC degradation[110].By understanding the interrelationships among these soil properties,

appropriate soil management practices can be implemented to enhance soil fertility and support diverse plant communities.

# Conclusion

The aim of the study was to investigate how environmental factors, including elevation, slope, soil nutrients, and disturbance, influence species richness, diversity, and plant communities in the Arjo-Diga forest in western Ethiopia. The results revealed that the forest exhibits high biodiversity, including endemic species, highlighting the urgent need for immediate protection to prevent irreversible loss. Plant community diversity exhibited variations and was associated with environmental factors such as soil nutrients, disturbance, and topography. Notably, the *Acacia abyssinica-Millettia ferruginea* community displayed the lowest species richness and diversity indices, emphasizing the importance of conservation efforts in this community. Altitude was identified as a significant factor impacting species diversity and distribution, with fewer species occurring at lower elevations due to anthropogenic disturbances such as fuelwood extraction, selective logging, and the harvesting of non-timber forest products. Soil nutrients and anthropogenic disturbances also influenced the species composition and diversity of plant communities. The study recommended further research to investigate the effects of climate change on plant community formation and species distribution, the impact of slope aspect on floristic composition and diversity, and the consequences of land-use change on vegetation cover.

# Acknowledgments

The authors would like to express their sincere gratitude to the Diga District Agriculture and Rural Development Office for providing valuable information that significantly contributed to the successful completion of this study. They would also like to extend their thanks to the staff of the Ethiopian National Herbarium at Addis Ababa University for their invaluable assistance in identifying the specimens. Additionally, the authors wholeheartedly appreciate the support and cooperation received from the local authorities and community of the Diga district, as their involvement greatly facilitated the accomplishment of this study.

# References


1. Dakhil, M.A., et al., *Richness patterns of endemic and threatened conifers in south-west China: topographic-soil fertility explanation.* Environmental Research Letters, 2021. **16**(3): p. 034017.
2. Gebrehiwot, K., et al., *Elevational changes in vascular plants richness, diversity, and distribution pattern in Abune Yosef mountain range, Northern Ethiopia.* Plant diversity, 2019. **41**(4): p. 220-228.
3. Naud, L., et al., *Altitude effects on spatial components of vascular plant diversity in a subarctic mountain tundra.* Ecology and evolution, 2019. **9**(8): p. 4783-4795.
4. Costanza, J.K., et al., *Classifying forest inventory data into species-based forest community types at broad extents: exploring tradeoffs among supervised and unsupervised approaches.* Forest Ecosystems, 2018. **5**(1).
5. Nepali, B.R., J. Skartveit, and C.B. Baniya, *Impacts of slope aspects on altitudinal species richness and species composition of Narapani-Masina landscape, Arghakhanchi, West Nepal.* Journal of Asia-Pacific Biodiversity, 2021. **14**(3): p. 415-424.
6. Liu, Y., et al., *Effects of contemporary environment and Quaternary climate change on drylands plant diversity differ between growth forms.* Ecography, 2019. **42**(2): p. 334-345.
7. Pandey, K., Y. Adhikari, and M. Weber, *Structure, composition and diversity of forest along the altitudinal gradient in the Himalayas, Nepal.* Appl Ecol Environ Res, 2016. **14**(2): p. 235-251.
8. Gairola, S., et al., *Species richness and diversity along an altitudinal gradient in moist temperate forest of Garhwal Himalaya.* Am J Sci, 2009. **5**: p. 119-128.
9. Trigas, P., et al. *Elevational gradient of vascular plant species richness and endemism in Crete*. in *Poster 248 at the Fifth Biennial Conference of the International Biogeography Society, Irakleion, Crete, Greece*. 2011.
10. Ahmad, M., et al., *Patterns of plant communities along vertical gradient in Dhauladhar Mountains in Lesser Himalayas in North-Western India.* Science of The Total Environment, 2020. **716**: p. 136919.
11. Bennie, J., et al., *Influence of slope and aspect on long-term vegetation change in British chalk grasslands.* Journal of ecology, 2006. **94**(2): p. 355-368.
12. Fadrique, B. and J. Homeier, *Elevation and topography influence community structure, biomass and host tree interactions of lianas in tropical montane forests of southern Ecuador.* Journal of vegetation science, 2016. **27**(5): p. 958-968.
13. Waring, B.G., J.M. Becknell, and J.S. Powers, *Nitrogen, phosphorus, and cation use efficiency in stands of regenerating tropical dry forest.* Oecologia, 2015. **178**(3): p. 887-897.
14. Fisher, J.B., et al., *Nutrient limitation in rainforests and cloud forests along a 3,000-m elevation gradient in the Peruvian Andes.* Oecologia, 2013. **172**(3): p. 889-902.
15. Stevens, C.J., et al., *Impact of nitrogen deposition on the species richness of grasslands.* Science, 2004. **303**(5665): p. 1876-1879.
16. Huston, M., *Soil nutrients and tree species richness in Costa Rican forests.* Journal of Biogeography, 1980: p. 147-157.
17. Nadeau, M.B. and T.P. Sullivan, *Relationships between plant biodiversity and soil fertility in a mature tropical forest, Costa Rica.* International Journal of Forestry Research, 2015. **2015**.
18. Zhu, Y., et al., *The effects of crop tree thinning intensity on the ability of dominant tree species to sequester carbon in a temperate deciduous mixed forest, northeastern China.* Forest Ecology and Management, 2022. **505**: p. 119893.

19. Shrestha, K.B., et al., *Effect of anthropogenic disturbance on plant species diversity in oak forests in Nepal, Central Himalaya.* International Journal of Biodiversity Science, Ecosystem Services & Management, 2013. **9**(1): p. 21-29.
20. Zhang, J., et al., *Long-term grazing effects on vegetation characteristics and soil properties in a semiarid grassland, northern China.* Environmental Monitoring and Assessment, 2017. **189**: p. 1-13.
21. Willig, M. and S. Presley, *Biodiversity and disturbance.* The encyclopedia of the Anthropocene, 2018. **3**: p. 45-51.
22. Collins, S.L., S.M. Glenn, and D.J. Gibson, *Experimental analysis of intermediate disturbance and initial floristic composition: decoupling cause and effect.* Ecology, 1995. **76**(2): p. 486-492.
23. Volis, S., *Conservation-oriented restoration–a two for one method to restore both threatened species and their habitats.* Plant Diversity, 2019. **41**(2): p. 50-58.
24. Gebretsadik, T., *Causes for biodiversity loss in Ethiopia: A review from conservation perspective.* South Agricultural Research Institute (SARI), Hawassa Agricultural Research center, 2016. **6**(11).
25. Kebede, M., et al., *Vegetation structural characteristics and topographic factors in the remnant moist Afromontane forest of Wondo Genet, south central Ethiopia.* Journal of forestry research, 2013. **24**: p. 419-430.
26. Dibaba, A., T. Soromessa, and B. Warkineh, *Plant community analysis along environmental gradients in moist afromontane forest of Gerba Dima, South-western Ethiopia.* BMC Ecology and Evolution, 2022. **22**(1): p. 12.
27. Teketay, D. and T. Bekele, *Floristic composition of Wof-Washa natural forest, Central Ethiopia: Implications for the conservation of biodiversity.* Feddes Repertorium, 1995. **106**(1-5): p. 127-147.
28. Siraj, M., et al., *Floristic composition and plant community types in Maze National Park, southwest Ethiopia.* Applied Ecology and Environmental Research, 2017. **15**(1): p. 245-262.
29. Jaleta, G. and H. Jebssa *The impact of large scale agriculture on forest and wildlife in Diga Woreda, Western Ethiopia.* Asian Journal of Agriculture, 2017. **1**(02): p. 100-113.
30. Jaleta, O.G. and H. Jebessa *The impact of large scale agriculture on forest and wildlife in Diga Woreda, Western Ethiopia.* Asian Journal of Agriculture, 2017. **1**(02): p. 100-113.
31. Mohr, P.A., *The geology of Ethiopia*. 1971: Haile Selassie I University Press.
32. Scholte, P., *Atlas of the Potential Vegetation of Ethiopia. Ib Friis, Sebsebe Demissew & Paulo van Breugel. Copenhagen: The Royal Danish Academy of Sciences and Letters in Biologiske Skrifter58. 2010. 307 pp. including 29 colour map plates (1: 2 000 000). ISSN 0366 3612, ISBN 97887 7304 347 9. 400 DKK (approx.£ 46) paperback.* Edinburgh Journal of Botany, 2012. **69**(1): p. 207-209.
33. Mueller-Dombois, D. and H. Ellenberg, *Aims and methods of vegetation ecology*. 1974: Wiley.
34. Van der Maarel, E., *Transformation of cover-abundance values in phytosociology and its effects on community similarity.* Vegetatio, 1979. **39**(2): p. 97-114.
35. Nelson, D.a. and L.E. Sommers, *Total carbon, organic carbon, and organic matter.* Methods of soil analysis: Part 2 chemical and microbiological properties, 1983. **9**: p. 539-579.
36. Skjemstad, J. and R. Reeve, *The determination of nitrogen in soils by rapid high-temperature Kjeldahl digestion and autoanalysis.* Communications in Soil Science and Plant Analysis, 1976. **7**(3): p. 229-239.
37. Ryti, R., *On the determination of soil pH.* Agricultural and Food Science, 1965. **37**(1): p. 51-60.
38. Gutierrez Boem, F.H., G. Rubio, and D. Barbero, *Soil phosphorus extracted by Bray 1 and Mehlich 3 soil tests as affected by the soil/solution ratio in Mollisols.* Communications in soil science and plant analysis, 2011. **42**(2): p. 220-230.

39. Chapman, H., *Cation-exchange capacity.* Methods of soil analysis: Part 2 Chemical and microbiological properties, 1965. **9**: p. 891-901.
40. Anderson, E.W. and W.F. Currier, *Evaluating zones of utilization.* Rangeland Ecology & Management/Journal of Range Management Archives, 1973. **26**(2): p. 87-91.
41. Yeshitela, K. and T. Bekele, *Plant community analysis and ecology of Afromontane and transitional rainforest vegetation of southwestern Ethiopia.* SINET: Ethiopian Journal of Science, 2002. **25**(2): p. 155-175.
42. Legendre, P. and L. Legendre, *Numerical ecology*. 2012: Elsevier.
43. Voigt, W., *PC-ORD Reviews.* ESA Bulletin. **92**(3): p. 298-301.
44. Team, R.C., *R: A language and environment for statistical computing. R Foundation for Statistical Computing, Vienna, Austria.* http://www. R-project. org/, 2016.
45. Mbuga, F. and C. Tortora, *Spectral clustering of mixed-type data.* Stats, 2021. **5**(1): p. 1-11.
46. Maechler, M., et al., *Cluster: cluster analysis basics and extensions.* 2012.
47. Magurran, A.E., *Measuring biological diversity.* Current Biology, 2021. **31**(19): p. R1174-R1177.
48. Sorensen, T.A., *A method of establishing groups of equal amplitude in plant sociology based on similarity of species content and its application to analyses of the vegetation on Danish commons.* Biol. Skar., 1948. **5**: p. 1-34.
49. Kent, M., *Vegetation description and data analysis: a practical approach*. 2011: John Wiley & Sons.
50. Chao, A., C.-H. Chiu, and L. Jost, *Unifying species diversity, phylogenetic diversity, functional diversity, and related similarity and differentiation measures through Hill numbers.* Annual review of ecology, evolution, and systematics, 2014. **45**: p. 297-324.
51. Field, A., *Discovering statistics using IBM SPSS statistics*. 2013: sage.
52. Gole, T.W., *Vegetation of the Yayu forest in SW Ethiopia: impacts of human use and implicatiions for in situ conservation of wild Coffea arabica L. Populations*. Vol. 10. 2003: Cuvillier Verlag.
53. Assefa, A., S. Demissew, and Z. Woldu, *Floristic composition, structure and regeneration status of M asha forest, south-west E thiopia.* African Journal of Ecology, 2014. **52**(2): p. 151-162.
54. Burju, T., K. Hundera, and E. Kelbessa, *Floristic composition and structural analysis of Jibat humid afromontane forest, west Shewa zone, Oromia national regional state, Ethiopia.* Ethiopian Journal of Education and Sciences, 2013. **8**(2): p. 11-34.
55. Gurmessa, F., T. Soromessa, and E. Kelbessa, *Floristic Composition and Community Analysis of Komto Afromontane Moist Forest, East Wollega Zone, West Ethiopia.* Science, Technology and Arts Research Journal, 2013. **2**(2): p. 58-69.
56. Gebrehiwot, K. and K. Hundera, *Species composition, plant community structure and natural regeneration status of Belete moist evergreen montane forest, Oromia regional state, Southwestern Ethiopia.* Momona Ethiopian Journal of Science, 2014. **6**(1): p. 97-101.
57. Bilew, A., H. Kitessa, and A. Balcha, *Floristic composition and structural analysis of Gelesha forest, Gambella regional State, Southwest Ethiopia.* Journal of Ecology and the Natural Environment, 2015. **7**(7): p. 218-227.
58. Gemechu, T., E. Kelbessa, and T. Soromessa, *Floristic Composition and Community analysis of Gendo Moist Montane Forest of East Wellega, Western Ethiopia.* Journal of Natural Sciences Research, 2015. **5**: p. 15.
59. Admassu, A., et al., *Floristic composition and plant community types of Agama Forest, an â€ œAfromontane Forestâ€ in Southwest Ethiopia.* Journal of Ecology and the Natural Environment, 2016. **8**(5): p. 55-69.

60. Kelbessa, E. and T. Soromessa, *Interfaces of regeneration, structure, diversity and uses of some plant species in Bonga Forest: A reservoir for wild coffee gene pool.* SINET: Ethiopian Journal of Science, 2008. **31**(2): p. 121-134.
61. Song, X., et al., *Different environmental factors drive tree species diversity along elevation gradients in three climatic zones in Yunnan, southern China.* Plant Diversity, 2021. **43**(6): p. 433-443.
62. Kelbessa, E. and S. Demissew, *Diversity of vascular plant taxa of the flora of Ethiopia and Eritrea.* Ethiopian Journal of Biological Sciences, 2014. **13**(Supp.)): p. 37-45.
63. Gupta, R.K., *Features of Afro-Alpine Plant Ecology.(Acta Phytogeographica Succica 49)*. 1965, JSTOR.
64. Garg, S., R.K. Joshi, and S.C. Garkoti, *Effect of tree canopy on species composition, diversity and biomass of herbaceous vegetation and soil characteristics in semi-arid forests of the Aravalli hills.* 2020.
65. Addi, A., et al., *Floristic composition and plant community types of Agama Forest, an Afromontane Forest in Southwest Ethiopia.* Journal of Ecology and the Natural Environment, 2016. **8**(5): p. 55-69.
66. Wakjira, F.S., *Biodiversity and ecology of Afromontane rainforests with wild Coffea arabica L. populations in Ethiopia*. 2006: Cuvillier Verlag.
67. Addi, A., T. Soromessa, and T. Bareke, *Floristic composition and vegetation community analysis of Gesha and Sayilem Forest in Kaffa Zone south western Ethiopia.* Biodiversitas Journal of Biological Diversity, 2020. **21**(7).
68. Maharjan, S.K., et al., *Temperature and soils predict the distribution of plant species along the Himalayan elevational gradient.* Journal of Tropical Ecology, 2022. **38**(2): p. 58-70.
69. Mulugeta, Y., T. Bekele, and E. Kelbessa, *Floristic Composition, Species Diversity and Vegetation Structure of Gera Moist Montane Forest, Jimma Zone of Oromia National Regional State, Southwest Ethiopia.* Ethiopian Journal of Biological Sciences, 2015. **14**(1): p. 45-68.
70. Muleta, D., et al., *Socioeconomic benefits of shade trees in coffee production systems in Bonga and Yayuhurumu districts, southwestern Ethiopia: farmers' perceptions.* Ethiopian Journal of Education and Sciences, 2011. **7**(1): p. 39-55.
71. Hundera, K., *Shade tree selection and management practices by farmers in traditional coffee production systems in Jimma Zone, Southwest Ethiopia.* Ethiopian Journal of education and sciences, 2016. **11**(2): p. 91-105.
72. Gemechu, H.W., D. Lemessa, and D.B. Jiru, *A comparative analysis of indigenous and exotic tree species management practices in agricultural landscapes of Southwest Ethiopia.* Trees, Forests and People, 2021. **4**: p. 100059.
73. Senbeta, F., et al., *Plant diversity, vegetation structure and relationship between plant communities and environmental variables in the Afromontane Forests of Ethiopia.* SINET: Ethiopian Journal of Science, 2014. **37**(2): p. 113-130.
74. Alebachew, G.W., T.A. Abejew, and Z. Tesema, *Honeybee Colony Management Practices and Identification of Honeybee Floras in Eastern Amhara, Ethiopia.* Journal of Apiculture, 2022. **2**: p. 151-161.
75. Jamil, A., et al., *Impact of human settlements on diversity of range vegetation.* Sustainability, 2022. **14**(1): p. 519.
76. Somashekar, R., M.B. Raj, and B. Nagaraja. *Impact of human settlement on forest structure and composition in Kudremukh national park, Western Ghats, south India*. in *XII World Forestry Congress, Quebec City*. 2003.

77. Yao, Z., et al., *Precipitation and temperature regulate species diversity, plant coverage and aboveground biomass through opposing mechanisms in large-scale grasslands.* Frontiers in Plant Science, 2022. **13**: p. 999636.
78. Socolar, J.B., et al., *How should beta-diversity inform biodiversity conservation?* Trends in ecology & evolution, 2016. **31**(1): p. 67-80.
79. Hao, M., et al., *Assessing biological dissimilarities between five forest communities.* Forest Ecosystems, 2019. **6**(1): p. 1-8.
80. Wondie, M., et al., *Relationship between topographic variables and land cover in the Simen Mountains National Park, a World Heritage Site in northern Ethiopia.* International Journal of Remote Sensing Applications, 2012. **2**(2): p. 36-43.
81. Kebede, M., et al., *Soil seed bank and seedlings bank composition and diversity of Wondo Genet moist Afromontane forest South Central Ethiopia.* International Journal of Botany, 2012. **8**(4): p. 170-180.
82. Teshome, M., Z. Asfaw, and M. Mohammed, *Diversity Influences Tree Aboveground Carbon Stocks Through Functional Diversity and Functional Dominance in the Dry Evergreen Afromontane Forest of Hararghe Highland, Southeast Ethiopia.* 2020.
83. Djordjević, V., et al., *Patterns of distribution, abundance and composition of forest terrestrial orchids.* Biodiversity and Conservation, 2020. **29**(14): p. 4111-4134.
84. Rawal, R.S., et al., *Plant species diversity and rarity patterns along altitude range covering treeline ecotone in Uttarakhand: conservation implications.* Trop. Ecol, 2018. **59**(2): p. 225-239.
85. Zheng, X., X. Wei, and S. Zhang, *Tree species diversity and identity effects on soil properties in the Huoditang area of the Qinling Mountains, China.* Ecosphere, 2017. **8**(3): p. e01732.
86. Bekele, T., *Phytosociology and ecology of a humid Afromontane forest on the central plateau of Ethiopia.* Journal of Vegetation Science, 1994. **5**(1): p. 87-98.
87. Collins, S.L. and S.M. Glenn, *Intermediate disturbance and its relationship to within-and between-patch dynamics.* New Zealand Journal of Ecology, 1997: p. 103-110.
88. Fakhry, A.M. and G.S. Aljedaani, *Impact of disturbance on species diversity and composition of Cyperus conglomeratus plant community in southern Jeddah, Saudi Arabia.* Journal of King Saud University-Science, 2020. **32**(1): p. 600-605.
89. Maestre, F.T., et al., *Plant species richness and ecosystem multifunctionality in global drylands.* Science, 2012. **335**(6065): p. 214-218.
90. Sharma, P., et al., *Floristic diversity and distribution pattern of plant communities along altitudinal gradient in Sangla Valley, Northwest Himalaya.* The Scientific World Journal, 2014. **2014**.
91. Grytnes, J.A. and O.R. Vetaas, *Species richness and altitude: a comparison between null models and interpolated plant species richness along the Himalayan altitudinal gradient, Nepal.* The American Naturalist, 2002. **159**(3): p. 294-304.
92. McCain, C.M. and J.-A. Grytnes, *Elevational gradients in species richness.* eLS, 2010.
93. Meragiaw, M., et al., *Floristic composition and structure of the Kibate Forest along environmental gradients in Wonchi, Southwestern Ethiopia.* Journal of Forestry Research, 2021: p. 1-14.
94. Boll, T., et al., *Spatial distribution and environmental preferences of the piassaba palm Aphandra natalia (Arecaceae) along the Pastaza and Urituyacu rivers in Peru.* Forest Ecology and Management, 2005. **213**(1-3): p. 175-183.
95. Zeng, X.H., et al., *Slope aspect and slope position have effects on plant diversity and spatial distribution in the hilly region of Mount Taihang, North China.* J. Food Agric. Environ., 2014. **12**(1): p. 391-397.

96. ZHANG, C.-s., et al., *Effects of topographic factors on the plant species richness and distribution pattern of alpine meadow in source region of Lancang River, Southwest China.* Chinese Journal of Ecology, 2012. **31**(11): p. 2767.
97. Senbeta, F. and M. Denich, *Effects of wild coffee management on species diversity in the Afromontane rainforests of Ethiopia.* Forest Ecology and Management, 2006. **232**(1-3): p. 68-74.
98. Shumi, G., et al., *Woody plant use and management in relation to property rights: a social-ecological case study from southwestern Ethiopia.* Ecosystems and People, 2019. **15**(1): p. 303-316.
99. Hundera, K., et al., *Effects of coffee management intensity on composition, structure, and regeneration status of Ethiopian moist evergreen afromontane forests.* Environmental management, 2013. **51**: p. 801-809.
100. Shumi, G., et al., *Conservation value of moist evergreen Afromontane forest sites with different management and history in southwestern Ethiopia.* Biological conservation, 2019. **232**: p. 117-126.
101. Beche, D., et al., *Spatial variation in human disturbances and their effects on forest structure and biodiversity across an Afromontane forest.* Landscape Ecology, 2022. **37**(2): p. 493-510.
102. Huston, M.A., *Disturbance, productivity, and species diversity: empiricism vs. logic in ecological theory.* Ecology, 2014. **95**(9): p. 2382-2396.
103. Li, W., et al., *Effects of different grazing disturbances on the plant diversity and ecological functions of alpine grassland ecosystem on the qinghai-Tibetan plateau.* Frontiers in Plant Science, 2021: p. 2909.
104. Yirga, F., et al., *Impact of altitude and anthropogenic disturbance on plant species composition, diversity, and structure at the Wof-Washa highlands of Ethiopia.* Heliyon, 2019. **5**(8): p. e02284.
105. Schultz, H., *Soil, vine, climate change; the challenge of predicting soil carbon changes and greenhouse gas emissions in vineyards and is the 4 per 1000 goal realistic? This article is published in cooperation with Terclim 2022 (XIVth International Terroir Congress and 2nd ClimWine Symposium), 3-8 July 2022, Bordeaux, France.* OENO One, 2022. **56**(2): p. 251-263.
106. Devi, S.B. and S.S.S.S. Sherpa, *Soil carbon and nitrogen stocks along the altitudinal gradient of the Darjeeling Himalayas, India.* Environmental monitoring and assessment, 2019. **191**: p. 1-18.
107. Kumar, A., et al., *Forest soil nutrient stocks along altitudinal range of Uttarakhand Himalayas: An aid to Nature Based Climate Solutions.* Catena, 2021. **207**: p. 105667.
108. Shapkota, J. and G. Kafle, *Variation in soil organic carbon under different forest types in Shivapuri Nagarjun National Park, Nepal.* Scientifica, 2021. **2021**.
109. Kilambo, D.L., et al., *Effect of soils properties on the quality of compact arabica hybrids in Tanzania.* American Journal of Research Communication, 2015. **3**(1): p. 15-19.
110. Gebrehiwot, K., et al., *Soil organic carbon stock in Abune Yosef afroalpine and sub-afroalpine vegetation, northern Ethiopia.* Ecological Processes, 2018. **7**(1): p. 1-9.
111. Zhang, X.-N., et al., *Influence of edaphic factors on plant distribution and diversity in the arid area of Xinjiang, Northwest China.* Arid land research and Management, 2018. **32**(1): p. 38-56.
112. Fang, K., et al., *Decreased soil cation exchange capacity across northern China's grasslands over the last three decades.* Journal of Geophysical Research: Biogeosciences, 2017. **122**(11): p. 3088-3097.

S1 Appendix. Floristic list of species in the Arjo-Diga forest. (DOCX)

Figure

Click here to access/download;Figure;Fig 1.tiff(1).tif

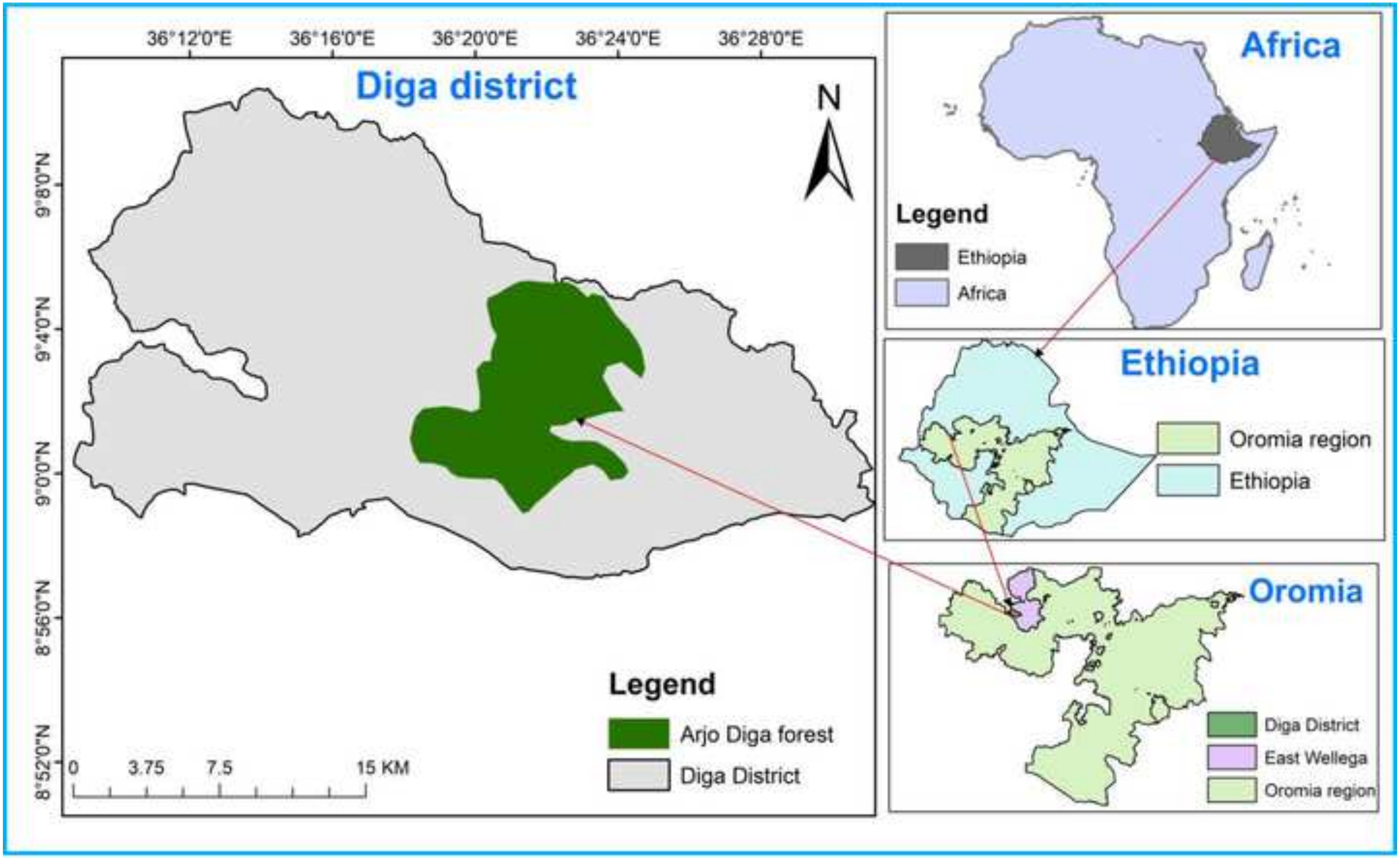


**Fig 1. Fig 1: Map of Africa showing Ethiopia, the Oromia region, and the study district**

**(Source: OCHA, 2021) (https://data.humdata.org/dataset/cod-ab-eth)**

Click here to access/download;Figure;Fig 2..tif

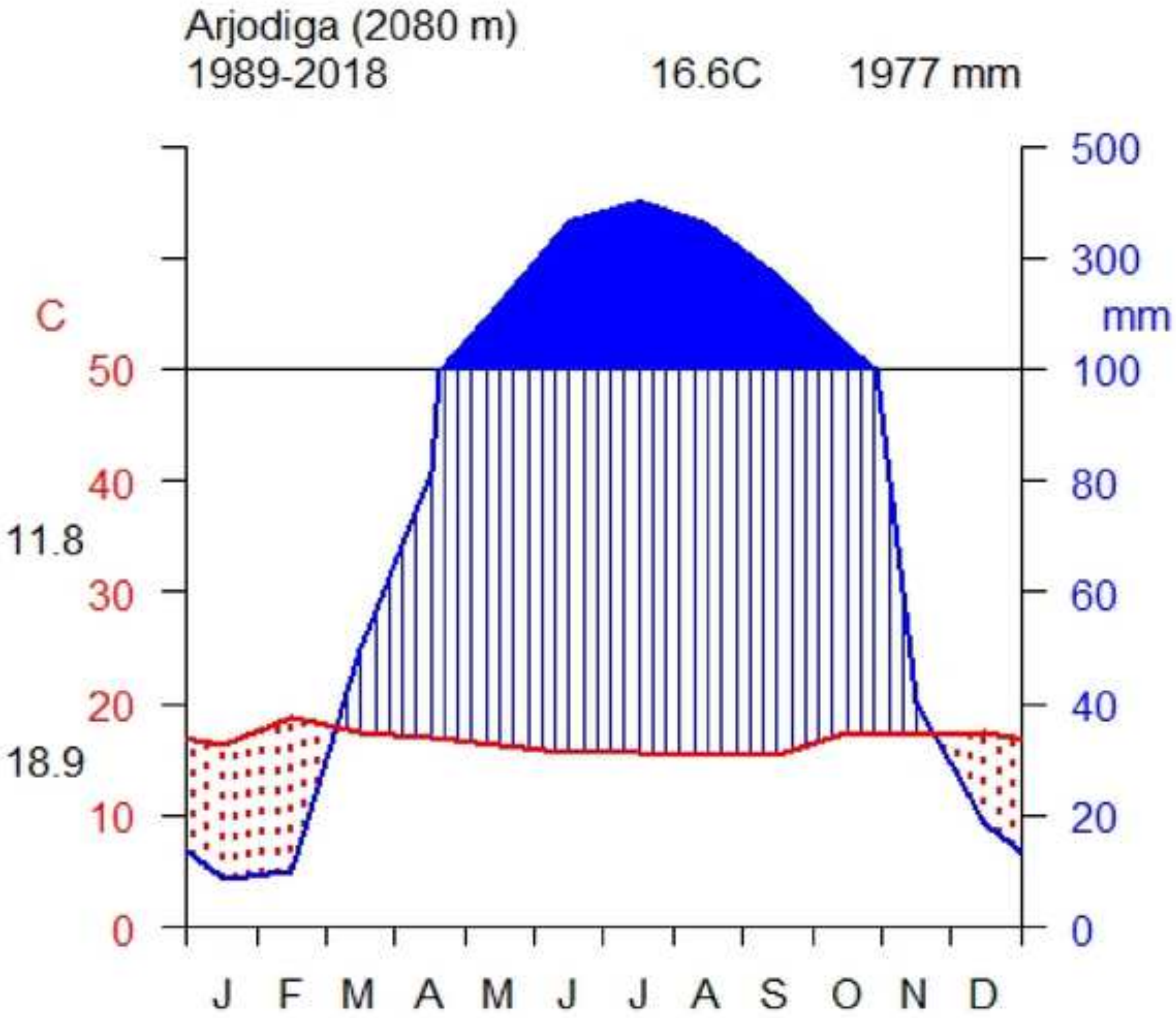


**Fig 2. Climate diagram of study area**

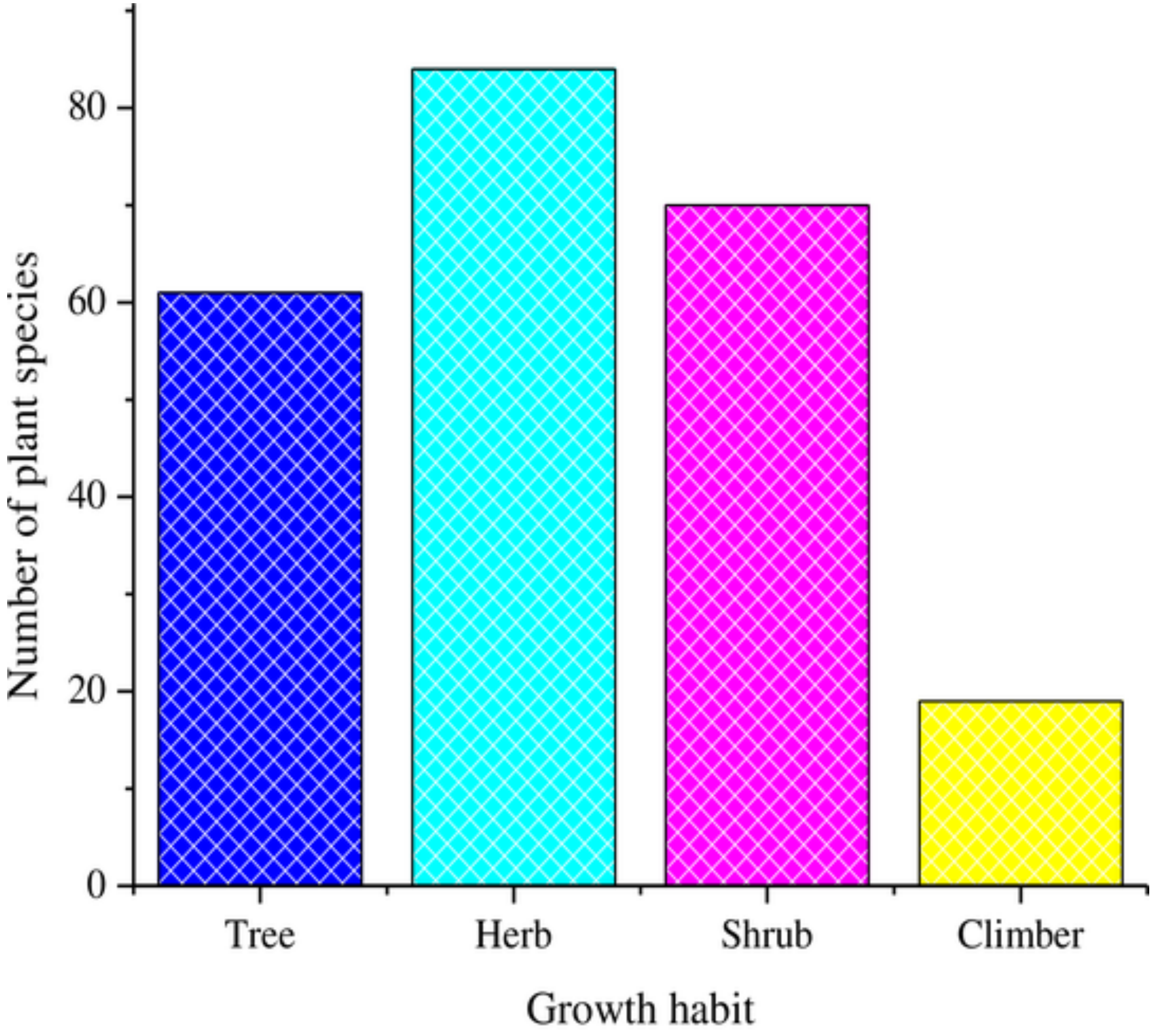


**Fig 3. Growth habit of plant**

Figure Click here to access/download;Figure;Fig 4.(2).tif

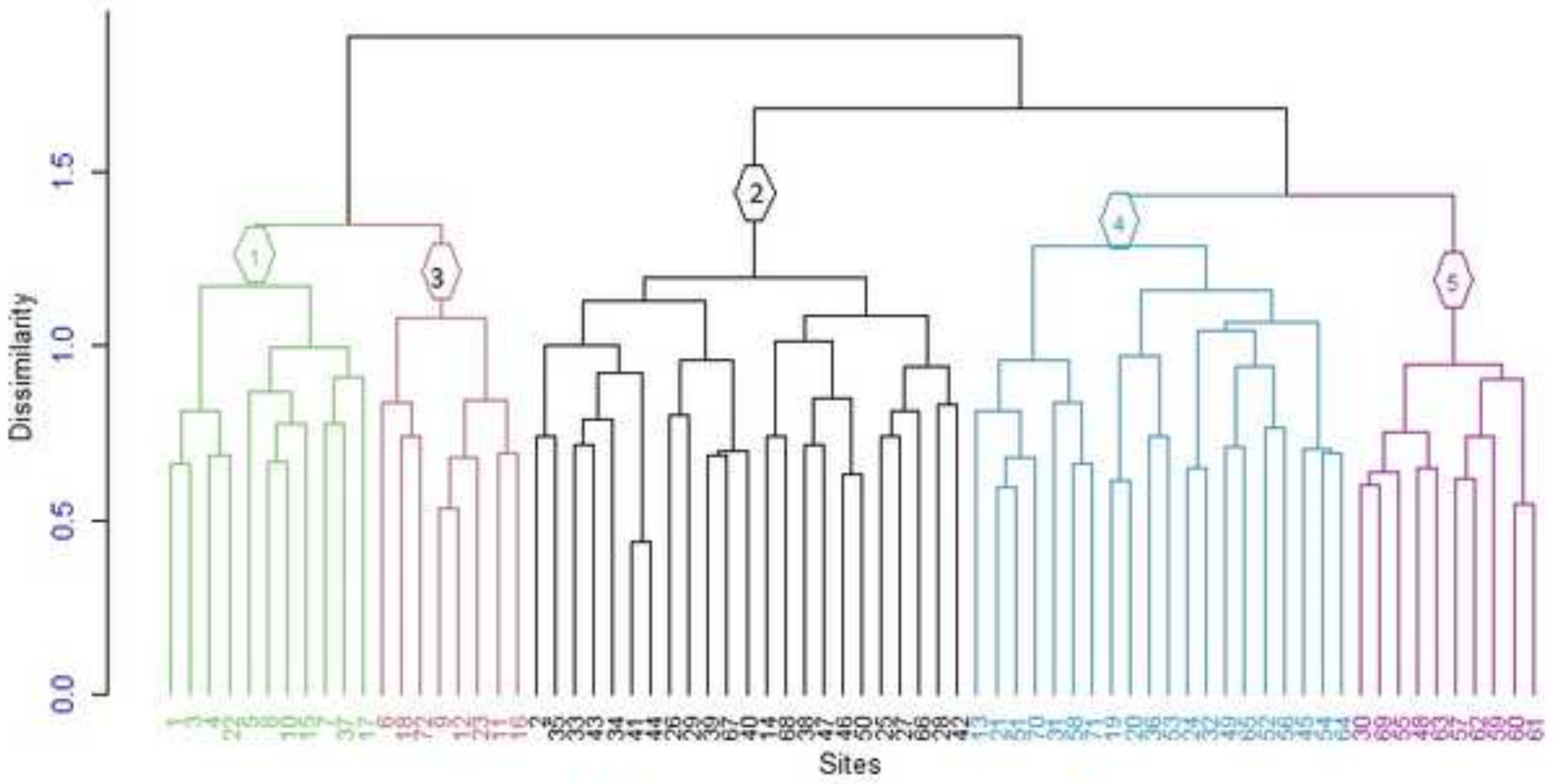


**Fig 4. Dendrogram generated from agglomerative hierarchical cluster analysis of vegetation data. 1= community type 1, 2= community type 2, 3= community type 3, 4= community type 4, and 5 =community type 5.**

 Click here to access/download;Figure;Fig 5(1).tif

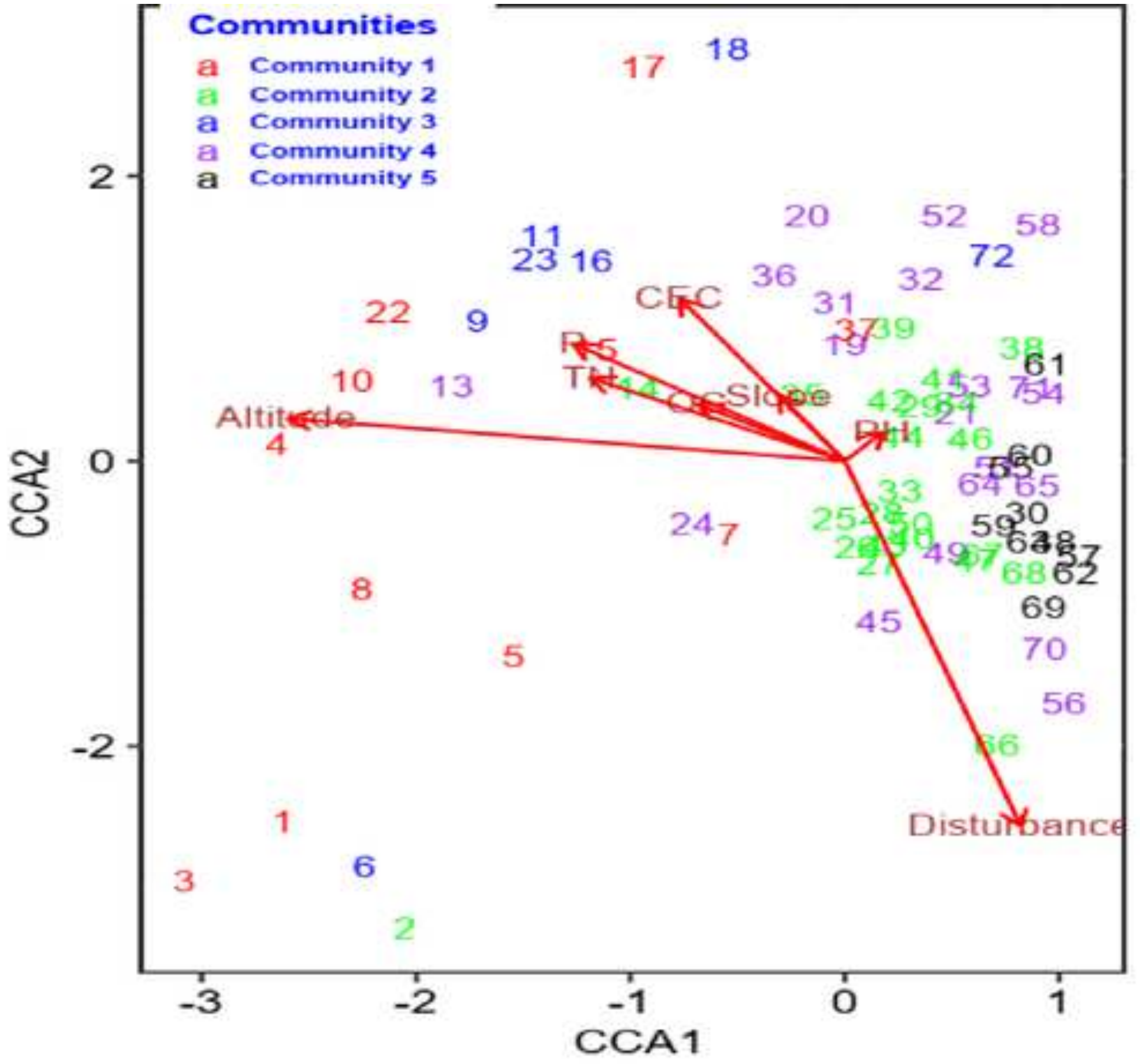


**Fig 5. The CCA ordination diagram displays 72 plots, five environmental variables, and five plant communities**

Figure Click here to access/download;Figure;Fig 6. Figs 6A,6B,6C, and 6D(1).tif

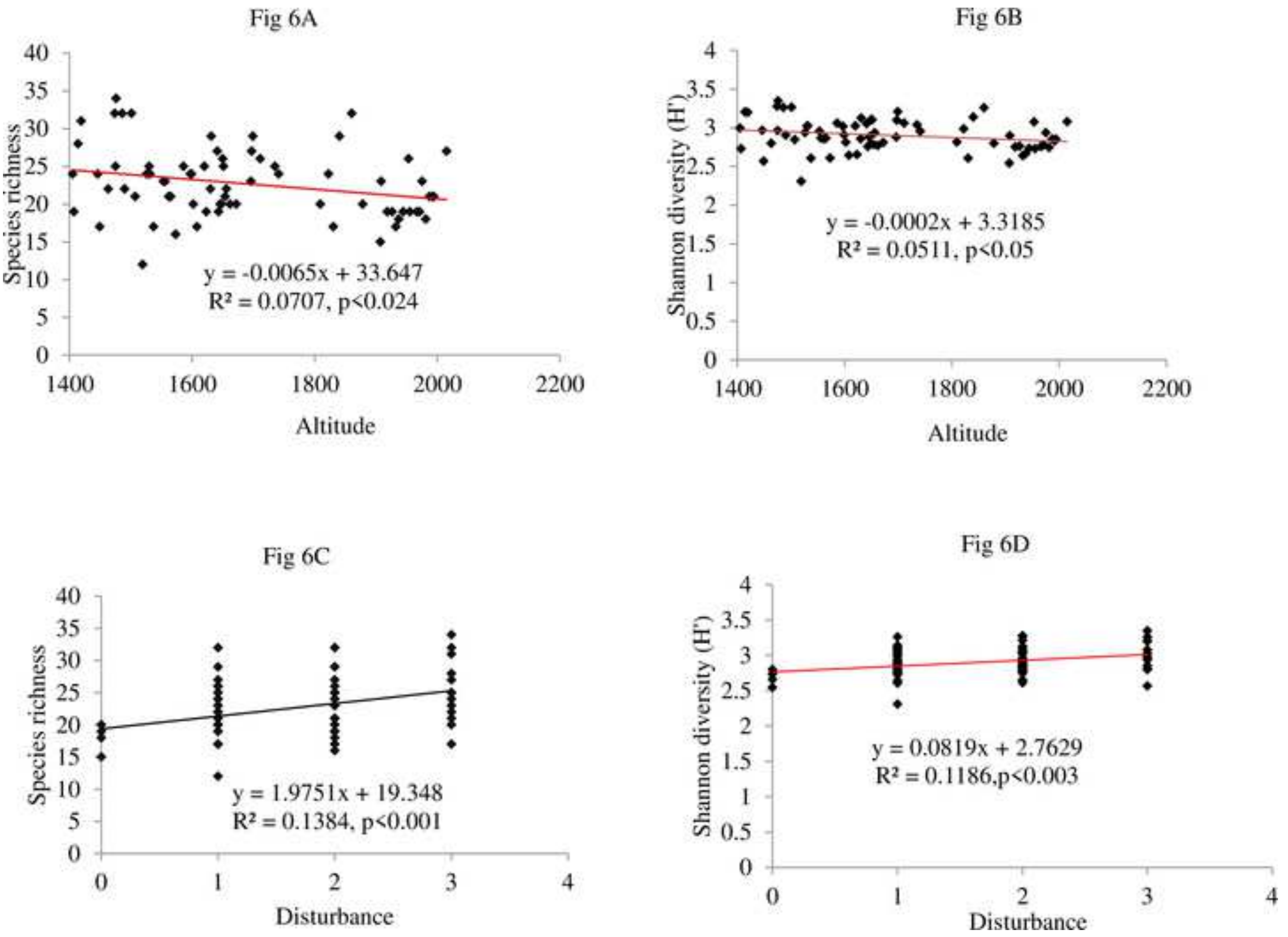

Figure

Click here to access/download;Figure;Fig 6. Figs 6E,6F,6G, and 6H(1).tif

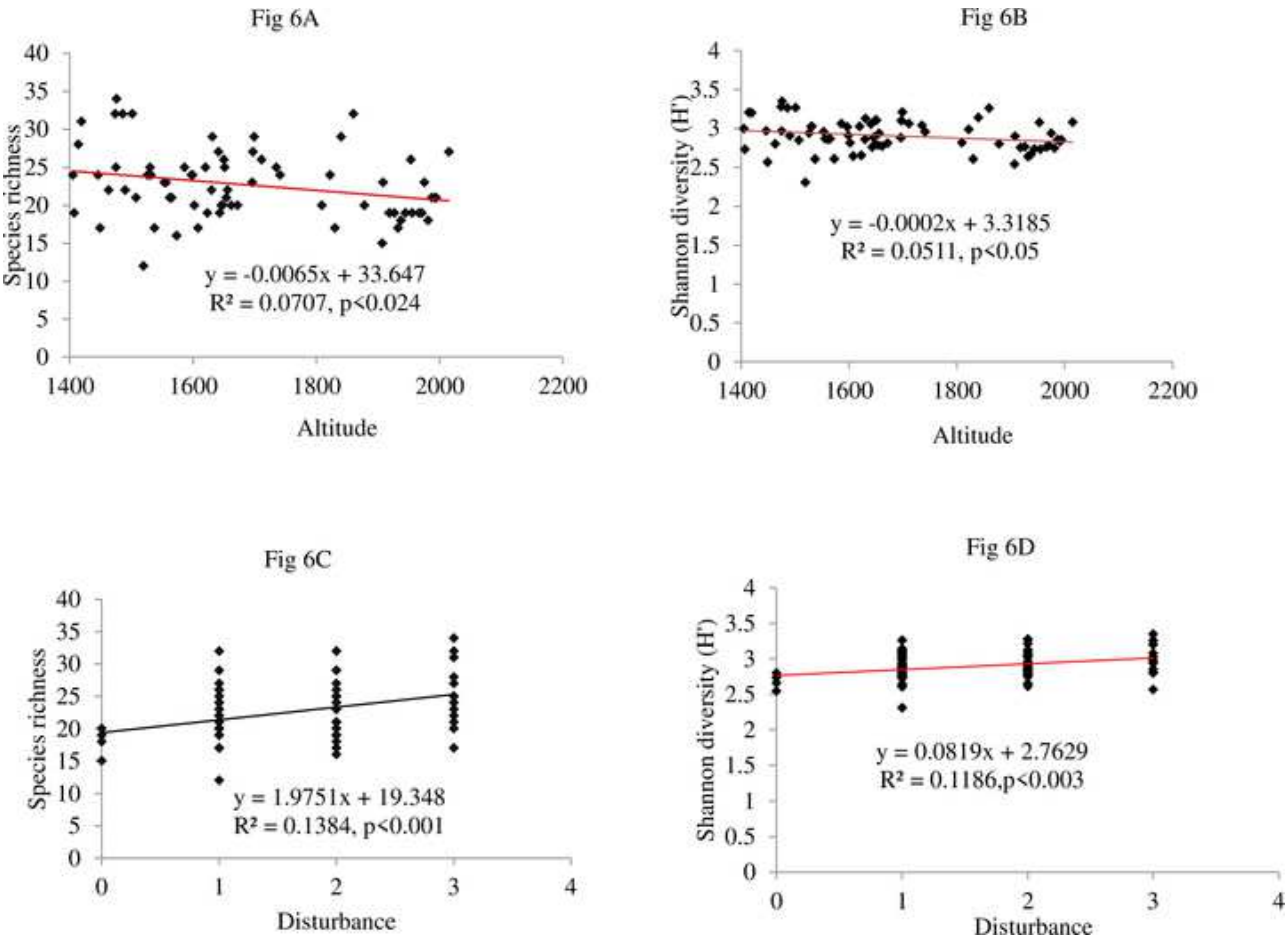

Figure

Click here to access/download;Figure;Fig 7.Figs 7A,7B,7C, and 7D.tif

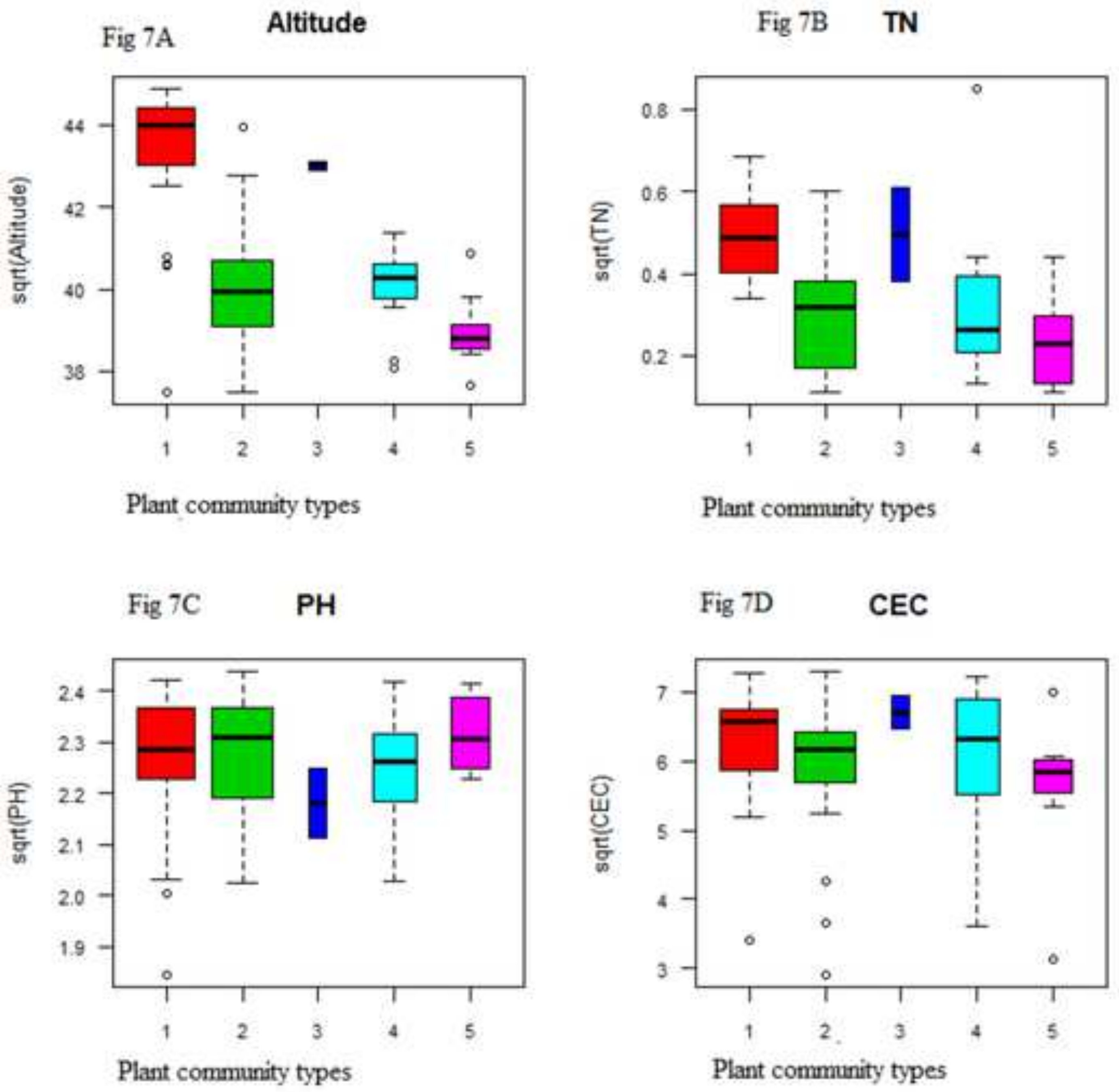

Figure
Click here to access/download;Figure;Fig. 7.figs 7E,7F,7G,and H.tif

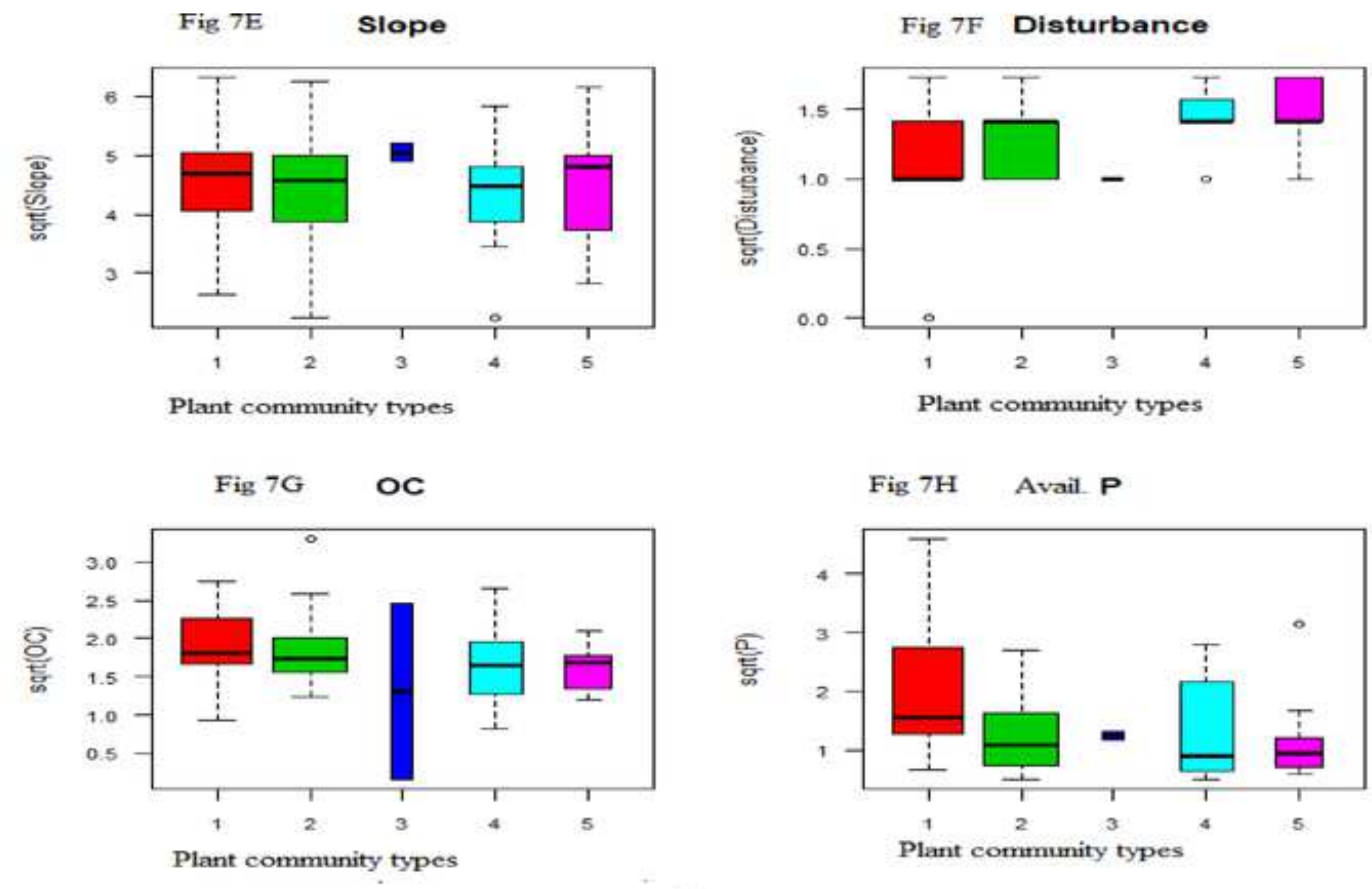


**Fig 7. Illustrates the influence of environmental variables on different plant community types: Community type 1:** ***Maesa lanceolata-Ehretia cymosa*****, Community type 2:** ***Trichilia dregeana-Ficus vasta*****, Community type 3:** ***Acacia abyssinica-Millettia ferruginea*****, Community type 4:** ***Combertum collinum-Bersama abyssinica*****, and Community type** ***5:Terminalia macroptera-Piliostigma thonninigii***

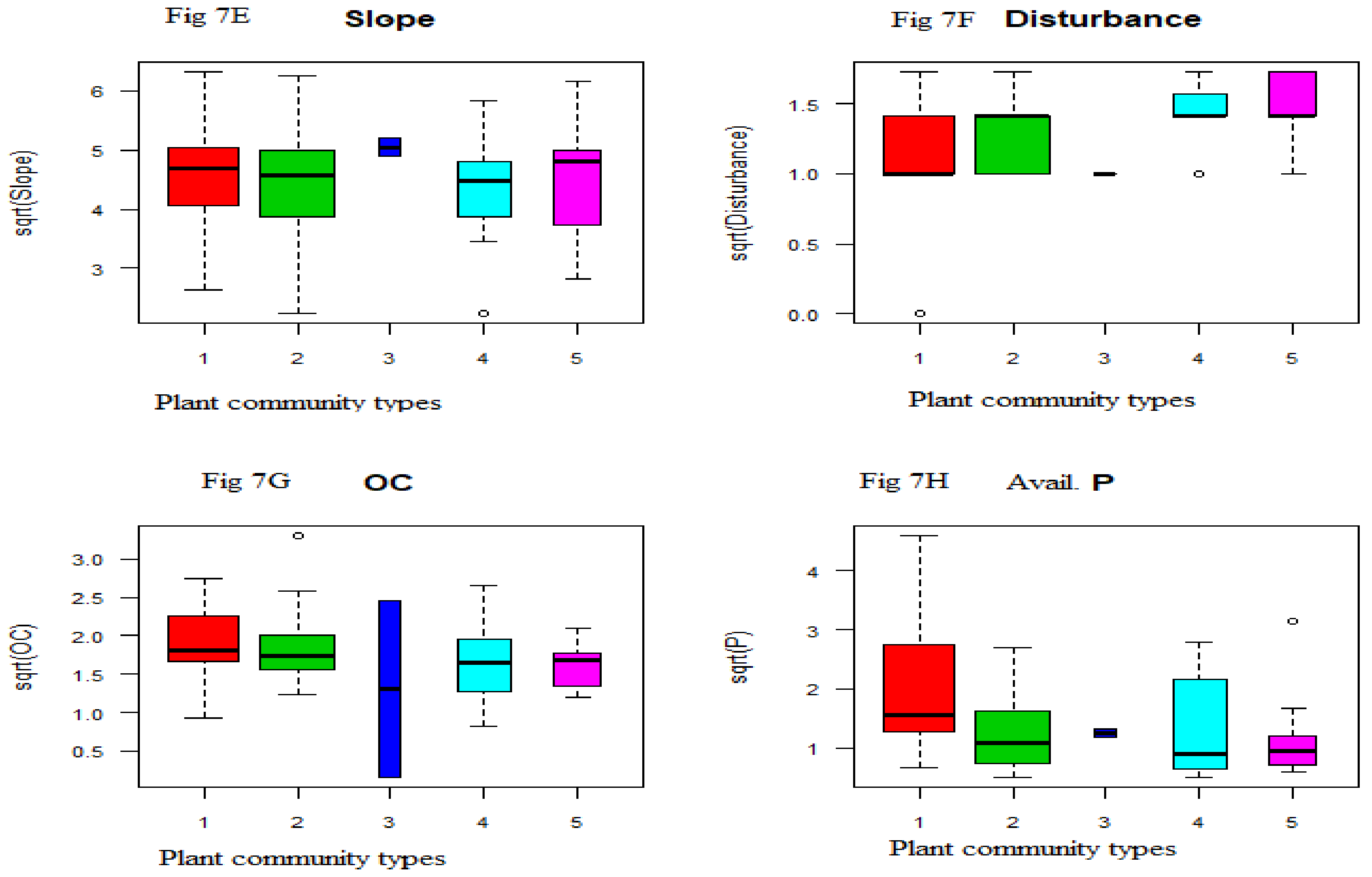


**Fig 7. Illustrates the influence of environmental variables on different plant community types: Community type 1: *Maesa lanceolata-Ehretia cymosa*, Community type 2: *Trichilia dregeana-Ficus vasta*, Community type 3: *Acacia abyssinica-Millettia ferruginea*, Community type 4: *Combertum collinum-Bersama abyssinica*, and Community type *5:Terminalia macroptera-Piliostigma thonninigii***

Click here to access/download
**Supporting Information**
Supporting information.docx

Click here to access/download
**Other**
Response of editor.docx

# ***Topography factors and soil variables drive the plant community distribution pattern and species richness in Arjo-Diga Forest in Western Ethiopia.***

Tariku Berihun*¶[1], Tamrat Bekele¶[2], Ermias Lulekal¶[2], and Zemede Asfaw¶[2]

[1]Department of Biology, Dilla University, Dilla, Ethiopia

[2]Department of Plant Biology and Biodiversity Management, Addis Ababa University, Addis Ababa, Ethiopia

*Corresponding Author. E-mail: berihun.tariku@yahoo.com

¶These authors contributed equally to this work

**Abstract**

This study aimed to examine the influence of topography and soil variables on plant community distribution patterns and species richness in the Arjo-Diga Forest. A total of 72 plots, each measuring 900 $m^2$, were established along altitudinal gradients to collect vegetation data.~~ Trees and shrubs with a diameter at breast height (DBH) of 2.5 cm were recorded in each plot. Herb and soil data were also collected from five subplots, measuring 1 $m^2$, located at the corners and center of each plot~~. Agglomerative cluster analysis and Canonical Correspondence Analysis (CCA) using R software were employed to identify plant communities and analyze the relationship between plant community types and environmental variables. In this study, Asteraceae, Fabaceae, and Euphorbiaceae were the dominant families, represented by 17 genera and 29 species (12.4%), 16 genera and 23 species (9.8%), and 10 genera and 14 species (6%), respectively. The agglomerative hierarchical cluster analysis identified five plant communities: *Maesa lanceolata-Ehretia cymosa*, *Trichilia dregeana-Flacourtia indica*, *Acacia abyssinica-Millettia ferruginea*, *Combretum collinum-Croton macrostachyus*, and *Terminalia macroptera-Piliostigma thonninigii*. The CCA results demonstrated significant relationships between species

**Commented [H1]:** W bring this sentence before plant communities based reviewer comment

distribution variations and environmental factors, including altitude, disturbance, total nitrogen, and cation exchange capacity (CEC). The findings suggest that species richness and diversity in each plant community (communities 1, 2, 3, 4, and 5) are directly influenced by altitude, cation exchange capacity, total nitrogen, slope, and disturbances, with altitude being the most influential factor. The study also revealed the significant impacts of anthropogenic factors such as human settlement, expansion of agricultural land, and livestock grazing on the plant communities' species distribution patterns. Therefore, the study highlights the urgent need for sustainable practices to protect species diversity and endemic species in forest ecosystems. In conclusion, this study provides valuable insights into the floristic diversity and plant community types in the Arjo-Diga forest. It emphasizes the importance of sustainable forest management plans that consider the impact of human activities on species diversity and the formation of plant communities.

Keywords: Environmental variables, anthropogenic factors, species diversity, plant communities

# Introduction

Investigating the relationship between vegetation attributes, such as richness, diversity, and evenness, and environmental factors is crucial for designing effective conservation activities [1]. The interactions among various environmental factors can lead to changes in habitat conditions, which in turn directly or indirectly affect species distribution and plant diversity worldwide. The composition, diversity, and spatial distribution patterns of plant species are influenced by both abiotic and biotic factors [2]. Understanding the complex relationship between plants and abiotic factors is essential, and further research into their influence on species distribution and the formation of plant communities is urgently needed [3].

Most studies of environmental factors have focused on elevation, topography, and soil to study how environmental factors affect species composition and plant diversity in different ecosystems. Altitude, in particular, drastically alters abiotic factors such as water, temperature, soil composition, and area, which in turn affect plant growth and development [4, 5]. Altitude also regulates the response of plant communities to environmental factors on global scale [6]. A study by Pandey et al. [7] found different patterns of species richness along different elevation gradients. Several studies have shown that species richness patterns decrease monotonically from lowest to highest elevation [8, 9] or monotonically increase. Hump-shaped patterns have also been reported at mid-elevations [2, 10]. However, some researchers have shown that species richness is low at mid-elevations [11]. These contrasting results suggest that species richness along elevation gradients is not a general trend since many species exhibit different phenotypic traits, such as different leaf characteristics, biomass, and phenology. As a result, patterns of species richness and distribution of plant species along altitudinal gradients often differ in ways that are important to conservationists.

Soil nutrients play a crucial role in shaping vegetation structure and plant diversity by creating heterogeneous habitats. Deficiencies in soil nutrients have been reported to impact various aspects of forest ecosystems in tropical forests, including community structure, plant biomass, tree height, and basal area [12]. Conversely, forests with high species richness often exhibit high nutrient dynamics in certain locations. The influence of soil factors, such as total nitrogen (TN) and available phosphorus, on plant community structure appears to be well-established [13]. Total nitrogen (TN), in particular, acts as a limiting factor for plant growth [14] and significantly

**Formatted:** Font color: Red

**Commented [H2]:** Abbreviation forme writen

influences plant diversity and community composition. Additionally, studies have demonstrated that species richness can be influenced by high nitrogen deposition [15]. In tropical forests, soil cation exchange capacity (CEC) has been found to impact tree species richness [16, 17]. However, there are still knowledge gaps that need to be addressed in order to fully understand how soil cation exchange capacity (CEC) affects tree species richness in tropical forests.

Species composition, diversity, and distribution are also influenced by anthropogenic disturbances such as agricultural expansion, settlement, overgrazing of livestock, selective cutting, and fires [18]. Disturbance has a positive effect on vegetation properties to a certain extent [19]. However, disturbances usually have a negative effect on vegetation since the relationship between vegetation features and disturbances is often negative [20, 21]. Under conditions of severe disturbance, species richness and diversity are low because most species cannot tolerate frequent destructive events. However, due to dominant competitors and fast colonizers, high species richness can be predicted at moderate levels of disturbance [22]. It is important to understand how species respond to human-caused disturbances to make informed conservation decisions. This understanding enables effective actions such as habitat conservation and restoration necessary to maintain critical ecological phenomena such as species distribution limits.[23].

Various authors have documented the results of studies on biodiversity, structure, and regeneration status in different moist Afromontane forests in different parts of Ethiopia. However, many of their species are threatened, endangered, or locally extinct due to habitat destruction, fragmentation, and overexploitation of forest products and habitats [24].

**Commented [H3]:** Abbreviation form written

**Commented [H4]:** New references cite.

**Commented [H5]:** New cited references based on reviewer's comment

Understanding the interaction between species diversity and composition and the relationship between environmental factors and plant community types remains critical to the development of forest management strategies.

The Arjo-Diga forest is a complex ecosystem influenced by a variety of local and regional environmental factors. Factors such as soil nutrients, disturbance and microclimate are critical at the local scale and have significant impacts on the distribution pattern of species in the region. Soil properties and disturbances can affect species distribution and composition, while human activities such as logging and grazing have negative impacts on species diversity and composition. Similar results have been reported in other parts of southwestern Ethiopia [25, 26] and demonstrate the key role of soil nutrients such as total nitrogen, cation exchange capacity and soil organic carbon in floristic variation. In addition, natural disturbances, such as fires, can create opportunities for new species to emerge [27, 28]. It is important to understand that these factors are important for forest management to promote healthy and diverse plant communities and mitigate the negative impacts of human activities.

The Arjo-Diga forest, facing degradation from both human and man-made factors, was designated as a biodiversity conservation area in 2014. Despite its protected status, the forest continues to be exploited by the surrounding population for activities such as agricultural expansion, timber extraction, and charcoal production. Unfortunately, no studies have been conducted to assess species composition or analyze plant communities in these areas. Therefore, the present study aims to fill this knowledge gap by conducting a plant community analysis across environmental gradients and investigating the influence of environmental factors on plant diversity, including richness, diversity, and evenness.

Specifically, the objectives of this study were (1) to examine the floristic composition and diversity of the Arjo-Diga forest, (2) to assess the impact of environmental variables on plant diversity characteristics (such as richness, diversity, and evenness), and (3) to explore the relationship between plant community types and environmental variables.

# Materials and methods

## Study area

The Arjo-Diga forests are situated in southwestern Ethiopia and encompass a total area of 12,683.6 hectares. The elevation in the region ranges from 1,200 to 2,220 meters above sea level (m.a.s.l.). Specifically, the forests are located in Diga District, within the East Wollega Zone of the Oromia Regional State. They are positioned 12 km away from Nekemte town and approximately 340 km from Addis Ababa, the capital of Ethiopia. Geographically, the forests are situated between 9°59′00″ and 9°6′30″ north latitude and 36°18′30″ and 36°24′30″ east longitude (Fig 1). The forests are bounded by three districts: Guto Gida, Chewaka, Sasiga, and Leka Dulecha.

**Commented [H6]:** Abbreviation of meter above se level

The topography of the area exhibits variability, ranging from flat and gentle slopes to moderate and steep slopes. Slope gradients range from 7% to 30% in the southwest and from 30% to 60% in the northeast, making the region more susceptible to severe erosion. According to [29], the study area comprises two agroecological zones: the lowlands (51.4%) and the midlands (48.6%). The midlands are characterized by steep slopes and are predominantly covered by forests.

**Fig 1. Map of Africa showing, Ethiopia, the Oromia region and the study district (Source: OCHA, 2021(https://data.humdata.org/dataset/cod-ab-eth)**

## Soil and Geology

Acriosols are the dominant soil types in the study area These soils are heavily weathered and acidic, with a low cation exchange capacity [30]. They are characterized by a low pH value and insufficient phosphorus content. The dominant soil colors observed were red in the midlands and black in the lowlands. Geologically, the study area comprises Precambrian rocks of high origin, including migmatites [31].

## Climate

The study area mainly experiences tepid to cool subhumid mid-highlands in the northeastern part and hot to warm humid lowlands in the northwestern part. The maximum temperature was 25.7 °C, and the minimum temperature was 0.2 °C (Fig 2). The mean annual precipitation is approximately 1977 mm/year with a unimodal precipitation pattern. The monthly mean precipitation trend shows its maximum in July, June and August and is dry from November and extends into January.

**Fig 2. Climate diagram of study area**

## Vegetation types

~~According to [32], ~~The vegetation type of the Arjo-Diga forest ~~vegetation ~~is part of the humid Afromontane forest and is characterized by broadleaf evergreen species[32].This vegetation is found at an altitude between 1500 and 2342 ~~meters above sea level~~m.a.s.l. with characteristic emergent species, including *Pouteria adolf-friederici*. Other distinctive species in this forest are *Sapium ellipticum, Celtis africana, Euphorbia ampliphylla, Ficus sur, Schefflera abyssinica, Syzygium guineense ssp. afromontanum, Nuxia congesta, Galiniera saxifraga, Rytigynia Neglecta, Vepris dainellii, Rothmannia urcelliformi* and several *Albizia* species [32]. *Combretum*

*paniculatum*, *Landolphia buchananii* and *Urera hypselodendron* were the dominant woody climbers in the forest. The western part of the study area borders the Didessa valley, which forms the *Combretum-Terminalia* vegetation.

## ~~Ethical statement~~

~~Ethical approval was obtained from the Diga District Department of Agriculture in Ethiopia to conduct this study. The study forest is public and protected to protect biodiversity as part of a climate-resilient green economy. We confirm that all national and international regulations were observed during field work. It is customary to enter the sampling sites and collect plant and soil samples after explaining the aim of the research and submitting the proposal for village development agents (DAs), which was approved by the Diga District Office of Agriculture. We confirmed that the field study plant collection did not include all endangered or protected species.~~

**Commented [H7]:** We agreed to delete the ethics statement based on the reviewer's comment, as the idea is to reflect permission to collect soil and plant samples from the study area.

# Methods of data collection

## Vegetation data collection

A reconnaissance survey was conducted from October 2010 to January 2013 to select sampling sites and to decide on the possible number of transect lines to be laid down. After a reconnaissance survey, nine transect lines were systematically laid along an altitudinal gradient from 1360 to 2220 ~~m above sea level~~m.a.s.l. from the crest of the base. A total of 72 sampling plots, each measuring 900 m2 (30 m x 30 m), were sampled to record woody species Vegetation data were collected from a total of 72 plots along nine transects that were systematically constructed using the sampling approach described by [33]. Each plot was relocated every 100

meters along the transect lines. Of the 72 plots, a single plot measuring 30 m x 30 m (900 m$^2$) was created to record tree species. Within each of the main plots, five 5 m x 5 m (25 m$^2$) subplots were created to record shrubs species. Within each 25 m$^2$ subplot, five 2 m x 2 m (4 m$^2$) subplots were created, one in the center and four at the corner of the plot, to record herbaceous species. Finally, the percentage cover of each species in each plot was visually estimated and later converted to a 1–9 modified Braun-Blanquet scale [34]. Voucher specimens in each plot were collected, coded, pressed, and dried. Using the Flora of Ethiopia and Eritrea (volumes 1–8), all voucher specimens was identified and have been deposited in the National Herbarium of Ethiopia, Addis Ababa University.

## Environmental Data

~~The environmental variables altitude~~ Altitude, latitude and longitude for each plot were recorded~~of each plot~~ ~~were taken~~ using ~~a handheld~~ Garmin GPS H72. The slope of sample plot was measured using Suunto clinometer~~and clinometer, respectively~~. Soil samples were ~~collected~~ taken from these 72 sample plot ~~-~~for soil analysis .The soil samples were taken a depth of 0 to ~~with a soil core sampler at a depth of~~ 30 cm. Soil samples were taken from each plot at five locations, one in the center and ~~the other~~ four in ~~at each~~the corner, and these mixed to ~~produce~~ obtain a composite sample and then one sample was~~s~~ taken for analysis. ~~The~~ Samples were air dried, ground, and passed through a 2 mm sieve to remove rock fragments and large root particles prior to analysis. Soil samples were analyzed at the Bedele's soil laboratory for selected soil chemistry including soil pH, organic carbon, available phosphorus, total nitrogen ~~(TN)~~ and cation exchange capacity  Soil organic carbon was determined using the dichromate oxidation method [35] and total soil nitrogen was analyzed using the Kjeldahl digestion and distillation

method [36]. Soil~~The~~ pH ~~of the soil~~ was determined ~~with~~ using a pH meter at a soil- to- liquid ratio of 1:2.5 [37], available phosphorus was extracted using the Bray-1 method [38] and using a spectrophotometer and CEC was determined using the following ammonium acetate method [39].

In each main plot, we examined various forms of human disturbance, including firewood collection, the presence of beehives, charcoal production, timber extraction, footpath creation, and expansion of agricultural land through deforestation, the presence of coffee cultivation, tree debarking, and grazing. To assess the level of human intervention, we recorded indicators such as the presence or absence of stumps, logs, grazing intensity, and signs of firewood collection. We estimated the intensity of forest grazing by visually observing its impact on livestock, including manure droppings and grass clippings. Additionally, we examined the distance between each plot and the forest edge, as well as the distance to nearby houses. To categorize the different types of disturbances, we qualitatively ranked them based on the remaining evidence observed in each plot. We assigned ratings of relatively absent (indicating no disorder), low, medium, or high, with corresponding values of 0, 1, 2, and 3, respectively, for each level of disturbance[40, 41]. By summing the results for each plot, we created an overall ranking of human disturbance in each community. Higher ranks indicate higher levels of human disturbance, while lower ranks indicate lower levels of disturbance.

## Ordination

For this study, we employed Detrended Correspondence Analysis (DCA) as the chosen ordination method to analyze vegetation data. The selection of the best ordination methods was based on the criteria that if the longest axis length of the DCA exceeds 4, it indicates



**Commented [H8]:** Modified paragraph based on reviewer comment

heterogeneity [42]. In this study, the longest axis length observed was 4.2 SD, confirming the presence of heterogeneous data. Consequently, we decided to utilize a unimodal technique and opted for canonical correspondence analysis (CCA) to illustrate the relationships between plant communities, plots, and environmental variables The study considered a total of 72 plots, 234 species, and 8 environmental variables, which included elevation, slope, pH, available phosphorus, total nitrogen (TN), cation exchange capacity (CEC), disturbances, and soil organic carbon (SOC).

## Data analysis

### Plant community classification

Cluster analysis is a useful tool for grouping observations, such as plots, based on their floristic similarities, specifically the number of plant species present in each plot [43]. In this study, a data matrix consisting of 72 plots and 234 plant species was employed for hierarchical cluster analysis, which allows for the grouping of plant species based on their similarities. For the cluster analysis in this study, an agglomerative hierarchical approach was utilized, employing Ward's method and a similarity ratio. This method helps classify vegetation into distinct plant community types based on species abundance. We utilized the free statistical software R version 4.2.22 [44] along with the Cluster and Dendextend packages [45] to perform the cluster analysis.

An indicator species analysis (ISA) was conducted using the indicator value (Indval) approach to identify diagnostic plant species for the classification of plant community types. To perform this analysis, several packages were utilized, including Vegan, Cluster, and Labdsv [46].The indicator values of each species were evaluated based on the probability of indicator values across specific iterations. In this analysis, plant species that exhibited a statistically significant

indicator value at $p<0.05$ were considered indicator species for a particular plant community type. Ultimately, two of the most dominant plant species within each cluster were selected to assign names to the community types.

## Species diversity

The Shannon diversity index and Shannon evenness were calculated to assess the species diversity, richness, and evenness of the study area and plant communities[47].

$$H' = -\sum_{i=1}^{s} \text{Pi} \ln \text{Pi} \qquad (1)$$

where H'= Shannon diversity index; S=number of species, pi=proportion of ith species. ln= the natural logarithm.

The Shannon evenness index (J) was calculated by using:

$$J = \frac{H'}{H'\text{max}} \qquad (2)$$

where $H'$= Shannon–Wiener diversity index and Hmax = lns, where s is the number of species in the plot.

Sorensen's similarity coefficient (Ss) was used to compare the similarity between two communities [48, 49].

$$Ss = \frac{2a}{(2a+b+c)} \qquad (3)$$

where 'a' represents the number of species that are present in both communities, 'b' represents the number of species unique to the first community, and 'c' represents the number of species unique to the second community[50].

## Ordination

Ordination is a multivariate analysis method in ecology that uses an ordination plot to study the relationships among species, plots, plant communities, and environmental variables in a two-dimensional space [42]. Pearson's correlation coefficient was used to calculate correlations between environmental variables using SPSS version 20 software [51]. For this analysis, canonical correspondence analysis (CCA) was used to test relationships between species, plant communities, and environmental variables. In addition, multiple linear regression models were used to test the relationship between environmental variables and diversity attributes at $p<0.05$. Two-way ANOVA followed by a post hoc Tukey HSD test was used to determine if there were significant mean differences between plant communities and environmental variables

# Results

## Floristic composition

A total of 234 species, belonging to 183 genera and 73 families, were documented in this study. The five families with the highest species richness were Asteraceae (29 species), Fabaceae (23 species), Euphorbiaceae (14 species), Lamiaceae (12 species), and Rubiaceae (11 species). In contrast, eight families each had between four and six species (Table 1).The recorded species were classified into different growth forms, including trees, shrubs, herbs, and climbers. Among them, trees accounted for 61 species (26%), shrubs comprised 70 species (30%), herbs constituted 84 species (36%), and climbers represented 19 species (8%) (Fig 3).

Out of the total identified plant species, 20 species (8.5%) were endemic to the flora of Ethiopia and Eritrea (S1 Appendix). Among these species, *Crotalaria rosenii, Impatiens tinctoria, Kalanchoe petitiana*, *Pycnostachys abyssinica*, and *Solanecio gigas* were some of the newly

recorded endemic species in the Wollega floristic region (Table 2). Furthermore, there were approximately 13 species (5.5%) from 10 families and 13 genera that were newly recorded in the Wollega floristic region (Table 2).

**Table 1. Families with more than three genera and their representative number of species**

| Family (N=73) | No of genera (No=183) | No of species (N=234) |
|---|---|---|
| Asteraceae | 17(10%) | 29(12.4%) |
| Fabaceae | 16(8.7%) | 23 (9.8%) |
| Rubiaceae | 11(7%) | 11 (4.7%) |
| Euphorbiaceae | 10(5.5%) | 14 (6%) |
| Lamiaceae | 10(8.7%) | 12(5.1%) |
| Acanthaceae | 6 (2.9%) | 6(2.6%) |
| Poaceae | 6(2.9%) | 6(2.6%) |
| Malvaceae | 4(2.2%) | 5(2.1%) |
| Rhamnaceae | 4(2.2%) | 5(2.1%) |
| Rutaceae | 4(2.2%) | 4(1.7%) |
| Sapindaceae | 4(2.2%) | 6(2.6%) |
| Verbenaceae | 4(2.2%) | 4(1.7%) |
| Vitaceae | 4(2.2%) | 5(2.1%) |

**Fig 3. Growth habit of plant**

**Table 2. Newly discovered species and endemic species in the Wollega floristic region**

| NO | Species name | Family | Ha. | Floristic Region |
|---|---|---|---|---|

| | | | | |
|---|---|---|---|---|
| 1 | *Crepis foetida* | Asteraceae | H | EW, TU,GD,SU,AR,HA |
| 2 | *Crotalaria rosenii** | Fabaceae | S | SU, AR, BA, KF, SD |
| 3 | *Cyperus distans* | Cyperaceae | H | AF, GD,SU,IL,KF,GG,SD |
| 4 | *Euphorbia ampliphylla* | Euphorbiaceae | T | TU, GD, GJ, WU, SU, IL, KF SD,HA |
| 5 | *Impatiens tinctoria** | Balsaminaceae | H | GD, SU, AR KF, SD, BA |
| 6 | *Kalanchoe petitiana** | Crassulaceae | H | EW, GD,WU,SU,AR,BA,HA |
| 7 | *Maytenus arbutifolia* | Celastraceae | T | SU, AR, SD GG |
| 8 | *Olea capensis subsp. Macrocarpa* | Oleaceae | T | GD, SU, AR, IL, KF, SD, BA |
| 9 | *Pycnostachys abyssinica** | Lamiaceae | S | IL ,KF, GG, SD |
| 10 | *Solanecio gigas** | Asteraceae | S | GD, GJ, WU, SU, AR, SD, IL, KF, BA, HA |
| 11 | *Solanum incanum* | Solanaceae | S | EW, EE, TU, AF, SU, GG, SD, BA, AR |
| 12 | *Tagetus minuta* | Asteraceae | H | EW, TU, GD, WU, SU, KF, SD, BA, HA |
| 13 | *Trifolium semiplosum* | Fabaceae | H | EW, TU, GD, WU, SU, AR, BA, SD |

where Ha=habit, H=herb. S=shrub, T=tree, and * stands for newly discovered endemic species.

## Plant community types

In this study, hierarchical cluster analysis was employed to identify five distinct plant community types (Fig 4). The dendrogram was constructed using the ratio and Ward's method, illustrating the relationships between the five clusters based on percent species coverage data. The analysis involved clustering a data matrix consisting of 72 plots and 234 plant species using agglomerative hierarchical cluster analysis. Each community type was named after one or two dominant indicator woody species, chosen based on their significant indicator values and

synoptic mean abundances (Table 4). In this study, a species was considered an indicator of a particular group if its indicator value was statistically significant ($p < 0.05$) (Table 3). The identified plant communities in this study were *Maesa lanceolata-Ehretia cymosa*, *Trichilia dregeana-Ficus vasta*, *Acacia abyssinica-Millettia ferruginea*, *Combretum collinum-Bersama abyssinica*, and *Terminalia macroptera-Piliostigma thonningii*

**Fig 4. Dendrogram generated from agglomerative hierarchical cluster analysis of vegetation data.1= community type 1, 2= community type 2, 3= community type 3, 4= community type 4, and 5=community type 5.**

**Table 3. Value of the indicator species in identified plant communities and their significant p value.**

| Indicator species | Community types | Indicator value | P value |
|---|---|---|---|
| *Ehretia cymosa* | 1 | 0.603 | 0.001*** |
| *Erythrococca trichogyne* | 1 | 0.254 | 0.022* |
| *Euphorbia ampliphylla* | 1 | 0.364 | 0.003* |
| *Pruns africana* | 1 | 0.353 | 0.002* |
| *Tecela noblis* | 1 | 0.202 | 0.05* |
| *Bersama abyssinica* | 2 | 0.284 | 0.033* |
| *Cyathula cylindrica* | 2 | 0.217 | 0.026* |
| *Flacourtia indica* | 2 | 0.282 | 0.04* |
| *Trichilia dregeana* | 2 | 0.551 | 0.004* |
| *Acaccia abyssinica* | 3 | 0.874 | 0.001*** |
| *Buddleja polystachya* | 3 | 0.24 | 0.022* |
| *Girardinia diversifolia* | 3 | 0.218 | 0.025* |

| | | | |
|---|---|---|---|
| *Grewia bicolar* | 3 | 0.366 | 0.003* |
| *Lippia adoensis* | 3 | 0.208 | 0.029* |
| *Millettia ferruginea* | 3 | 0.22 | 0.055* |
| *Vernonia auriculifera* | 3 | 0.279 | 0.032* |
| *Combertum collinum* | 4 | 0.276 | 0.044* |
| *Gnidia glauca* | 4 | 0.229 | 0.021* |
| *Maytenus obscura* | 4 | 0.568 | 0.003* |
| *Guizotia villosa* | 4 | 0.164 | 0.049* |
| *Combretum molle* | 5 | 0.262 | 0.01* |
| *Ficus exasperata* | 5 | 0.376 | 0.003* |
| *Ficus vasta* | 5 | 0.276 | 0.015* |
| *Flueggea virosa* | 5 | 0.425 | 0.002* |
| *Gardenia ternifolia* | 5 | 0.563 | 0.001*** |
| *Grewia ferruginea* | 5 | 0.203 | 0.055* |
| *Piliostigma thonningii* | 5 | 0.704 | 0.001*** |
| *Stereospermum kunthianum* | 5 | 0.341 | 0.005** |
| *Syzygium guineense.subsp.afromontanum* | 5 | 0.446 | 0.001*** |
| *Terminalia macroptera* | 5 | 0.599 | 0.001*** |

Significance codes * = ($p < 0.5$), ** = ($p < 0.01$), *** = ($p < 0.001$)

1. *Maesa lanceolata-Ehretia cymosa* community type

This particular community type is situated at the highest elevation within the study areas, ranging from 1608 to 1992 m.a.s.l. It encompasses eight plots and comprises a total of 110 species.

Among the observed tree species, notable dominants include *Albizia gummifera*, *Celtis africana*, *Millettia ferruginea*, and *Prunus africana*. In the herbaceous layer, the dominant species consist of *Agratum conzoides, Bidens pilosa, Achyranthus aspra*, and *Digitaria abyssinica*. Common lianas/climbers found in this community include *Clematis simensis, Dioscorea praehensilis, Phytolaca decandra, Rhamnus prinoides, Landolphia buchananii, Cyphostemma cyphopetalum*, and *Urera hypselodendron*. The community exhibits five indicator species with significant indicator values ($p < 0.05$) (Table 3). Additionally, this community is characterized by the presence of economically important species such as *Coffea arabica, Dioscorea praehensilis*, and *Piper capense*. *Pteridium aquilinum*, a dominant fern species, exclusively occurs within this community.

2. *Trichilia dregeana* – *Ficus vasta* community type

This community was found between 1537 and 1631 ~~meters above sea level~~m.a.s.l and included 23 plots and 148 species. *Syzygium guineense subsp. guineense, Ficus vasta* and *Croton macrostachyus* were the dominant tree species along with *Trichilia dregeana* and *Flacourtia indica.* Other associated tree species are *Apodytes dimidiata, Combertum collinum, Ficus mucuso, Warburgia ugandensis* and *Cordia africana*. It formed three indicator species, namely, *Cyathula cylindrical, Flacourtia indica* and *Bersama abyssinica*, with significant indicator values ($p<0.05$) (Table 3). *Maytenus gracilipes* and *Carisa spinarum* were the dominant shrub species. The herb layer was dominated by *Oplismenus hirtellus, Laggera crispata* and *Kalanchoe petitiana*. Lianas in this community include *Jasminum abyssinicum, Saba comonesis, Capparis tomentosa, Smilax anceps, Rubus apetalus* and *Combretum paniculatum.*

3. *Acacia abyssinica-Millettia ferruginea* community type

This community is spread over altitudes ranging from 1476 to 1792 m.a.s.l. and includes eight plots and 94 species. The dominant tree species in the community are *Croton macrostachyus*, *Albizia gummifera, Buddleja polystachya, Acacia abyssinica,* and *Millettia ferruginea*. Other prominent tree species include *Combertum collinum, Cordia africana, Bridelia micrantha, Croton macrostachyus*, and *Maesa lanceolata*. The shrub layer is dominated by species such as *Maytenus gracilipes, Senna petersiana, Grewia ferruginea, Vernonia auriculifera, Brucea antidysenterica*, and *Nuxia congesta*. The herb layer is characterized by dominant species including *Sonchus bipontini, Tristemma mauritianum, Achyranthus aspra, Bidens macroptera*, and *Oplismenus hirtellus*. In terms of lianas and climbers, the community is characterized by *Paullinia pinnata* and *Cyphostemma adenocaule*. Additionally, this community exhibits seven indicator species with significant indicator values ($p < 0.05$) (Table 3).

4. *Combertum collinum – Bersama abyssinica* community type

This community spread at altitudes between 1630-1711 m.a.s.l. *Pouteria adolfi-friederici* was the only emergent tree species found in this community. *Stereospermum kunthianum, Terminalia macroptera* and *Bridelia micrantha* were dominant tree species next to *Combertum collinum* and *Croton macrostachyus*. Other associated tree species are *Ficus sycomorus, Entada abyssinica, Ximenia americana, Albiza gummifera* and *Flueggea virosa*. The shrub species are dominated by *Clausena anisata, Vepris dainellii, Gardenia ternifolia* and *Acanthus polystachius*. Dominant species of the herb layer are *Haumaniastrum villosum, Helinus mystacinus, Indigofera spicata, Senna occidentalis, Pennisetum thumbergi* and *Crotalaria lachnophora*. *Clematis longicauda* and *Clematis simensis* are the most common climbers in this community. This community has three indicator species, namely, *Combertum collinum, Maytenus obscura, Gnidia glauca* and *Guizotia villosa*, with significant indicator values ($p < 0.05$) (Table 3).

5. *Terminalia macroptera-Piliostigma thonninigii* community type

This community is represented by 10 plots and 98 species, situated within an altitude range of 1501 to 1672 m.a.s.l. The dominant tree species in this community include *Stereospermum kunthianum, Syzygium guineense subsp. afromontanum, Ficus exasperata, Gardenia ternifolia,* and *Flueggea virosa.* Other prominent tree and shrub species found in the community are *Combretum molle, Ficus mucuso, Albizia malacophylla, Pavetta abyssinica, Maytenus obscura, Ximenia americana, Oxythenteria abyssinica,* and *Flacourtia indica*. The herb layer is comprised of species such as *Crotalaria ononoides, Aframomum alboviolaceum, Conyza sumatreosis, Alysicarpus rugosus*, and *Guizotia villosa*. Among the climbers/vines present in this community, *Ampelocissus schimperiana and Cissampelos pareira* are the most common. Additionally, several indicator species with significant indicator values have been identified within this community. The most important of these are *Gardenia ternifolia, Piliostigma thonningii, Syzygium guineense subsp. afromontanum,* and *Terminalia macroptera*, as indicated in Table 4.

**Table 4. Synoptic mean cover value of plant species in the Arjo-Diga forest**

| Species name | Community types (Cluster) | | | | |
|---|---|---|---|---|---|
| | C1 | C 2 | C3 | C4 | C 5 |
| *Maesa lanceolata* | **2.73** | 1.87 | 1.37 | 0.85 | 1.8 |
| *Ehretia cymosa* | **2.32** | 0 | 0 | 0.1 | 0 |
| *Albizia gummifera* | 1.73 | 0.56 | 1.75 | 1.5 | 0 |
| *Cordia africana* | 1.17 | 1.13 | 1.33 | 0.55 | 1.32 |
| *Trichilia dregeana* | 1.16 | **4.33** | 1.08 | 1 | 3 |
| *Flacoutia indica* | 1.03 | **2.18** | 2.94 | 2.6 | 2.8 |

| | | | | | |
|---|---|---|---|---|---|
| *Ficus exasperata* | 0.11 | 2.9 | 1.78 | 2 | 1.98 |
| *Ficus vasta* | 0.54 | **3.18** | 0.65 | 0.25 | 1.45 |
| *Acaccia abyssinica* | 0.64 | 1.6 | **5.31** | 0.1 | 0 |
| *Millettia ferruginea* | 0.09 | 1.34 | **3.35** | 0.15 | 1 |
| *Stereospermum kunthianum* | 0 | 1.3 | 2.33 | 1.75 | 3 |
| *Apodytes dimidiata* | 0 | 1.23 | 2.24 | 1.77 | 0.5 |
| *Ficus thonningii* | 1.09 | 0 | 1.67 | 0.5 | 0.3 |
| *Maytenus gracilipes* | 0.63 | 1.34 | 1.37 | 0.15 | 0 |
| *Combretum collinum* | 0.18 | 2.43 | 0 | **4.54** | 0.6 |
| *Croton macrostachyus* | 0.17 | 1.91 | 0.34 | **2.31** | 3.1 |
| *Bersama abyssinica* | 1.63 | 1.86 | 0.62 | **2.65** | 0.9 |
| *Combretum molle* | 0 | 0.08 | 0 | 2.01 | 1.3 |
| *Bridelia macrantha* | 0.09 | 1.73 | 1.67 | 1.65 | 0.9 |
| *Terminalia macroptera* | 0 | 0.56 | 3.37 | 1.65 | **4.42** |
| *Piliostigma thonningi* | 0.09 | 0 | 2 | 0.45 | **3.34** |
| *Hymenodictylon floribundum* | 0 | 0 | 0.12 | 0.2 | **2.13** |
| *Ximenia americana* | 0 | 0.73 | 1.3 | 2.1 | 1 |
| *Warburgia ugandensis* | 0.09 | 1.17 | 0.65 | 1.54 | 0.4 |

## Richness, diversity, and evenness of plant communities

The diversity analysis, including species richness, diversity, and evenness, was conducted for each plant community type. The results revealed that community 4 exhibited the highest species

richness and Shannon diversity index, followed by communities 2, 1, and 5. Conversely, community 3 displayed the lowest species richness and Shannon diversity among the analyzed communities. In terms of evenness, community 2 demonstrated the highest evenness, followed by communities 5 and 1. On the other hand, community 4 exhibited the lowest Shannon evenness value, indicating a less balanced distribution of species (Table 5).

**Table 5. Diversity analysis for each of the five plant community types in Arjo-Diga Forest.**

| Community types | Species richness | Shannon diversity (H') | Shannon- Evenness(J) |
|---|---|---|---|
| Community 1 | 110 | 4.1 | 0.87 |
| Community 2 | 148 | 4.3 | 0.86 |
| Community 3 | 94 | 3.9 | 0.89 |
| Community 4 | 162 | 4.4 | 0.85 |
| Community 5 | 98 | 4 | 0.88 |

## Similarities between plant communities

According to the results of the plant community similarity analysis, the highest degree of similarity was found between communities 2 and 4. In contrast, the lowest level of similarity was observed between communities 3 and 5 (as indicated in Table 6).

**Table 6. Similarities between five types of plant communities in Arjo Diga forest**

| Community types | CI | C2 | C3 | C4 | C5 |
|---|---|---|---|---|---|

| | | | | | |
|---|---|---|---|---|---|
| C1 | 1 | | | | |
| C2 | 0.57 | 1 | | | |
| C3 | 0.34 | 0.36 | 1 | | |
| C4 | 0.45 | 0.79 | 0.39 | 1 | |
| C5 | 0.35 | 0.42 | 0.22 | 0.68 | 1 |

C1= *Maesa lanceolata-Ehretia cymosa,* C2=*Trichilia dregeana – Flacourtia indica,* C3=*Acacia abyssinica-Millettia ferruginea,* C4= *Combertum collinum – Crotonc macrostachyus,* and C5= *Terminalia macroptera- Piliostigma thonninigii*

## Relationship between community types and environmental variables

.The results of the CCA ordination, presented in Fig 5, depict the relationship between environmental variables, plant community types, and axes. The direction of the arrows in the ordination diagram indicates a positive or negative association between the environmental variables and the axis. The length of the arrow represents the strength of the association and its impact. According to the results of the Adonis test (Table 7), altitude, total nitrogen, and cation exchange capacity exhibited a statistically significant association with community composition and distribution patterns. Altitude, in particular, explained 45.24% of the variance in community distribution, surpassing other environmental variables. The eigenvalues for the first two axes were 0.35 and 0.23, respectively. The cumulative percentage of variance explained by these two axes accounted for 72.31% of the species-environment variation (Table 8).The distribution of species composition within the plant community was found to be strongly associated with environmental factors such as altitude (83%), disturbances (62.83%), total nitrogen (32.21%), phosphorus (19.72%), and cation exchange capacity (13.43%).

**Fig 5. The CCA ordination diagram displays 72 plots, five environmental variables, and five plant communities**

**Table 7. Results of the Adonis functional test of the environmental variables (significant environmental variables are presented by asterisks with their p values).**

| Environmental variables | Df | Sum of Squares | $R^2$ | F | Pr(>F) |
|---|---|---|---|---|---|
| Altitude (m. a. s. l.) | 1 | 1.2540 | 0.0452 | 3.3734 | 0.001*** |
| Slope (°) | 1 | 0.4280 | 0.0154 | 1.1513 | 0.270 |
| Disturbance (%) | 1 | 1.2771 | 0.0460 | 1.1452 | 0.172 |
| SOC (%) | 1 | 0.2935 | 0.0105 | 0.7861 | 0.825 |
| TN (%) | 1 | 0.4328 | 0.0156 | 1.1643 | 0.01* |
| P (ppm) | 1 | 0.2775 | 0.0103 | 0.8138 | 0.79 |
| pH | 1 | 0.3925 | 0.0141 | 1.0560 | 0.409 |
| CEC (cmol $kg^{-1}$) | 1 | 0.5130 | 0.0185 | 1.3799 | 0.05 ** |
| Residuals | 63 | 19.80091 | 0.8449 | | |
| Total | 71 | 27.7161 | 1.0000 | | |

Significance level: 0 '***', 0.001 '**', 0.01 '*', 0.05

The first CCA axis exhibited significant negative correlations with altitude (r = -0.99), TN (r = -0.95), CEC (r = -0.99), and available phosphorus (r = -0.78), while showing a significant positive correlation with disturbances (r = 0.33). On the other hand, the second axis displayed positive associations with CEC and disturbances (Table 8). The CCA ordination of the Arjo-Diga forest study plots revealed the formation of five community types based on species composition. These community types were distinguished by following the arrows of the environmental variables. In the CCA ordination (Fig 5), community 4 was separated from communities 1, 2, 3, and 5 in the first CCA axis by disturbances. Conversely, in the second CCA axis, communities 1, 2, and 3 were separated from communities 4 and 5 by elevation, CEC, total nitrogen, slope, and soil organic carbon.

**Table 8.Monte Carlo permutation test of the relationship between environmental factors and plant community types**

| Environmental variables | CCA1 | CCA2 | $r^2$ | Pr(>r) |
|---|---|---|---|---|
| Altitude | -0.9996 | -0.0262 | 0.8302 | 0.000 *** |
| Disturbance | 0.3374 | 0.9413 | 0.6280 | 0.000*** |
| Total nitrogen | -0.9994 | -0.0332 | 0.3218 | 0.000*** |
| Phosphorus | -0.7867 | -0.6173 | 0.1973 | 0.001** |
| CEC | -0.9998 | 0.0183 | 0.1344 | 0.01 * |
| Eigenvalue | 0.3504 | 0.2346 | | |
| Proportion Explained | 0.2710 | 0.1814 | | |
| Cumulative Proportion | 0.2710 | 0.4524 | | |

Significance Level: 0 ‘***’, 0.001 ‘**’, 0.01 ‘*’, 0.05 ‘.

The results of the post hoc mean comparison between environmental variables and plant community types are presented in Table 9. Community type 1 was found at a higher altitude (2011.8±0.3), while community type 5 was found at a lower (15285±0) altitude. The highest disturbance value (2±0.5) was recorded in community type 5, while the lowest disturbance value (1.4±1.13) was recorded in community 3. The highest CEC value (40.3±6) was recorded in community type 2, while the lowest CEC value (31.2±10.7) was recorded in community type 5. The maximum (4.19) value of soil organic carbon was recorded in community type 2, while the minimum (2.85) value was observed in community type 5. The pH value of the soils varied between them from 4.8±0.5 to 5.1±0.4 (Table 9). Accordingly, community type 4 was found in very strongly acidic soil, while communities 1, 2, 3 and 5 were found in strongly acidic soil.

**Table 9. Mean comparison between environmental variables, and plant community types.**

| Environmental variable | Plant Community Types | | | | |
|---|---|---|---|---|---|
| | C1 | C2 | C3 | C4 | C5 |
| Altitude(m) | 2011.8±0.3$^{d}$ | 1761.4±1.2$^{a}$ | 1917.4±2.1c | 1648.1±.2.5$^{b}$ | 15285±0.5$^{b}$ |
| Slope (%) | 21.8±.3.6$^{bc}$ | 19.8±11bc | 22.7±1.3$^{c}$ | 21.1±5a | 20.3±1$^{a}$ |
| Disturbance | 1.85±1$^{ab}$ | 2±0.6$^{ab}$ | 1.4±1.13$^{a}$ | 1.57±0.7$^{a}$ | 2±0.5ab |
| pH | 5.1±0.4$^{a}$ | 5.08±0.7a | 5.02±0.5$^{a}$ | 4.8±0.5$^{b}$ | 5.1±0.3$^{a}$ |
| CEC | 38.8±7.3$^{c}$ | 40.3±6$^{b}$ | 39.8±8$^{b}$ | 32.9$^{a}$ | 31.2±10.7$^{a}$ |
| SOC | 3.6$^{b}$ | 4.19$^{a}$ | 3.19$^{b}$ | 4.08$^{a}$ | 2.85$^{c}$ |

Values in rows and columns with different letters are significantly different ($p < 0.05$).

## Influence of environmental variables on plant community and plant diversity

Regression analysis revealed significant differences ($p<0.05$) in species richness, diversity, and evenness along environmental gradients (Fig 6). Specifically, the analysis showed that species richness ($R^2$ = 0.07, p< 0.05) (Fig 6A) and Shannon diversity ($R^2$ = 0.05, p < 0.05) (Fig 6B) decreased significantly with increasing altitude. Furthermore, species richness exhibited a negative association with TN ($R^2$=0.04, p<0.05), indicating that higher TN deposition led to a decrease in species richness (Fig 6E). Conversely, the slope demonstrated a significant negative association with Shannon diversity ($R^2$ = 0.0122, p < 0.01) (Fig 6G) and evenness ($R^2$=0.08, p< 0.01) (Fig 6H), suggesting that steeper slopes were associated with lower diversity and evenness. Interestingly, disturbance showed a significant positive association with species richness ($R^2$=0.11, p<0.003) (Fig 6C) and Shannon diversity ($R^2$=0.13, p<0.001) (Fig 6D), indicating that areas with higher disturbance levels exhibited higher species richness and diversity.

**Fig 6. Illustrates the regression analysis conducted to examine the relationships between altitude, slope, TN, and disturbance with species richness (Figs 6A, 6C, 6E, and 6F), Shannon diversity (Figs 6B, 6D, and 6G), and evenness (Fig 6H).**

As depicted in Fig 7, the different types of plant communities were influenced by several environmental factors, including altitude (Fig 7A, slope (Fig 7E, ~~CEC~~CEC (Fig 7D), TN), ~~SOC~~OC (Fig 7G), ~~pH~~pH (Fig 7C), and available phosphorus (Fig 7H). The impact of available phosphorus, ~~S~~OC, and altitude on Community 5 was minimal, although some outliers were observed. However, these factors had a significant influence on Community 1. Community 3 showed a strong association with CEC and TN, while Community 5 exhibited a lesser sensitivity to these variables. Disturbance and pH had a notable effect on Community 5, but their impact was relatively lower on Community 3. Slope demonstrated a fairly consistent pattern in its effects across all communities (Fig 7E).

**Fig 7. Illustrates the influence of environmental variables on different plant community types: Community type 1: *Maesa lanceolata-Ehretia cymosa*, Community type 2: *Trichilia dregeana-Ficus vasta*, Community type 3: *Acacia abyssinica-Millettia ferruginea*, Community type 4: *Combertum collinum-Bersama abyssinica*, and Community type 5: *Terminalia macroptera-Piliostigma thonninigii***

## Pearson correlation between environmental variables, diversity, richness, and evenness

According to the Pearson correlation analysis (Table 9), several variables exhibited significant positive correlations with altitude, including slope, disturbance, CEC, TN, and Av. P. Unexpectedly, slope and species evenness showed a negative association. Negative correlations were found between disturbance and SOC, TN, Av. P, CEC, species richness, and evenness. This suggests that higher disturbance levels were associated with lower values of these variables. Positive correlations were found between SOC, CEC and Av.P, indicating that higher levels of these variables were associated with each other. Additionally, a significant inverse relationship was observed between CEC and Av. P. On the other hand, there was a significant association between Shannon diversity and species richness and evenness. The diversity index and all other environmental variables were negatively correlated with soil pH in indicating that higher pH levels were associated with lower diversity and environmental values.

**Table 10. Pearson correlation coefficients between environmental factors, diversity index in Diga district, western Ethiopia**

| | Alt. | Slope | Dis. | SOC | TN | Av.P | pH | CEC | H' | S | E |
|---|---|---|---|---|---|---|---|---|---|---|---|
| Alt. | | | | | | | | | | | |
| Slope | 0.18* | | | | | | | | | | |
| Dis. | 0.10* | -0.07 | | | | | | | | | |

| | | | | | | | | | | | |
|---|---|---|---|---|---|---|---|---|---|---|---|
| SOC | 0.19* | -0.23 | -0.17* | | | | | | | | |
| TN | 0.46*** | -0.00 | -0.26** | -0.17 | | | | | | | |
| Av.P | 0.25* | -0.04 | -0.28* | 0.07* | -0.11 | | | | | | |
| pH | -0.05 | -0.13 | -0.06 | -0.03 | -0.08 | | | | | | |
| CEC | 0.24*** | 0.16 | -0.27** | -0.16* | -0.00* | 0.01* | 0.00 | | | | |
| H' | 0.01* | -0.08 | 0.12* | 0.12 | 0.08 | 0.04 | -0.1 | 0.09 | | | |
| S | -0.04* | 0.1 | -0.08** | -0.12 | -0.14 | -0.03 | 0.08 | -0.07 | 0.98*** | | |
| E | -0.02* | -0.14* | -0.12* | -0.18 | 0.07 | 0.01 | 0.05 | -0.03 | 0.78*** | -0.74** | |

*. Correlation is significance at the 0.05 level (2-tailed),

**. Correlation is significant at the 0.01 level (2-tailed),

Alt. = altitude, Dis. = Disturbance, Av.P= available phosphorus, SOC= Soil organic carbon, TN=Total nitrogen, CEC=Cation exchange capacity H′=Shannon diversity, S = species richness, and E= evenness.

## Discussion

### Species ~~composition~~ richness of the Arjo-Diga forest

The Arjo-Diga Forest is rich in species richness consisting of economically important species such as *Coffea arabica* and important timber trees namely *Cordia africana*, *Pouteria adolfi friederici*, and *Prunus africana* However, direct comparisons of species richness between forests may not be meaningful due to differences in sample sizes and study objectives [52]. But the Arjo-Diga F~~f~~orest consisted of 234 species which is higher than Masha forest (130 species [53]; Jibat forest, 183 species [54]; Komto forest, 180 species [55]; Belete forest, 157 species [56]; Gelesha forest, 157 species [57], Gendo forest, 168 species [58], ~~and~~ Agama forest, 162 species [59] and Gerba Dima forest,180 species [26]. This could be attributed to its extensive topographic gradient. On the other hand, the total number of species ~~in the study area was~~ is

slightly ~~lower~~ less than the number of species recorded in the Bonga forest (243 species) [60]. This could be attributed to the number of sample plots, geographical location and ~~history of~~ disturbance history, ~~and~~ as well as climatic factors~~,~~ which could illustrate ~~explain~~ the differences in species ~~composition~~ richness between different forest areas [61].

Asteraceae, Fabaceae, Rubiaceae, Euphorbiaceae, Lamiaceae, Acanthaceae, and Poaceae~~In the study area, seven families~~ contributed 38% of the total number of species in the study forest. These dominant families have also been reported as~~Asteraceae, Fabaceae, Rubiaceae, Euphorbiaceae, Lamiaceae, Acanthaceae, and Poaceae~~ ~~were~~ the seven ~~the~~ most species-rich families ~~in the Arjo-Diga Forest, as reported~~ in the flora of Ethiopia and Eritrea [62] and other moist Afromontane forests of southwestern Ethiopia~~. Similarly, other authors~~ [26, 55, 60] ~~also reported that these families were the most species-rich families in the humid Afromontane Forest of southwestern Ethiopia~~. The dominance of these families in the study area is consistent with general dominance in the flora area and tropical forests. ~~was based on~~This is due to their adaptation and colonization of different ecological niches based on their efficient ~~mechanisms of~~ pollination, dispersal, and germination mechanisms. For example, species ~~in the family~~from Asteraceae have an attractive flower color that allows the plant to be easily pollinated by a variety of insects [63].

Among the identified growth forms, h~~H~~erbs account for 36% of the ~~identified~~ species ~~among growth forms~~. The spread of herb could be ~~This, coupled~~attributed to ~~with~~ the ~~occurrence~~ presence of canopy gaps ~~in the canopy~~ caused by anthropogenic disturbance, ~~may~~ which may leads to an increase in the prevalence of herbaceous species ~~result in herbaceous species being more prevalent~~[64]. Additionally, ~~F~~forest disturbances in the form of selective tree clear-cutting

~~favor~~ promote the growth of herbaceous species in the forest ~~undergrowth~~understory. Similarly, study conducted in moist Afromontane forests of Ethiopia by[26, 55, 65] also reported a significant presence of herbs, alongside other growth forms such as trees and shrubs.~~{Dibaba, 2022 #127}~~

~~The study shows that the~~ Arjo-Diga f~~f~~orest is the home to ~~accounts for~~ 20 (8.54%) of the endemic plant species, which is . ~~This species count is~~a higher numbered ~~than~~ compared to the endemic species found in ~~that reported studies in the~~ Harenna Forest, Berhane-Kontir Forest, Wofwasha Forest, Bonga Forest, Yayu Forest, and Agama Forest[27, 66, 67]. This This can be attributed to~~study shows that the large~~ extreme micro-climate condition,including moisture ~~number of endemic species in the study forest is related to various climatic factors prevailing in the area. For example,~~ precipitation, ~~and~~ temperature and spatial heterogeneity, which contribute to ~~significantly to~~ the ~~existence~~presence of endemic species [68]~~ in the area~~. Additionally, the diverse topography of the study forest, characterized by hills, valleys, ridges, and plateaus, plays a crucial role in supporting the existence of endemic species.

~~In addition, the geographic location of the area also leads to the organization of taxa with unique biological characteristics.~~

## Plant community

The hierarchical cluster analysis conducted on the Arjo-Diga f~~f~~orest revealed the presence of five distinct plant communities. These communities were identified by selecting two or more dominant plant species based on their synoptic mean cover values. The dominant plant species were determined by their highest mean cover value within each community. Compared to the other communities, plant communities type ~~I~~ 1 (*Maesa lanceolata-Ehretia cymosa*) and type ~~III~~ 3 (*Acacia abyssinica-Millettia ferruginea*) exhibited the lowest species diversity. This is can be

attributed to anthropogenic disturbances such as cattle grazing, fire, charcoal production, and firewood collection in the plots studied. This finding is consistent with the findings of [69], who ~~found~~ reported that selective logging of economically important woody species and livestock grazing can ~~of~~ ~~reduce~~decrease species richness and ~~species~~ diversity. These community type also include economically important tree species such as *Albizia gummifera* and *Millettia ferruginea*, used as coffee shade; *Ficus thonningi*, used as forage; and *Prunus africana*, used as firewood and agricultural tools. These results support previous research ~~showing~~ indicating that trees in forests ~~help~~ provide various benefits to household ~~members in a variety of ways~~ due to their proximity ~~to forests~~ [70-72]. According to local sources (personal communication with elders), human impacts on the ~~have been observed in the~~ Arjo-Diga forest have been observed for many years, and a significant effort have been made to restore and conserve the forest~~most of today's forest area was formerly used as farmland and human settlements~~. ~~This means that significant conservation measures have been taken to reclaim the forest.~~

The type 2 community (*Trichilia dregeana- Ficus vasta*) showed higher species richness than the other communities, which is attributed to the suitability of the habitat for plant growth and the presence of rivers and streams in the studied plots. Similarly,[67] found that the presence of rivers in sample plots and lower anthropogenic influence may have contributed to the highest species richness and diversity in plant community composition. On the other hand, community type 4 (*Combertum collinum- Bersama abyssinica*), *Apodytes dimidiata*, *Sapium ellipticum*, and *Syzygium guineense ssp. guineense* are distinctive species in the middle canopy of the Arjo-Diga forest. In addition, this community type included the main species that provided traditional medicine, edible fruits, honey bees, and household materials to the local population. For

example, *Schefflera abyssinica* and *Pouteria adolfi-friederici* are dominant emerging tree species for honey bee production, and *Croton macrostachyus, Erythrococca burci, Securridaca longepedunculata,* and *Entada abyssinica* are used for traditional medicine, while *Allophylus abyssinicus, Rhus vulgaris, Syzygium guineense* and *Ximenia americana* are used to provide local people with edible fruit. A similar investigation was reported in Yayu Forest, Sheko Forest, and Bonga Forest by [73, 74].

Community type ~~V~~ 5 (*Terminalia macroptera- Piliostigma thonninigii*) consisted of edible tree species such as *Syzygium guineense subsp. afromontanum, Ximenia americana* and *Gardenia ternifolia* are used as food by many animals and humans. *Stereospermum kunthianum* and *Calpurnia aurea* were used for traditional medicine by the local people in the study area. In addition, economically important species such as *Aframomum corrorima, Piper capense,* and *Dioscorea praehensilis* were present in this community and are used for traditional medicine and food. However, they have been influenced by human activities such as coffee plantations, agricultural expansion, and human settlement. Therefore, this study suggests that protecting the ecosystem ensures the conservation of beneficial plant species on site by using sustainable harvesting methods to take plants from the forest for any purpose.

## Species diversity, richness, and evenness in identified plant communities.

The results of this study indicate slight differences in species richness, diversity, and evenness among the five plant communities. Community types 2 and 4 exhibit relatively high species richness and diversity, while community type 3 has the lowest values. These differences may be attributed to factors such as altitude, human impacts, grazing, and other biotic and abiotic variables among the five community types. Community types 2 and 4 have the highest species diversity and richness because they are situated at moderate altitudes, which create favorable

conditions for rapid resource acquisition and growth of most plants [26, 75]. ~~Conversely~~On the other hand, community type 3, located in close proximity to human settlements primarily between 1476 to 1792 m.a.s.l, experiences lower species richness and diversity. Similar studies conducted elsewhere have also reported reduced plant diversity and richness ~~that~~ in forests near settlements~~ often exhibit~~ ~~ reduced plant diversity and richness~~[76]. Additionally, altitude and climatic factors such as temperature, precipitation, humidity, and seasonal fluctuations play a role in determining species richness and diversity [55, 77]. ~~A study~~Studies conducted in the southwestern Afromontane forest [26] and the Komta moist Afromontane forest [55]~~ -~~ have also observed that anthropogenic disturbances including grazing, agricultural land expansion, and human settlement, can diminish species richness and diversity. Community type 4 demonstrates low plant evenness due to the presence of disturbances and overexploitation of multipurpose species used for timber and other purposes. This leads to the dominance of certain species such as *Trichilia dregeana*, *Pouteria adolfi-friedericii*, and *Vepris dainellii* within community type 4. Conversely, the highest plant evenness is observed in community type 5, indicating recurring coexistence of species across all plots rather than the low dominance of a single species. Hence, the significance of evenness values suggests that in forests, high evenness in specific locations within protected areas may not be as critical as in forests with low evenness.

The similarity/difference between forest communities maintains forest biodiversity and shapes forest composition and structure [78]. Measuring similarity between forest communities is important to assess species changes attributed to species composition through selective tree harvesting [79]. The reason lies in the different abundances of species and the different intensities of disturbance between them. This study was consistent with a study by [80], who

reported that environmental factors such as slope, topography, soil and climate have significant effects on vegetation distribution patterns.

## Relationship between environmental variables and plant community

**Formatted:** Font color: Red

In a given study area, the distribution patterns of plant communities are often influenced by a combination of factors, including altitude, slope, soil nutrients, and disturbances. Specifically, in the study area of the Arjo-Diga forest, the distribution of plant communities is influenced by altitude, total nitrogen (TN), cation exchange capacity (CEC), and disturbances. The results of Canonical Correspondence Analysis (CCA) indicate that altitude is a significant variable in determining the distribution and formation of plant communities in the Arjo-Diga forest. Similar findings have been reported in other studies conducted in Ethiopia, highlighting the impact of elevation variations on the distribution patterns of forest vegetation [26, 81, 82]. By focusing on these factors, conservation efforts can effectively contribute to the preservation and restoration of plant communities in the study area. This, in turn, promotes biodiversity and ensures the overall health of the ecosystem.

Furthermore, our results showed that soil variables such as total nitrogen (TN) and cation exchange capacity (CEC) were significantly associated with plant community formation in the study area. These results suggest that TN and CEC are the most important soil variables in determining plant community distribution. For example, the soil TN explained the significant variation in species composition in community types 1 and 3 (Fig 5). These results could be due to the positive effects of TN on certain strongly competing plants, which forecloses species in competition [83]. This result is very similar to a study by [84], who found a negative trend in species diversity in the plant community with TN in forests in Pennsylvania. Similarly, a study by (2011) showed that plant community composition can be influenced by total nitrogen (TN)

deposition in European acidic grassland ecosystems. The research found that changes in TN deposition resulted in significant changes in plant community composition. Soil cation exchange capacity (CEC) was also found to be a major factor in explaining the differences in species composition between communities 1 and 2 in the Arjo-Diga forest. Similar to our results [85], CEC affects tree species in a plant community by varying soil fertility in the soil. In addition,[86] found that CEC had a positive and significant impact on the species composition of the plant community on the central plateau of Shewa, Ethiopia. The results of this study suggest that the structure of plant communities is significantly influenced by soil chemical and physical properties.

In addition, disturbances also significantly explained differences in species composition between plant communities. In this study, we found that community 5 was the most disturbed plant community due to its proximity to adjacent agricultural activities and human settlements. The study showed that the species richness of communities 1, 2, and 4 increased in the studied plot with a moderate level of disturbance. These results are consistent with the "intermediate disturbance hypothesis" [87], which suggests that species richness is maximized when ecological disturbances occur at intermediate frequency. This finding is consistent with [88], who reported that plant communities at moderately disturbed sites have high species composition and diversity.

## Relationship between environmental variables and diversity metrics such as species diversity, species richness and evenness

In ecological studies, it is crucial to measure diversity metrics such as species diversity, richness, and evenness to gain insight into community dynamics and their response to biotic and abiotic factors [47, 89].

In this study, topographic factors and disturbances are important determinants of species diversity, species richness, and evenness distribution [90]. In our study, altitude had a significant impact on the species richness and diversity of the Arjo-Diga forest. Species richness and species diversity showed a significant relationship with altitude [8, 9], therefore, species richness and species diversity decrease linearly with increasing altitude. This is consistent with other studies in which species richness and species diversity decreased monotonically along elevation gradients[91]. This shows the strong dependence of species richness and diversity on altitude. Our results are consistent with several previous studies in Ethiopia [84, 92, 93]. At high altitudes, the combination of extreme environmental conditions and selective filters leads to a reduction in species richness and species diversity. Only the plant species best adapted to these conditions can survive and reproduce.

The present study found a significant relationship between slope gradient and species diversity and evenness. We observed that the Shannon–Wiener index (H) and evenness began to decrease at the slope gradient level (30-50°), which was due to the deterioration of soil nutrient spatial conditions and root distribution with increasing slope gradient (see Fig. 6). Therefore, slope is considered to play a crucial role in determining species diversity and evenness, as argued by various authors, such as [94, 95]. Other studies, such as [95, 96], have shown that slope gradients have a significant impact on species diversity and evenness by altering important ecological factors such as humidity, heat, light intensity and soil conditions. However, high species

diversity and evenness have been observed on steep slopes in Semen Mountain National Park [80], as there is less human disturbance on steep slopes.

Disturbance plays a crucial role in shaping species diversity, richness, and evenness in the current study. The research findings indicate that species richness and diversity exhibit an increasing trend with rising levels of human disturbance, reaching a peak at a certain threshold. However, beyond this threshold, species richness and diversity start to decline (Fig 6). This pattern is attributed, in part, to the emergence of disturbance-tolerant and fast-growing ruderal species during moderate disturbances. These results are consistent with findings from other studies [97-99], which consistently demonstrate that species richness and biodiversity are generally higher under moderate disturbance levels but tend to decrease under severe disturbance conditions.

In addition, in the studied forest coffee plantation and management practices, a reduction in tree species richness and diversity were observed. This is because people tend to select smaller trees for logging, while larger trees are needed for shading coffee under the canopy. Similar studies by[55, 97, 98] in southwestern Afromontane forests have reported that the selective removal of economically important tree species and grazing by livestock could contribute to low species richness and diversity in a given forest. Additionally, significant edge effects were observed, with various forms of human disturbance being more noticeable closer to agricultural edges in the study forest. This was expected, as people in the study area heavily rely on the forest for ecosystem services such as wild coffee and spice plantations like *Piper capense* and *Aframomum corrorima*. These findings align with [99, 100], who reported that people living near forests are largely dependent on them for provisioning ecosystem services such as wild coffee, spices, firewood, and honey.

**Commented [H9]:** This on the revised paragraph based on reviewer comment

## Correlation between environmental variables

Pearson correlation analysis showed that altitude is positively correlated with soil organic carbon (SOC), with SOC increasing with altitude. The increase in SOC with altitude is likely due to a combination of factors, including temperature, precipitation, topography, and vegetation cover, all of which may contribute to organic matter accumulation [105]. Our results are consistent with results [106, 107] reporting that the SOC in the Himalayan forest is positively correlated with altitude. However, a study by [108] found that the SOC in Nepalease National Park forest decreased with increasing altitude. This could be due to lower decomposition associated with organic carbon production and long-term accumulation. Therefore, this study confirms that altitude is an effective indicator of forest soil organic carbon content.

Moreover, a positive correlation was observed between Cation Exchange Capacity (CEC) and altitude. This association could be explained by the higher vegetation density at higher altitudes, which leads to increased organic matter production and, consequently, higher CEC[109]. Similarly, a study on tea plantations in Indonesia reported that the addition of organic matter to the soil can improve soil CEC[110]. However, some authors[111] have reported a negative correlation between CEC and SOC, as decreased decomposability of Soil Organic Matter (SOM) leads to a decrease in CEC.SOC and total nitrogen (TN) exhibit a positive correlation due to their common origin from organic matter. Nitrogen is released as SOM decomposes, resulting in higher TN content in soils with high SOC. These results align with the findings of [112].Furthermore, there is a negative correlation between soil pH and SOC, as soil pH plays a crucial role in regulating the diversity of microbial communities that affect the rate of SOC degradation[110].By understanding the interrelationships among these soil properties,

appropriate soil management practices can be implemented to enhance soil fertility and support diverse plant communities.

# Conclusion

The aim of the study was to investigate how environmental factors, including elevation, slope, soil nutrients, and disturbance, influence species richness, diversity, and plant communities in the Arjo-Diga forest in western Ethiopia. The results revealed that the forest exhibits high biodiversity, including endemic species, highlighting the urgent need for immediate protection to prevent irreversible loss. Plant community diversity exhibited variations and was associated with environmental factors such as soil nutrients, disturbance, and topography. Notably, the *Acacia abyssinica-Millettia ferruginea* community displayed the lowest species richness and diversity indices, emphasizing the importance of conservation efforts in this community. Altitude was identified as a significant factor impacting species diversity and distribution, with fewer species occurring at lower elevations due to anthropogenic disturbances such as fuelwood extraction, selective logging, and the harvesting of non-timber forest products. Soil nutrients and anthropogenic disturbances also influenced the species composition and diversity of plant communities. The study recommended further research to investigate the effects of climate change on plant community formation and species distribution, the impact of slope aspect on

**Formatted:** Font: Italic

floristic composition and diversity, and the consequences of land-use change on vegetation cover.

## Acknowledgments

The authors would like to express their sincere gratitude to the Diga District Agriculture and Rural Development Office for providing valuable information that significantly contributed to the successful completion of this study. They would also like to extend their thanks to the staff of the Ethiopian National Herbarium at Addis Ababa University for their invaluable assistance in identifying the specimens. Additionally, the authors wholeheartedly appreciate the support and cooperation received from the local authorities and community of the Diga district, as their involvement greatly facilitated the accomplishment of this study.

# References


1. Dakhil, M.A., et al., *Richness patterns of endemic and threatened conifers in south-west China: topographic-soil fertility explanation.* Environmental Research Letters, 2021. **16**(3): p. 034017.
2. Gebrehiwot, K., et al., *Elevational changes in vascular plants richness, diversity, and distribution pattern in Abune Yosef mountain range, Northern Ethiopia.* Plant diversity, 2019. **41**(4): p. 220-228.
3. Naud, L., et al., *Altitude effects on spatial components of vascular plant diversity in a subarctic mountain tundra.* Ecology and evolution, 2019. **9**(8): p. 4783-4795.
4. Costanza, J.K., et al., *Classifying forest inventory data into species-based forest community types at broad extents: exploring tradeoffs among supervised and unsupervised approaches.* Forest Ecosystems, 2018. **5**(1).
5. Nepali, B.R., J. Skartveit, and C.B. Baniya, *Impacts of slope aspects on altitudinal species richness and species composition of Narapani-Masina landscape, Arghakhanchi, West Nepal.* Journal of Asia-Pacific Biodiversity, 2021. **14**(3): p. 415-424.
6. Liu, Y., et al., *Effects of contemporary environment and Quaternary climate change on drylands plant diversity differ between growth forms.* Ecography, 2019. **42**(2): p. 334-345.
7. Pandey, K., Y. Adhikari, and M. Weber, *Structure, composition and diversity of forest along the altitudinal gradient in the Himalayas, Nepal.* Appl Ecol Environ Res, 2016. **14**(2): p. 235-251.
8. Gairola, S., et al., *Species richness and diversity along an altitudinal gradient in moist temperate forest of Garhwal Himalaya.* Am J Sci, 2009. **5**: p. 119-128.
9. Trigas, P., et al. *Elevational gradient of vascular plant species richness and endemism in Crete*. in *Poster 248 at the Fifth Biennial Conference of the International Biogeography Society, Irakleion, Crete, Greece*. 2011.
10. Ahmad, M., et al., *Patterns of plant communities along vertical gradient in Dhauladhar Mountains in Lesser Himalayas in North-Western India.* Science of The Total Environment, 2020. **716**: p. 136919.
11. Bennie, J., et al., *Influence of slope and aspect on long-term vegetation change in British chalk grasslands.* Journal of ecology, 2006. **94**(2): p. 355-368.
12. Fadrique, B. and J. Homeier, *Elevation and topography influence community structure, biomass and host tree interactions of lianas in tropical montane forests of southern Ecuador.* Journal of vegetation science, 2016. **27**(5): p. 958-968.
13. Waring, B.G., J.M. Becknell, and J.S. Powers, *Nitrogen, phosphorus, and cation use efficiency in stands of regenerating tropical dry forest.* Oecologia, 2015. **178**(3): p. 887-897.
14. Fisher, J.B., et al., *Nutrient limitation in rainforests and cloud forests along a 3,000-m elevation gradient in the Peruvian Andes.* Oecologia, 2013. **172**(3): p. 889-902.
15. Stevens, C.J., et al., *Impact of nitrogen deposition on the species richness of grasslands.* Science, 2004. **303**(5665): p. 1876-1879.
16. Huston, M., *Soil nutrients and tree species richness in Costa Rican forests.* Journal of Biogeography, 1980: p. 147-157.
17. Nadeau, M.B. and T.P. Sullivan, *Relationships between plant biodiversity and soil fertility in a mature tropical forest, Costa Rica.* International Journal of Forestry Research, 2015. **2015**.
18. Zhu, Y., et al., *The effects of crop tree thinning intensity on the ability of dominant tree species to sequester carbon in a temperate deciduous mixed forest, northeastern China.* Forest Ecology and Management, 2022. **505**: p. 119893.

19. Shrestha, K.B., et al., *Effect of anthropogenic disturbance on plant species diversity in oak forests in Nepal, Central Himalaya.* International Journal of Biodiversity Science, Ecosystem Services & Management, 2013. **9**(1): p. 21-29.
20. Zhang, J., et al., *Long-term grazing effects on vegetation characteristics and soil properties in a semiarid grassland, northern China.* Environmental Monitoring and Assessment, 2017. **189**: p. 1-13.
21. Willig, M. and S. Presley, *Biodiversity and disturbance.* The encyclopedia of the Anthropocene, 2018. **3**: p. 45-51.
22. Collins, S.L., S.M. Glenn, and D.J. Gibson, *Experimental analysis of intermediate disturbance and initial floristic composition: decoupling cause and effect.* Ecology, 1995. **76**(2): p. 486-492.
23. Volis, S., *Conservation-oriented restoration–a two for one method to restore both threatened species and their habitats.* Plant Diversity, 2019. **41**(2): p. 50-58.
24. Gebretsadik, T., *Causes for biodiversity loss in Ethiopia: A review from conservation perspective.* South Agricultural Research Institute (SARI), Hawassa Agricultural Research center, 2016. **6**(11).
25. Kebede, M., et al., *Vegetation structural characteristics and topographic factors in the remnant moist Afromontane forest of Wondo Genet, south central Ethiopia.* Journal of forestry research, 2013. **24**: p. 419-430.
26. Dibaba, A., T. Soromessa, and B. Warkineh, *Plant community analysis along environmental gradients in moist afromontane forest of Gerba Dima, South-western Ethiopia.* BMC Ecology and Evolution, 2022. **22**(1): p. 12.
27. Teketay, D. and T. Bekele, *Floristic composition of Wof-Washa natural forest, Central Ethiopia: Implications for the conservation of biodiversity.* Feddes Repertorium, 1995. **106**(1-5): p. 127-147.
28. Siraj, M., et al., *Floristic composition and plant community types in Maze National Park, southwest Ethiopia.* Applied Ecology and Environmental Research, 2017. **15**(1): p. 245-262.
29. Jaleta, G. and H. Jebssa *The impact of large scale agriculture on forest and wildlife in Diga Woreda, Western Ethiopia.* Asian Journal of Agriculture, 2017. **1**(02): p. 100-113.
30. Jaleta, O.G. and H. Jebessa *The impact of large scale agriculture on forest and wildlife in Diga Woreda, Western Ethiopia.* Asian Journal of Agriculture, 2017. **1**(02): p. 100-113.
31. Mohr, P.A., *The geology of Ethiopia*. 1971: Haile Selassie I University Press.
32. Scholte, P., *Atlas of the Potential Vegetation of Ethiopia. Ib Friis, Sebsebe Demissew & Paulo van Breugel. Copenhagen: The Royal Danish Academy of Sciences and Letters in Biologiske Skrifter58. 2010. 307 pp. including 29 colour map plates (1: 2 000 000). ISSN 0366 3612, ISBN 97887 7304 347 9. 400 DKK (approx.£ 46) paperback.* Edinburgh Journal of Botany, 2012. **69**(1): p. 207-209.
33. Mueller-Dombois, D. and H. Ellenberg, *Aims and methods of vegetation ecology*. 1974: Wiley.
34. Van der Maarel, E., *Transformation of cover-abundance values in phytosociology and its effects on community similarity.* Vegetatio, 1979. **39**(2): p. 97-114.
35. Nelson, D.a. and L.E. Sommers, *Total carbon, organic carbon, and organic matter.* Methods of soil analysis: Part 2 chemical and microbiological properties, 1983. **9**: p. 539-579.
36. Skjemstad, J. and R. Reeve, *The determination of nitrogen in soils by rapid high-temperature Kjeldahl digestion and autoanalysis.* Communications in Soil Science and Plant Analysis, 1976. **7**(3): p. 229-239.
37. Ryti, R., *On the determination of soil pH.* Agricultural and Food Science, 1965. **37**(1): p. 51-60.
38. Gutierrez Boem, F.H., G. Rubio, and D. Barbero, *Soil phosphorus extracted by Bray 1 and Mehlich 3 soil tests as affected by the soil/solution ratio in Mollisols.* Communications in soil science and plant analysis, 2011. **42**(2): p. 220-230.

39. Chapman, H., *Cation-exchange capacity.* Methods of soil analysis: Part 2 Chemical and microbiological properties, 1965. **9**: p. 891-901.
40. Anderson, E.W. and W.F. Currier, *Evaluating zones of utilization.* Rangeland Ecology & Management/Journal of Range Management Archives, 1973. **26**(2): p. 87-91.
41. Yeshitela, K. and T. Bekele, *Plant community analysis and ecology of Afromontane and transitional rainforest vegetation of southwestern Ethiopia.* SINET: Ethiopian Journal of Science, 2002. **25**(2): p. 155-175.
42. Legendre, P. and L. Legendre, *Numerical ecology*. 2012: Elsevier.
43. Voigt, W., *PC-ORD Reviews.* ESA Bulletin. **92**(3): p. 298-301.
44. Team, R.C., *R: A language and environment for statistical computing. R Foundation for Statistical Computing, Vienna, Austria.* http://www. R-project. org/, 2016.
45. Mbuga, F. and C. Tortora, *Spectral clustering of mixed-type data.* Stats, 2021. **5**(1): p. 1-11.
46. Maechler, M., et al., *Cluster: cluster analysis basics and extensions.* 2012.
47. Magurran, A.E., *Measuring biological diversity.* Current Biology, 2021. **31**(19): p. R1174-R1177.
48. Sorensen, T.A., *A method of establishing groups of equal amplitude in plant sociology based on similarity of species content and its application to analyses of the vegetation on Danish commons.* Biol. Skar., 1948. **5**: p. 1-34.
49. Kent, M., *Vegetation description and data analysis: a practical approach*. 2011: John Wiley & Sons.
50. Chao, A., C.-H. Chiu, and L. Jost, *Unifying species diversity, phylogenetic diversity, functional diversity, and related similarity and differentiation measures through Hill numbers.* Annual review of ecology, evolution, and systematics, 2014. **45**: p. 297-324.
51. Field, A., *Discovering statistics using IBM SPSS statistics*. 2013: sage.
52. Gole, T.W., *Vegetation of the Yayu forest in SW Ethiopia: impacts of human use and implicatiions for in situ conservation of wild Coffea arabica L. Populations*. Vol. 10. 2003: Cuvillier Verlag.
53. Assefa, A., S. Demissew, and Z. Woldu, *Floristic composition, structure and regeneration status of M asha forest, south-west E thiopia.* African Journal of Ecology, 2014. **52**(2): p. 151-162.
54. Burju, T., K. Hundera, and E. Kelbessa, *Floristic composition and structural analysis of Jibat humid afromontane forest, west Shewa zone, Oromia national regional state, Ethiopia.* Ethiopian Journal of Education and Sciences, 2013. **8**(2): p. 11-34.
55. Gurmessa, F., T. Soromessa, and E. Kelbessa, *Floristic Composition and Community Analysis of Komto Afromontane Moist Forest, East Wollega Zone, West Ethiopia.* Science, Technology and Arts Research Journal, 2013. **2**(2): p. 58-69.
56. Gebrehiwot, K. and K. Hundera, *Species composition, plant community structure and natural regeneration status of Belete moist evergreen montane forest, Oromia regional state, Southwestern Ethiopia.* Momona Ethiopian Journal of Science, 2014. **6**(1): p. 97-101.
57. Bilew, A., H. Kitessa, and A. Balcha, *Floristic composition and structural analysis of Gelesha forest, Gambella regional State, Southwest Ethiopia.* Journal of Ecology and the Natural Environment, 2015. **7**(7): p. 218-227.
58. Gemechu, T., E. Kelbessa, and T. Soromessa, *Floristic Composition and Community analysis of Gendo Moist Montane Forest of East Wellega, Western Ethiopia.* Journal of Natural Sciences Research, 2015. **5**: p. 15.
59. Admassu, A., et al., *Floristic composition and plant community types of Agama Forest, an â€ œAfromontane Forestâ€ in Southwest Ethiopia.* Journal of Ecology and the Natural Environment, 2016. **8**(5): p. 55-69.

60. Kelbessa, E. and T. Soromessa, *Interfaces of regeneration, structure, diversity and uses of some plant species in Bonga Forest: A reservoir for wild coffee gene pool.* SINET: Ethiopian Journal of Science, 2008. **31**(2): p. 121-134.
61. Song, X., et al., *Different environmental factors drive tree species diversity along elevation gradients in three climatic zones in Yunnan, southern China.* Plant Diversity, 2021. **43**(6): p. 433-443.
62. Kelbessa, E. and S. Demissew, *Diversity of vascular plant taxa of the flora of Ethiopia and Eritrea.* Ethiopian Journal of Biological Sciences, 2014. **13**(Supp.)): p. 37-45.
63. Gupta, R.K., *Features of Afro-Alpine Plant Ecology.(Acta Phytogeographica Succica 49)*. 1965, JSTOR.
64. Garg, S., R.K. Joshi, and S.C. Garkoti, *Effect of tree canopy on species composition, diversity and biomass of herbaceous vegetation and soil characteristics in semi-arid forests of the Aravalli hills.* 2020.
65. Addi, A., et al., *Floristic composition and plant community types of Agama Forest, an Afromontane Forest in Southwest Ethiopia.* Journal of Ecology and the Natural Environment, 2016. **8**(5): p. 55-69.
66. Wakjira, F.S., *Biodiversity and ecology of Afromontane rainforests with wild Coffea arabica L. populations in Ethiopia*. 2006: Cuvillier Verlag.
67. Addi, A., T. Soromessa, and T. Bareke, *Floristic composition and vegetation community analysis of Gesha and Sayilem Forest in Kaffa Zone south western Ethiopia.* Biodiversitas Journal of Biological Diversity, 2020. **21**(7).
68. Maharjan, S.K., et al., *Temperature and soils predict the distribution of plant species along the Himalayan elevational gradient.* Journal of Tropical Ecology, 2022. **38**(2): p. 58-70.
69. Mulugeta, Y., T. Bekele, and E. Kelbessa, *Floristic Composition, Species Diversity and Vegetation Structure of Gera Moist Montane Forest, Jimma Zone of Oromia National Regional State, Southwest Ethiopia.* Ethiopian Journal of Biological Sciences, 2015. **14**(1): p. 45-68.
70. Muleta, D., et al., *Socioeconomic benefits of shade trees in coffee production systems in Bonga and Yayuhurumu districts, southwestern Ethiopia: farmers' perceptions.* Ethiopian Journal of Education and Sciences, 2011. **7**(1): p. 39-55.
71. Hundera, K., *Shade tree selection and management practices by farmers in traditional coffee production systems in Jimma Zone, Southwest Ethiopia.* Ethiopian Journal of education and sciences, 2016. **11**(2): p. 91-105.
72. Gemechu, H.W., D. Lemessa, and D.B. Jiru, *A comparative analysis of indigenous and exotic tree species management practices in agricultural landscapes of Southwest Ethiopia.* Trees, Forests and People, 2021. **4**: p. 100059.
73. Senbeta, F., et al., *Plant diversity, vegetation structure and relationship between plant communities and environmental variables in the Afromontane Forests of Ethiopia.* SINET: Ethiopian Journal of Science, 2014. **37**(2): p. 113-130.
74. Alebachew, G.W., T.A. Abejew, and Z. Tesema, *Honeybee Colony Management Practices and Identification of Honeybee Floras in Eastern Amhara, Ethiopia.* Journal of Apiculture, 2022. **2**: p. 151-161.
75. Jamil, A., et al., *Impact of human settlements on diversity of range vegetation.* Sustainability, 2022. **14**(1): p. 519.
76. Somashekar, R., M.B. Raj, and B. Nagaraja. *Impact of human settlement on forest structure and composition in Kudremukh national park, Western Ghats, south India*. in *XII World Forestry Congress, Quebec City*. 2003.

77. Yao, Z., et al., *Precipitation and temperature regulate species diversity, plant coverage and aboveground biomass through opposing mechanisms in large-scale grasslands.* Frontiers in Plant Science, 2022. **13**: p. 999636.
78. Socolar, J.B., et al., *How should beta-diversity inform biodiversity conservation?* Trends in ecology & evolution, 2016. **31**(1): p. 67-80.
79. Hao, M., et al., *Assessing biological dissimilarities between five forest communities.* Forest Ecosystems, 2019. **6**(1): p. 1-8.
80. Wondie, M., et al., *Relationship between topographic variables and land cover in the Simen Mountains National Park, a World Heritage Site in northern Ethiopia.* International Journal of Remote Sensing Applications, 2012. **2**(2): p. 36-43.
81. Kebede, M., et al., *Soil seed bank and seedlings bank composition and diversity of Wondo Genet moist Afromontane forest South Central Ethiopia.* International Journal of Botany, 2012. **8**(4): p. 170-180.
82. Teshome, M., Z. Asfaw, and M. Mohammed, *Diversity Influences Tree Aboveground Carbon Stocks Through Functional Diversity and Functional Dominance in the Dry Evergreen Afromontane Forest of Hararghe Highland, Southeast Ethiopia.* 2020.
83. Djordjević, V., et al., *Patterns of distribution, abundance and composition of forest terrestrial orchids.* Biodiversity and Conservation, 2020. **29**(14): p. 4111-4134.
84. Rawal, R.S., et al., *Plant species diversity and rarity patterns along altitude range covering treeline ecotone in Uttarakhand: conservation implications.* Trop. Ecol, 2018. **59**(2): p. 225-239.
85. Zheng, X., X. Wei, and S. Zhang, *Tree species diversity and identity effects on soil properties in the Huoditang area of the Qinling Mountains, China.* Ecosphere, 2017. **8**(3): p. e01732.
86. Bekele, T., *Phytosociology and ecology of a humid Afromontane forest on the central plateau of Ethiopia.* Journal of Vegetation Science, 1994. **5**(1): p. 87-98.
87. Collins, S.L. and S.M. Glenn, *Intermediate disturbance and its relationship to within-and between-patch dynamics.* New Zealand Journal of Ecology, 1997: p. 103-110.
88. Fakhry, A.M. and G.S. Aljedaani, *Impact of disturbance on species diversity and composition of Cyperus conglomeratus plant community in southern Jeddah, Saudi Arabia.* Journal of King Saud University-Science, 2020. **32**(1): p. 600-605.
89. Maestre, F.T., et al., *Plant species richness and ecosystem multifunctionality in global drylands.* Science, 2012. **335**(6065): p. 214-218.
90. Sharma, P., et al., *Floristic diversity and distribution pattern of plant communities along altitudinal gradient in Sangla Valley, Northwest Himalaya.* The Scientific World Journal, 2014. **2014**.
91. Grytnes, J.A. and O.R. Vetaas, *Species richness and altitude: a comparison between null models and interpolated plant species richness along the Himalayan altitudinal gradient, Nepal.* The American Naturalist, 2002. **159**(3): p. 294-304.
92. McCain, C.M. and J.-A. Grytnes, *Elevational gradients in species richness.* eLS, 2010.
93. Meragiaw, M., et al., *Floristic composition and structure of the Kibate Forest along environmental gradients in Wonchi, Southwestern Ethiopia.* Journal of Forestry Research, 2021: p. 1-14.
94. Boll, T., et al., *Spatial distribution and environmental preferences of the piassaba palm Aphandra natalia (Arecaceae) along the Pastaza and Urituyacu rivers in Peru.* Forest Ecology and Management, 2005. **213**(1-3): p. 175-183.
95. Zeng, X.H., et al., *Slope aspect and slope position have effects on plant diversity and spatial distribution in the hilly region of Mount Taihang, North China.* J. Food Agric. Environ., 2014. **12**(1): p. 391-397.

96. ZHANG, C.-s., et al., *Effects of topographic factors on the plant species richness and distribution pattern of alpine meadow in source region of Lancang River, Southwest China.* Chinese Journal of Ecology, 2012. **31**(11): p. 2767.
97. Senbeta, F. and M. Denich, *Effects of wild coffee management on species diversity in the Afromontane rainforests of Ethiopia.* Forest Ecology and Management, 2006. **232**(1-3): p. 68-74.
98. Shumi, G., et al., *Woody plant use and management in relation to property rights: a social-ecological case study from southwestern Ethiopia.* Ecosystems and People, 2019. **15**(1): p. 303-316.
99. Hundera, K., et al., *Effects of coffee management intensity on composition, structure, and regeneration status of Ethiopian moist evergreen afromontane forests.* Environmental management, 2013. **51**: p. 801-809.
100. Shumi, G., et al., *Conservation value of moist evergreen Afromontane forest sites with different management and history in southwestern Ethiopia.* Biological conservation, 2019. **232**: p. 117-126.
101. Beche, D., et al., *Spatial variation in human disturbances and their effects on forest structure and biodiversity across an Afromontane forest.* Landscape Ecology, 2022. **37**(2): p. 493-510.
102. Huston, M.A., *Disturbance, productivity, and species diversity: empiricism vs. logic in ecological theory.* Ecology, 2014. **95**(9): p. 2382-2396.
103. Li, W., et al., *Effects of different grazing disturbances on the plant diversity and ecological functions of alpine grassland ecosystem on the qinghai-Tibetan plateau.* Frontiers in Plant Science, 2021: p. 2909.
104. Yirga, F., et al., *Impact of altitude and anthropogenic disturbance on plant species composition, diversity, and structure at the Wof-Washa highlands of Ethiopia.* Heliyon, 2019. **5**(8): p. e02284.
105. Schultz, H., *Soil, vine, climate change; the challenge of predicting soil carbon changes and greenhouse gas emissions in vineyards and is the 4 per 1000 goal realistic? This article is published in cooperation with Terclim 2022 (XIVth International Terroir Congress and 2nd ClimWine Symposium), 3-8 July 2022, Bordeaux, France.* OENO One, 2022. **56**(2): p. 251-263.
106. Devi, S.B. and S.S.S.S. Sherpa, *Soil carbon and nitrogen stocks along the altitudinal gradient of the Darjeeling Himalayas, India.* Environmental monitoring and assessment, 2019. **191**: p. 1-18.
107. Kumar, A., et al., *Forest soil nutrient stocks along altitudinal range of Uttarakhand Himalayas: An aid to Nature Based Climate Solutions.* Catena, 2021. **207**: p. 105667.
108. Shapkota, J. and G. Kafle, *Variation in soil organic carbon under different forest types in Shivapuri Nagarjun National Park, Nepal.* Scientifica, 2021. **2021**.
109. Kilambo, D.L., et al., *Effect of soils properties on the quality of compact arabica hybrids in Tanzania.* American Journal of Research Communication, 2015. **3**(1): p. 15-19.
110. Gebrehiwot, K., et al., *Soil organic carbon stock in Abune Yosef afroalpine and sub-afroalpine vegetation, northern Ethiopia.* Ecological Processes, 2018. **7**(1): p. 1-9.
111. Zhang, X.-N., et al., *Influence of edaphic factors on plant distribution and diversity in the arid area of Xinjiang, Northwest China.* Arid land research and Management, 2018. **32**(1): p. 38-56.
112. Fang, K., et al., *Decreased soil cation exchange capacity across northern China's grasslands over the last three decades.* Journal of Geophysical Research: Biogeosciences, 2017. **122**(11): p. 3088-3097.

S1 Appendix. Floristic list of species in the Arjo-Diga forest. (DOCX)

Manuscript ID PONE-D-23-07720

Subject: Resubmitting the Revised Manuscript

Dear Reviewer 2,

Thank you for providing us with the opportunity to submit a revised version of our manuscript titled "Topography Factors and Soil Variables drive Plant Community Distribution Pattern and Species Richness in Arjo Diga Forest, Western Ethiopia" for publication in the PLOS ONE journal. We would like to express our gratitude for the time and effort you and the reviewers dedicated to reviewing our manuscript. We greatly appreciate the insightful comments and valuable improvements suggested.

We have carefully considered the comment regarding including a map showing the location of Ethiopia in Africa. We agree that such a map would enhance the clarity and context of our study. Therefore, we have now included a map in the revised manuscript to provide readers with a visual representation of the study area. Thank you once again for your valuable feedback. We believe that the revisions have significantly strengthened the manuscript, and we hope that it now meets the standards for publication in PLOS ONE.

Sincerely

Tariku Berihun (on behalf of all coauthors)

Lecturer at Dilla University

Ph.D. fellow in Plant Biology and Biodiversity Management, Addis Ababa University

Email: berihun.tariku@yahoo.com

Manuscript ID PONE-D-23-07720

**Response to Reviewer 3**

**Subject: Resubmitting the revised Manuscript**

Thank you for giving us the opportunity to submit a revised draft of the manuscript "**Topography Factors and Soil Variables Control Plant Community Distribution Pattern and Species Richness in Arjo Diga Forest, Western Ethiopia**" for publication in PLOS ONE journal. We sincerely appreciate the time and effort you and the reviewers have dedicated to providing feedback on our manuscript. We are grateful for the insightful comments and valuable improvements made to our article. We have accepted the majority of the editor's suggestions and have also corrected the grammar errors, tables and figures formatting problem according to journal

Sincerely

Tariku Berihun (on behalf of all coauthors)

Lecturer at Dilla University

Ph.D. fellow in Plant Biology and Biodiversity Management, Addis Ababa University

Email: berihun.tariku@yahoo.com

**Comments from Reviewer 3**

Abstract

**Comment 1**: L17-18, the plant family information is in between sentences, which discusses plant communities. The author can start with plant families, followed by another sentence about plant communities or vice versa

**Response 1**: Thank you for your suggestion. We agree with your point and have revised the manuscript accordingly. In the revised version, we have started with a sentence discussing plant families, followed by a sentence addressing plant communities. This reorganization improves the flow and clarity of the information.

Introduction

**Comment 2:** The full form of TN and CEC needs to be included in the introduction.

**Response 2:** Thank you. We wrote the full form of TN, and CEC based on a reviewer's in the introductory section

**Comment 3:** L72-77 It would be nice if author could provide references for the following sentences

**Response** 3: Thank you. We provide and cite references based on a reviewer's comment

## Materials and Methods

**Comments 4:** L113 Here, the author may introduce meters above sea level abbreviation as (m.a.s.l.), which can be used for the rest of the sections and throughout this section; authors can cite the references directly instead of using according to

**Response 4:** Thank you for this valuable suggestion. We have incorporated the suggested abbreviation "m.a.s.l." (meters above sea level) in the manuscript. This abbreviation was used consistently throughout the rest of the sections to provide a concise and standardized representation of elevation measurements.

**Comment 5:** Throughout this section, authors can cite the references directly instead of using according to …

**Response 5**: Thank you for bringing this to our attention. We have made revisions to this section by directly citing the relevant references instead of using the phrase "according to." The direct citation of appropriate sources aims to enhance the clarity and accuracy of the information presented in the manuscript.

**Comment 6**: Line 147-155.ethical statement.

Although it is an important point, I am unsure if the ethical statement must be a part of the methodology section. If it is only about the permits/permission the authors required for the sample collection, this will fit well in the acknowledgements.

**Response 6**: Thank you. We carefully considered the placement of the ethical statement in the manuscript. After consideration, we agree that it would be more appropriate to include this information in the Acknowledgments section rather than the Methodology section, as the ethical statement relates to the permits/permissions for authors to collect samples from the study area. On this basis, we agreed to move the ethical statement to the Acknowledgments section based on the reviewer's comment, as the idea is to reflect the permits/permissions that the authors have for the collection of plant and soil samples from the study area.

## Methods of data collection

**Comment 7**: L162: Use abbreviations (m.a.s.l.)

**Response 7**: Thank you very much. As suggested, the abbreviation m.a.s.l. were used throughout our manuscript.

Comment 8: L165: Does it mean that out of 72 plots, a single plot size was 30 m x 30 m (900 m2) and inside each such plot authors also laid 5 m x 5 m (25 m2) and five 2 m x 2 m (4 m2) to record other life forms such as shrubs and herbs? If it is so, then it needs to be rewritten.

Also, unlike herbs, how many 5 m x 5 m (25 m2) were laid in each plot needs to be clearly mentioned

**Response 8**: Thank you for your comment and clarification. We apologize for any confusion caused by the sentence. To provide a clear explanation, we have revised the sentence as follows: yes, out of the 72 plots, each plot had a size of 30 m x 30 m (900 m2). Additionally, within each large plot, we laid five smaller plots, including 5 m x 5 m (25 m2) subplots, to record other life forms such as shrubs. Furthermore, we laid five 2 m x 2 m (4 m2) subplots within the 25 $m^2$ to record herbs. The revised manuscript will also include information about the specific number of 5 m x 5 m subplots lay in each larger plot.

**Comments 9**: L169: Does it mean that voucher specimens per species were collected from all plots for unidentified/identified species?

**Response 9**: Thank you for your question. We apologize for the confusion caused by the sentence. In the revised manuscript, we were clarifying that voucher specimens were collected from all sampled plots, for both identified and unidentified species. This clarification will provide a more accurate understanding of the specimen collection process.

Environmental Data

Comment 10: L179: Won't the soil samples be more than 72? If they are collected from five different locations at each 1 m x 1 m subplot. Please clarify.

**Response 10**: Thank you for bringing up this point. We apologize for any confusion caused. To provide clarification, we revised the sentence as follows: "The soil samples collected for analysis were not more than 72. These 72 soil samples were collected from the 72 plots that were established for vegetation data collection. Within each main plot, soil samples were collected from five different locations using 1 m x 1 m (1 $m^2$) subplots, with one located in the middle and four at the corners of the plot. These samples were then mixed to produce a composite sample, from which one sample was taken for analysis. Therefore, the total number of soil samples was 72.

**Comment 11**: L181 air dried in natural conditions? Or a hot air oven?

**Response 11:** Thank you very much. It is air dried under natural conditions.

**Comment 12:** L184 The full form and abbreviations of TN and CEC should be mentioned in the introduction.

**Response 12:** Thanks for the hint. We write the full form abbreviation of TN and CEC in the introduction section based on reviewer comment.

**Comment 13:** L191-194: It will be easier for a reader to understand the methodology if we give more information than just citing previous papers. The scale looks interesting, but it will

be more apparent if the authors briefly describe the signs of disturbance that were considered for data collection. For example, the presence or absence of cattle dung? Proximity to the forests? Also, reading the discussion, human-caused disturbance is one of the major causes of species loss, so adding more information to this section would be worthwhile.

**Response 13:** Thank you for your valuable suggestion. Based on your comment, we have expanded the methodology section to provide additional information on the signs of disturbance considered in the data collection process. We now include details such as the presence or absence of cattle dung, proximity to the forests, and other relevant indicators of disturbance. By including this information, readers will have a clearer understanding of the factors assessed during data collection. Furthermore, we appreciate your insight regarding the impact of human-caused disturbance on species loss. In response to your suggestion, we have added more information on the effect of human disturbance on species loss in the discussion section. This addition enhances the overall discussion and provides a more comprehensive analysis of the study findings.

**Results**

**Comment 14**: Table 2: Having the full form of the floristic regions in the caption will be good. Also, were any of these species endemic? Will it be possible to add a row showing whether these are endemic? Ignore this comment if endemic species are listed elsewhere in another table.

**Response 14**: Thank you for pointing this out. We have represented new endemic species recorded in the Wollega floristic region in Table 2 by Asterix (*), which have not previously been recorded in the Wollega floristic region in the flora of Ethiopia and Eretria (Vol 1-7).

**Comment 15**: L266: Package information and its reference must be mentioned in the methods/analysis section, not the results section

**Response 15**: Thank you for pointing this out. We corrected based on reviewer comment

**Comment 16**: L370: Delete however and use while to connect both the sentences

**Response 16:** Thanks you, as suggested by the reviewer, we replaced it with "while".

**Comment 17:** L373 attitude?

**Response 17**: Thank you very much. We rewrite the sentence again to make it meaner and more complete as “Attitude explained 45.24% of the variance in community distribution compared to other environmental variables”.

**Comment 18**: L375 axes are 0.3504 and 0.2346 ….. follow the similar no of digits after the decimal points for the entire manuscript.

**Response 18*:*** Thanks for your suggestion. We have corrected it according to the reviewers' suggestions

**Comment 19**: Table 7 Altitude (m a s l) needs to be replaced with m.a.s.l. Also, the row title is in bold, while the rest of the tables are not; please follow the same formatting style.

Response 19: Thank you for pointing out the formatting issues in Table 7. We have made the necessary corrections according to your recommendations. We replaced "m a s l" with "m.a.s.l." to properly indicate altitude. Additionally, we have adjusted the formatting of the row title to match the non-bold style used in the other tables throughout the manuscript. These changes ensure consistency and improve readability. We have also reviewed the manuscript for any other discrepancies in formatting and made the necessary corrections to align with the established style.

**Comment 20**: The current file shows all the figures as Fig 1, which needs to be clarified. Is it the formatting issue? Kindly have a look at it and make the required changes.

Fig - Dendrogram of vegetation data from agglomerative hierarchical cluster analysis-

In this figure, the legend and the caption for the y and x axes need a larger font size; it is now difficult to read.

**Response 20**: Thanks for the tip. We agree to correct any reviewer comments on the figure throughout the current file. The entire figure does not represent Fig. 1, but all figures are stored in a folder. When you open it, you will get all the figures cited in the manuscript from Fig. 1 to Fig. 7. To avoid this error, we tried to arrange them in order as suggested by the reviewer. Regarding Fig. 4 Dendrogram of vegetation data from agglomerative hierarchical cluster analysis, we increased the font size of the legend and labels for the x and y axes based on the reviewer's suggestion in the final figure submission

**Comment 21**: Fig- Regression analysis along altitude, slope, TN, and disturbance.. Species richness ----- For this figure, the significance can be mentioned

at a similar location for all figures, towards the bottom right or top right. That will make the figure look clean and easy to read. Furthermore, please follow similar width and breadth sizes for all the figures.

**Response 21**: We agreed and adopted the reviewer's suggested comment

**Comment 22**: Fig 1. Influence of environmental variables on plant community types- here, please mention the plant community types.

**Response 22:** Thank you for the clarification. I apologize for the previous error. In Fig. 7, the diagram illustrates the influence of environmental variables on different plant community types:

Community type 1: *Maesa lanceolata-Ehretia cymosa*,

Community type 2: *Trichilia dregeana-Ficus vasta*

Community type 3: *Acacia abyssinica-Millettia ferruginea*

Community type 4: *Combertum collinum-Bersama abyssinica*

Community type 5: *Terminalia macroptera-Piliostigma thonninigii*
