# Supplementary material for: Topography and soil variables drive the plant community distribution pattern and species richness in the Arjo-Diga forest in western Ethiopia
Source: PLoS One. 2024 Aug 6;19(8):e0307888. doi: 10.1371/journal.pone.0307888 (PMC11302921; doi:10.1371/journal.pone.0307888)
Supplement: Attachment — Submitted filename: Comment for authors-Report.docx [file pone.0307888.s004.docx]

**Topography factors and soil variables drive the plant community distribution pattern and species richness in the Arjo Diga forest in western Ethiopia.**

The present MS is about a unique type of study made by four Ethiopian authors. They focus on community analysis along 106 environmental gradients and seek to assess the impact of environmental factors on plant diversity (i.e., richness, diversity and evenness). The MS seems interesting and worth-full to read as well as I enjoyed reading it. The data have been collected by authors by sampling which add more value on the MS. This sort data base may add credit to define species diversity and composition theory in the field of ecology.

Beside this I found some weak points. The major weakness of the study is not focused well as per the aims of the study. When one going for studies the MS he or she may confuse whether it is local scale study or macro scale study. The Arjo-Diga forests have elevations gradients ranging from 1,200 to 2,220 meters above sea level and I ask the authors to show the patterns along the elevation gradient as well?

However, there are some more flaws in the MS which needs to be improved before publication of the MS. I have jotted down some of the flaws in the MS as below:

**Abstract:**

The abstract is relatively longer and it confusing so I suggest to rephrase it.

**Introduction:**

The study has two types of data set like elevation gradient which is macro scale factor and soil, disturbance etc are local scale factors so the authors have to discuss about these factors how they influence the species patterns. So they have to demonstrate how these factors are associated in their study.

**Methodology**

The Arjo-Diga has elevations ranging from 1,200 to 2,220 meters above sea level which quite long gradient so I suggest honoring this gradient. Why authors use DCA and CCA have to elaborate why this data set suitable to use these techniques. They have not provided DCA and CCA diagram and their interpretation.

Others part of methodology is okey.

**Result:**

**The authors have identified 5 types of plant communities I still want to see these communities in the ordination diagram. So I want to ask to make CCA diagram how these five types of communities distributed?**

**Discussion**

I am skeptical with the presentation of discussion. The subtopics which are provided are vague so I want to see discussion under the specific subheading like: relationship between species diversity and climatic variables or relationship between species composition and environmental factors etc…. and how their finding is similar or difference with others such studies in the different scales in the Ehiopia and other parts of Globe.

**Conclusions**

**The conclusion rephrased as it is bit confusing!**

**In conclusion I found the MS is interesting but it still needs to be revised thoroughly.**
