# Supplementary material for: Topography and soil variables drive the plant community distribution pattern and species richness in the Arjo-Diga forest in western Ethiopia
Source: PLoS One. 2024 Aug 6;19(8):e0307888. doi: 10.1371/journal.pone.0307888 (PMC11302921; doi:10.1371/journal.pone.0307888)
Supplement: Attachment — Submitted filename: Reviwer two response.docx [file pone.0307888.s005.docx]

Manuscript ID PONE-D-23-07720

Reviewer 2 Response

**Subject:** **Resubmitting the revised Manuscript**

Thank you for giving us the opportunity to submit a revised draft of the manuscript “**Topography Factors and Soil Variables Control Plant Community Distribution Pattern and Species Richness in Arjo-Diga Forest, Western Ethiopia**” for publication in PLOS ONE journal. We appreciate the time and effort you put into providing feedback on our manuscript and are grateful for the insightful comments and valuable improvements made to our article. We accepted most of the reviewers' suggestions and comments. We have highlighted the changes within the manuscript below in a point-by-point response to the reviewers' comments and concerns. All page numbers refer to the revised manuscript file with tracked changes

Sincerely

Tariku Berihun (on behalf of all coauthors)

Dilla University

Email: [berihun.tariku@yahoo.com](mailto:berihun.tariku@yahoo.com)

Here is a point-by-point response to the reviewers comments and concerns.

**Comments from Reviewer 2**

**Abstract**

**Comment 1**: The abstract is relatively longer and it confusing so I suggest to rephrase it

**Response 1:** We agree. We rephrased it accordingly and compressed the words to 294 words. Therefore, we show the revised abstract with tracked change and without tracked change in the final draft manuscript

**Introduction**

**Comment 2**: The study has two types of data set like elevation gradient which is macro scale factor and soil, disturbance, etc., are local scale factors so the authors have to discuss about these factors how they influence the species patterns. So they have to demonstrate how these factors are associated in their study.

**Response 2:** Thank you for pointing this out. Based on your comment, we have discussed the effects of microclimate, disturbance and soil variables at local scale in Arjo Diga Forest as they all have significant impacts on plant community formation and biodiversity in Arjo Diga Forest. In addition, we corrected some of the grammatical errors in the final draft and corrected the manuscript in the revised manuscript with/without tracked change in the final draft of manuscript.

**Methodology**

**Comment 3**: The Arjo-Diga has elevations ranging from 1,200 to 2,220 meters above sea level which quite long gradient so I suggest honoring this gradient. Why authors use DCA and CCA have to elaborate why this data set suitable to use these techniques. They have not provided DCA and CCA diagram and their interpretation.

**Response 3**: Thank you for bringing the reviewer feedback to our attention. We agree that elevation differences can have significant impacts on plant community structure and species composition, and we welcome the reviewers' suggestions in this regard. Although we could not address the specific effects of altitude on plant community formation in the Arjo Diga forest in this manuscript, we recognize that this is an important factor that needs to be considered in future studies. We will definitely consider the reviewers' feedback in future proposals and manuscripts, and consider discussing the potential impact of altitude on plant communities in our study area. Additionally, if we have specific hypotheses or predictions about how altitude may affect plant communities, we will state them explicitly in our proposals or manuscripts. Thank you again for your helpful feedback.

Thank you for your feedback. We acknowledge that further explanation regarding our choice of ordination methods would be useful for readers. In our methods section, we briefly mentioned that we chose the CCA ordination method, but we did not provide sufficient justification for this choice in previous draft manuscript submitted. We have now revised our manuscript to include additional explanation for why we chose both DCA and CCA for our data analysis. After performing this preliminary analysis of vegetation data using DCA ordination, we found that the first axis length results in this study were 4.2 SD (standard deviation), confirming the heterogonous data set. For this reason, we decided to use the CCA ordination method, to show the relationship between environmental variables and plant community and plant diversity matrice.

**Result**

**Comment 4**: The authors have identified 5 types of plant communities I still want to see these communities in the ordination diagram. So I want to ask to make CCA diagram how these five types of communities distributed?

**Response 4**: Thank you for pointing this out. Using the CCA ordination method, we identified five distinct plant communities and generated an ordination diagram (Fig 5) to visualize their relationships with the environmental variables presented in Fig 5. In the list of figures, we present the identified plant communities.

**Discussion**
**Comment 5**: I am skeptical with the presentation of discussion. The subtopics which are provided are vague so I want to see discussion under the specific subheading like: relationship between species diversity and climatic variables or relationship between species composition and environmental factors etc.…. and how their findings are similar or different from those of other studies at different scales in Ehiopia and other parts of the globe.

**Response 5:** Thank you for pointing this out. We have added the reviewer’s suggestion based on the comment.

**Conclusions**

**Comment 6**: The conclusion rephrased as it is bit confusing! In conclusion, I found the MS is interesting but it still needs to be revised thoroughly.

**Response 6:** Thank you for pointing this out. We have revised and paraphrased this accordingly.
