# Supplementary material for: Topography and soil variables drive the plant community distribution pattern and species richness in the Arjo-Diga forest in western Ethiopia
Source: PLoS One. 2024 Aug 6;19(8):e0307888. doi: 10.1371/journal.pone.0307888 (PMC11302921; doi:10.1371/journal.pone.0307888)
Supplement: Attachment — Submitted filename: Comments_Sep_23.docx [file pone.0307888.s006.docx]

The study titled "Topography factors and soil variables drive the plant community distribution pattern and species richness in the Arjo Diga forest in western Ethiopia" aims to examine three critical points.

(1) the floristic composition and diversity of the Arjo Diga forest,

(2) the influence of environmental variables on the characteristics of plant diversity

(Richness, variety, and uniformity)

(3) Investigate the relationship between plant community types and environmental variables.

Such a kind of study, especially from the underrepresented areas, is essential and requires our attention. This manuscript has valuable finding which is vital for protecting existing plant species in the Arjo-Diga forest in Western Ethiopia.

The authors have put a reasonable effort into addressing reviewers' comments; however, there is a scope to make more corrections, which can be quickly addressed and will improve the manuscript's readability. The discussion sections require some improvements.

While revising, please be careful of typos; I have highlighted some of them. Also, edit the tables and figures following a similar format throughout the manuscript.

Here are my detailed comments.

**Abstract:**

L17-18, The plant family information is in between sentences, which discusses plant communities. The author can start with plant families, followed by another sentence about plant communities or vice versa.

Introduction:

The full form of TN and CEC needs to be included in the introduction.

L72-77 It would be nice if author could provide references for the following sentences

**Materials and Methods**

L113 Here, the author may introduce meters above sea level abbreviation as (m.a.s.l.), which can be used for the rest of the sections.

Throughout this section, authors can cite the references directly instead of using according to ……

**Ethical statement**

Although it is an important point, I am unsure if the ethical statement must be a part of the methodology section.

If it is only about the permits/permission the authors required for the sample collection, this will fit well in the acknowledgements.

**Methods of data collection**

L162: Use abbreviations (m.a.s.l.)

L165: Does it mean that out of 72 plots, a single plot size was 30 m x 30 m (900 m2) and inside each such plots authors also laid 5 m x 5 m (25 m2) and five 2 m x 2 m (4 m2) to record other life forms such as shrubs and herbs? If it is so, then it needs to be rewritten. Also, unlike herbs, how many 5 m x 5 m (25 m2) were laid in each plot needs to be clearly mentioned.

L169: Does it mean that voucher specimens per species were collected from all plots for unidentified/identified species?

**Environmental Data**

L179: Won’t the soil samples be more than 72? If it is collected from five different locations at each 1 m x 1 m subplot. Please clarify.

L181 airdried in natural conditions? Or a hot air oven?

L184 The full form and abbreviations of TN and CEC should be mentioned in the introduction.

L191-194: It will be easier for a reader to understand the methodology if we give more information than just citing previous papers. The scale looks interesting, but it will be more apparent if the authors briefly describe the signs of disturbance that were considered for data collection. For example, the presence or absence of cattle dung? Proximity to the forests?

Also, reading the discussion, human-caused disturbance is one of the major causes of species loss, so adding more information to this section would be worthwhile.

**Results**

Table 2: Having the full form of the floristics regions in the caption will be good.

Also, were any of these species endemic? Will it be possible to add a row showing whether these are endemic? Ignore this comment if endemic species are listed elsewhere in another table.

L266: Package information and its reference must be mentioned in the methods/analysis section, not the results section.

L370: Delete however and use while to connect both the sentences

L373 attitude?

L375 axes are 0.3504 and 0.2346 ….. follow the similar no of digits after

the decimal points for the entire manuscript.

Table 7 Altitude (m a s l) needs to be replaced with m.a.s.l.

Also, the row title is in bold, while the rest of the tables are not; please follow the same formatting style.

The current file shows all the figures as Fig 1, which needs to be clarified. Is it the formatting issue?

Kindly have a look at it and make the required changes.

Fig - Dendrogram of vegetation data from agglomerative hierarchical cluster analysis-

In this figure, the legend and the caption for the y and x axes need a larger font size; it is now difficult to read.

Fig- Regression analysis along altitude, slope, TN, and disturbance.. Species richness ----- For this figure, the significance can be mentioned

at a similar location for all figures, towards the bottom right or top right. That will make the figure look clean and easy to read. Furthermore, please follow similar width and breadth sizes for all the figures.

Fig 1. Influence of environmental variables on plant community types- here, please mention the plant community types.

**Discussion**

The first paragraph of the discussion should help the reader recollect your aims or the questions addressed, followed by the data used and the results found. Overall, the first paragraph of the discussion sections will lead to the remaining section of the discussion. I wonder if the comparison with the other forests at the beginning of the discussion makes any sense. It distracts the readers from the main questions.

Either remove it or reshuffle the flow of this section.
