# Supplementary material for: Topography and soil variables drive the plant community distribution pattern and species richness in the Arjo-Diga forest in western Ethiopia
Source: PLoS One. 2024 Aug 6;19(8):e0307888. doi: 10.1371/journal.pone.0307888 (PMC11302921; doi:10.1371/journal.pone.0307888)
Supplement: Attachment — Submitted filename: Reviwer #3 response.docx [file pone.0307888.s007.docx]

Manuscript ID PONE-D-23-07720

**Response to Reviewer 3**

**Subject:** **Resubmitting the revised Manuscript**

Thank you for giving us the opportunity to submit a revised draft of the manuscript “**Topography Factors and Soil Variables Control Plant Community Distribution Pattern and Species Richness in Arjo Diga Forest, Western Ethiopia**” for publication in PLOS ONE journal. We appreciate the time and effort you and the reviewers put into providing feedback on our manuscript and are grateful for the insightful comments and valuable improvements made to our article. We have accepted most of the reviewers' suggestions. We have highlighted the changes within the manuscript below a point-by-point response to the reviewers' comments and concerns. All page numbers refer to the revised manuscript file with tracked changes.

Sincerely

Tariku Berihun (on behalf of all coauthors)

Lecturer at Dilla University

Ph.D. fellow in Plant Biology and Biodiversity Management, Addis Ababa University

Email: [berihun.tariku@yahoo.com](mailto:berihun.tariku@yahoo.com)

**Comments from Reviewer 3**

Abstract

Comment1. L17-18, the plant family information is in between sentences, which discusses plant communities. The author can start with plant families, followed by another sentence about plant communities or vice versa

**Response 1:** Thanks for pointing this out. We reorder the sentence based on the reviewer's comment, i.e. we bring the set of plant families first and follow the plant communities.

Introduction

**Comment 2:** The full form of TN and CEC needs to be included in the introduction.

**Response 2:** Thank you. We agreed and wrote the full name of TN, CEC and SOC in the introductory section

**Comment 3:** L72-77 It would be nice if author could provide references for the following sentences

**Response** 3: Thank you, we agreed and cited references based on the reviewer's comment

Materials and Methods

**Comments 4:** L113 Here, the author may introduce meters above sea level abbreviation as (m.a.s.l.), which can be used for the rest of the sections and throughout this section; authors can cite the references directly instead of using according to

**Response 4:** Thank you based on the comments we introduce meters above sea level in the manuscript and then abbreviate it to m.a.s.l throughout the manuscript. Furthermore, as suggested by the reviewer, we agreed to cite references.

**Comment 5**: Ethical statement. Although it is an important point, I am unsure if the ethical statement must be a part of the methodology section. If it is only about the permits/permission the authors required for the sample collection, this will fit well in the acknowledgements.

**Response 5**: Thanks for the hint. We agreed to delete the ethics statement based on the reviewer's comment as the idea is to only reflect permission to collect soil and plant samples from the study area.

Methods of data collection

Comment 6: L162: Use abbreviations (m.a.s.l.)

**Response** 7: Thank you very much. We agree to the use of the abbreviation m.a.s.l. throughout the manuscript

Comments 7: L165: Does it mean that out of 72 plots, a single plot size was 30 m x 30 m (900 m2) and inside each such plots authors also laid 5 m x 5 m (25 m2) and five 2 m x 2 m (4 m2) to record other life forms such as shrubs and herbs? If it is so, then it needs to be rewritten. Also, unlike herbs, how many 5 m x 5 m (25 m2) were laid in each plot needs to be clearly mentioned.

**Response 7**: Thank you for pointing this out. As you said, the sentence contains an unclear idea, so we'll rephrase it again to make it clearer to the reader

**Comment 8:** L169: Does it mean that voucher specimens per species were collected from all plots for unidentified/identified species?

**Response 8**: It is not the case that voucher specimens per species were collected from all plots for unidentified/identified species. It is said to be a voucher specimen per plots were collected and identified using Flora of Ethiopia and Etruria(volume1-8)

Environmental Data

**Comment 9**: L179: Won’t the soil samples be more than 72? If it is collected from five different locations at each 1 m x 1 m subplot. Please clarify.

**Response 9:** Thanks for the tip. There are no more than 72. Because we collected soil samples from a total of 72 plots. Within each plot, we collected soil samples from five subplots measuring 1 m × 1 m. After collecting the soil samples from the five subplots, we combined them to create a single composite soil sample representing each plot. Therefore, there was one soil sample per plot, resulting in a total of 72 soil samples were collected.

**Comment 10:** L181 air dried in natural conditions? Or a hot air oven?

**Response 10:** Thank you very much. It is air dried under natural conditions.

**Comment 11:** L184 The full form and abbreviations of TN and CEC should be mentioned in the introduction.

**Response 11:** Thanks for the hint. We agree and write the abbreviation of TN and CEC in the introduction section.

**Comment 12:** L191-194: It will be easier for a reader to understand the methodology if we give more information than just citing previous papers. The scale looks interesting, but it will be more apparent if the authors briefly describe the signs of disturbance that were considered for data collection. For example, the presence or absence of cattle dungs? Proximity to the forests? Also, reading the discussion, human-caused disturbance is one of the major causes of species loss, so adding more information to this section would be worthwhile.

**Response 12**: We expanded the methodology and provided additional information on the signs of disturbance considered in the data collection process, namely the proximity to the forests and the absence/presence of livestock dungs.

**Results**

**Comment 12**: Table 2: Having the full form of the floristic regions in the caption will be good. Also, were any of these species endemic? Will it be possible to add a row showing whether these are endemic? Ignore this comment if endemic species are listed elsewhere in another table.

**Response 12**: Thank you for pointing this out. We have represented new endemic species recorded in the Wollega floristic region in Table 2 by Asterix (*), which have not previously been recorded in the Wollega floristic region in the flora of Ethiopia and Eretria.

**Comment 13**: L266: Package information and its reference must be mentioned in the methods/analysis section, not the results section

**Response 13**: Thank you for pointing this out. We corrected based on reviewer comment

**Comment 14**: L370: Delete however and use while to connect both the sentences

**Response 14:** Thanks you, as suggested by the reviewer, we replaced it with "while".

**Comment 15:** L373 attitude?

**Response 15**: Thank you very much. We rewrite the sentence again to make it meaner and more complete as “Attitude explained 45.24% of the variance in community distribution compared to other environmental variables”.

**Comment 16**: L375 axes are 0.3504 and 0.2346 ….. follow the similar no of digits after

the decimal points for the entire manuscript.

**Response 16*:*** Thanks for your suggestion. We have corrected it according to the reviewers' suggestions

Comment **17**: Table 7 Altitude (m a s l) needs to be replaced with m.a.s.l. Also, the row title is in bold, while the rest of the tables are not; please follow the same formatting style.

Response 17: Thanks for the tip. We have corrected according to the reviewer's recommendations. The bold row title table has been corrected as non-bold. For the rest of the other, we follow the same format throughout the manuscript and correct what disagrees with others.

**Comment 18**: The current file shows all the figures as Fig 1, which needs to be clarified. Is it the formatting issue? Kindly have a look at it and make the required changes.

Fig - Dendrogram of vegetation data from agglomerative hierarchical cluster analysis-

In this figure, the legend and the caption for the y and x axes need a larger font size; it is now difficult to read.

**Response 18**: Thanks for the tip. We agree to correct any reviewer comments on the figure throughout the current file. The entire figure does not represent Fig. 1, but all figures are stored in a folder. When you open it, you will get all the figures cited in the manuscript from Fig. 1 to Fig. 7. To avoid this error, we tried to arrange them in order as suggested by the reviewer. Regarding Fig. 4 Dendrogram of vegetation data from agglomerative hierarchical cluster analysis, we increased the font size of the legend and labels for the x and y axes based on the reviewer's suggestion in the final figure submission

**Comment 19**: Fig- Regression analysis along altitude, slope, TN, and disturbance.. Species richness ----- For this figure, the significance can be mentioned

at a similar location for all figures, towards the bottom right or top right. That will make the figure look clean and easy to read. Furthermore, please follow similar width and breadth sizes for all the figures.

**Response 19**: We agreed and adopted the reviewer's suggested comment

**Comment 20**: Fig 1. Influence of environmental variables on plant community types- here, please mention the plant community types.

**Response 20:** Thank you for pointing this out. It is Fig. 7, not Fig. 1 as you comment. It is Fig. 7. Influence of environmental variables on plant community types (Community 1 = *Maesa lanceolata-Ehretia cymosa*, Community 2 = *Trichilia dregeana-Ficus vasta*, Community 3 = *Acacia abyssinica-Millettia ferruginea*, Community 4 = *Combertum collinum -Bersama abyssinica* and Community 5 = *Terminalia macroptera-Piliostigma thonninigii*).
