# Supplementary material for: Topography and soil variables drive the plant community distribution pattern and species richness in the Arjo-Diga forest in western Ethiopia
Source: PLoS One. 2024 Aug 6;19(8):e0307888. doi: 10.1371/journal.pone.0307888 (PMC11302921; doi:10.1371/journal.pone.0307888)
Supplement: Attachment — Submitted filename: Reviwer #3 response.docx [file pone.0307888.s008.docx]

Manuscript ID PONE-D-23-07720

**Response to Reviewer 3**

**Subject:** **Resubmitting the revised Manuscript**

Thank you for giving us the opportunity to submit a revised draft of the manuscript “**Topography Factors and Soil Variables Control Plant Community Distribution Pattern and Species Richness in Arjo Diga Forest, Western Ethiopia**” for publication in PLOS ONE journal. We sincerely appreciate the time and effort you and the reviewers have dedicated to providing feedback on our manuscript. We are grateful for the insightful comments and valuable improvements made to our article. We have accepted the majority of the editor's suggestions and have also corrected the grammar errors, tables and figures formatting problem according to journal

Sincerely

Tariku Berihun (on behalf of all coauthors)

Lecturer at Dilla University

Ph.D. fellow in Plant Biology and Biodiversity Management, Addis Ababa University

Email: [berihun.tariku@yahoo.com](mailto:berihun.tariku@yahoo.com)

**Comments from Reviewer 3**

Abstract

**Comment 1**: L17-18, the plant family information is in between sentences, which discusses plant communities. The author can start with plant families, followed by another sentence about plant communities or vice versa

**Response 1**: Thank you for your suggestion. We agree with your point and have revised the manuscript accordingly. In the revised version, we have started with a sentence discussing plant families, followed by a sentence addressing plant communities. This reorganization improves the flow and clarity of the information.

Introduction

**Comment 2:** The full form of TN and CEC needs to be included in the introduction.

**Response 2:** Thank you. We wrote the full form of TN, and CEC based on a reviewer’s in the introductory section

**Comment 3:** L72-77 It would be nice if author could provide references for the following sentences

**Response** 3: Thank you. We provide and cite references based on a reviewer's comment

Materials and Methods

**Comments 4:** L113 Here, the author may introduce meters above sea level abbreviation as (m.a.s.l.), which can be used for the rest of the sections and throughout this section; authors can cite the references directly instead of using according to

**Response 4:** Thank you for this valuable suggestion. We have incorporated the suggested abbreviation "m.a.s.l." (meters above sea level) in the manuscript. This abbreviation was used consistently throughout the rest of the sections to provide a concise and standardized representation of elevation measurements.

**Comment 5:** Throughout this section, authors can cite the references directly instead of using according to …

**Response 5**: Thank you for bringing this to our attention. We have made revisions to this section by directly citing the relevant references instead of using the phrase "according to." The direct citation of appropriate sources aims to enhance the clarity and accuracy of the information presented in the manuscript.

**Comment 6**: Line 147-155.ethical statement.

Although it is an important point, I am unsure if the ethical statement must be a part of the methodology section. If it is only about the permits/permission the authors required for the sample collection, this will fit well in the acknowledgements.

**Response 6**: Thank you. We carefully considered the placement of the ethical statement in the manuscript. After consideration, we agree that it would be more appropriate to include this information in the Acknowledgments section rather than the Methodology section, as the ethical statement relates to the permits/permissions for authors to collect samples from the study area. On this basis, we agreed to move the ethical statement to the Acknowledgments section based on the reviewer's comment, as the idea is to reflect the permits/permissions that the authors have for the collection of plant and soil samples from the study area.

Methods of data collection

**Comment 7**: L162: Use abbreviations (m.a.s.l.)

**Response 7**: Thank you very much. As suggested, the abbreviation m.a.s.l. were used throughout our manuscript.

Comment 8: L165: Does it mean that out of 72 plots, a single plot size was 30 m x 30 m (900 m2) and inside each such plot authors also laid 5 m x 5 m (25 m2) and five 2 m x 2 m (4 m2) to record other life forms such as shrubs and herbs? If it is so, then it needs to be rewritten. Also, unlike herbs, how many 5 m x 5 m (25 m2) were laid in each plot needs to be clearly mentioned

**Response 8**: Thank you for your comment and clarification. We apologize for any confusion caused by the sentence. To provide a clear explanation, we have revised the sentence as follows: yes, out of the 72 plots, each plot had a size of 30 m x 30 m (900 m2). Additionally, within each large plot, we laid five smaller plots, including 5 m x 5 m (25 m2) subplots, to record other life forms such as shrubs. Furthermore, we laid five 2 m x 2 m (4 m2) subplots within the 25 m^2^ to record herbs. The revised manuscript will also include information about the specific number of 5 m x 5 m subplots lay in each larger plot.

**Comments 9**: L169: Does it mean that voucher specimens per species were collected from all plots for unidentified/identified species?

**Response 9**: Thank you for your question. We apologize for the confusion caused by the sentence. In the revised manuscript, we were clarifying that voucher specimens were collected from all sampled plots, for both identified and unidentified species. This clarification will provide a more accurate understanding of the specimen collection process.

Environmental Data

Comment 10: L179: Won’t the soil samples be more than 72? If they are collected from five different locations at each 1 m x 1 m subplot. Please clarify.

**Response 10**: Thank you for bringing up this point. We apologize for any confusion caused. To provide clarification, we revised the sentence as follows: "The soil samples collected for analysis were not more than 72. These 72 soil samples were collected from the 72 plots that were established for vegetation data collection. Within each main plot, soil samples were collected from five different locations using 1 m x 1 m (1 m²) subplots, with one located in the middle and four at the corners of the plot. These samples were then mixed to produce a composite sample, from which one sample was taken for analysis. Therefore, the total number of soil samples was 72.

**Comment 11**: L181 air dried in natural conditions? Or a hot air oven?

**Response 11:** Thank you very much. It is air dried under natural conditions.

**Comment 12:** L184 The full form and abbreviations of TN and CEC should be mentioned in the introduction.

**Response 12:** Thanks for the hint. We write the full form abbreviation of TN and CEC in the introduction section based on reviewer comment.

**Comment 13:** L191-194: It will be easier for a reader to understand the methodology if we give more information than just citing previous papers. The scale looks interesting, but it will be more apparent if the authors briefly describe the signs of disturbance that were considered for data collection. For example, the presence or absence of cattle dung? Proximity to the forests? Also, reading the discussion, human-caused disturbance is one of the major causes of species loss, so adding more information to this section would be worthwhile.

**Response 13:** Thank you for your valuable suggestion. Based on your comment, we have expanded the methodology section to provide additional information on the signs of disturbance considered in the data collection process. We now include details such as the presence or absence of cattle dung, proximity to the forests, and other relevant indicators of disturbance. By including this information, readers will have a clearer understanding of the factors assessed during data collection. Furthermore, we appreciate your insight regarding the impact of human-caused disturbance on species loss. In response to your suggestion, we have added more information on the effect of human disturbance on species loss in the discussion section. This addition enhances the overall discussion and provides a more comprehensive analysis of the study findings.

**Results**

**Comment 14**: Table 2: Having the full form of the floristic regions in the caption will be good. Also, were any of these species endemic? Will it be possible to add a row showing whether these are endemic? Ignore this comment if endemic species are listed elsewhere in another table.

**Response 14**: Thank you for pointing this out. We have represented new endemic species recorded in the Wollega floristic region in Table 2 by Asterix (*), which have not previously been recorded in the Wollega floristic region in the flora of Ethiopia and Eretria (Vol 1-7).

**Comment 15**: L266: Package information and its reference must be mentioned in the methods/analysis section, not the results section

**Response 15**: Thank you for pointing this out. We corrected based on reviewer comment

**Comment 16**: L370: Delete however and use while to connect both the sentences

**Response 16:** Thanks you, as suggested by the reviewer, we replaced it with "while".

**Comment 17:** L373 attitude?

**Response 17**: Thank you very much. We rewrite the sentence again to make it meaner and more complete as “Attitude explained 45.24% of the variance in community distribution compared to other environmental variables”.

**Comment 18**: L375 axes are 0.3504 and 0.2346 ….. follow the similar no of digits after

the decimal points for the entire manuscript.

**Response 18*:*** Thanks for your suggestion. We have corrected it according to the reviewers' suggestions

**Comment 19**: Table 7 Altitude (m a s l) needs to be replaced with m.a.s.l. Also, the row title is in bold, while the rest of the tables are not; please follow the same formatting style.

Response 19: Thank you for pointing out the formatting issues in Table 7. We have made the necessary corrections according to your recommendations. We replaced "m a s l" with "m.a.s.l." to properly indicate altitude. Additionally, we have adjusted the formatting of the row title to match the non-bold style used in the other tables throughout the manuscript. These changes ensure consistency and improve readability. We have also reviewed the manuscript for any other discrepancies in formatting and made the necessary corrections to align with the established style.

**Comment 20**: The current file shows all the figures as Fig 1, which needs to be clarified. Is it the formatting issue? Kindly have a look at it and make the required changes.

Fig - Dendrogram of vegetation data from agglomerative hierarchical cluster analysis-

In this figure, the legend and the caption for the y and x axes need a larger font size; it is now difficult to read.

**Response 20**: Thanks for the tip. We agree to correct any reviewer comments on the figure throughout the current file. The entire figure does not represent Fig. 1, but all figures are stored in a folder. When you open it, you will get all the figures cited in the manuscript from Fig. 1 to Fig. 7. To avoid this error, we tried to arrange them in order as suggested by the reviewer. Regarding Fig. 4 Dendrogram of vegetation data from agglomerative hierarchical cluster analysis, we increased the font size of the legend and labels for the x and y axes based on the reviewer's suggestion in the final figure submission

**Comment 21**: Fig- Regression analysis along altitude, slope, TN, and disturbance.. Species richness ----- For this figure, the significance can be mentioned

at a similar location for all figures, towards the bottom right or top right. That will make the figure look clean and easy to read. Furthermore, please follow similar width and breadth sizes for all the figures.

**Response 21**: We agreed and adopted the reviewer's suggested comment

**Comment 22**: Fig 1. Influence of environmental variables on plant community types- here, please mention the plant community types.

**Response 22:** Thank you for the clarification. I apologize for the previous error. In Fig. 7, the diagram illustrates the influence of environmental variables on different plant community types:

Community type 1: *Maesa lanceolata-Ehretia cymosa*,

Community type 2: *Trichilia dregeana-Ficus vasta*

Community type 3: *Acacia abyssinica-Millettia ferruginea*

Community type 4: *Combertum collinum-Bersama abyssinica*

Community type 5: *Terminalia macroptera-Piliostigma thonninigii*
