# Supplementary material for: Topography and soil variables drive the plant community distribution pattern and species richness in the Arjo-Diga forest in western Ethiopia
Source: PLoS One. 2024 Aug 6;19(8):e0307888. doi: 10.1371/journal.pone.0307888 (PMC11302921; doi:10.1371/journal.pone.0307888)
Supplement: Attachment — Submitted filename: Tambat_Suggestions_PONE-D-23-07720R4.docx [file pone.0307888.s010.docx]

Dear Editor

Greeting from Tambat, India

I am writing with regards to the article “Topography factors and soil variables drive the plant community distribution pattern and species richness in the Arjo Diga forest in western Ethiopia”

The study is fine, in my view this article may be revised thoroughly and resubmitted.

Specific comments

1. There are gaps in readability / connecting sentences hence greater scope for improvement. Several places sentences are repeated.
2. Introduction, need for the study and purpose needs to clearly defined
3. Material methods – Soil and Geology-Specific value for the CEC, pH could be given

Plants scientific names - must include Author name also.

Vegetation data collection- more clarity required on the sampling – figure may be given for better clarity. Data collection on environmental variable and Disturbance is not clear hence greater details may be provided. Disturbance- scoring and formula may be given to indicate how the specific value/score was assigned. Plant data analysis- stepwise analysis could be given so to have better understanding.

4. Results- newly discovered species and endemism- it will be nice to specify when the species was discovered and endemic, RET status was assigned (if different years then earliest discovered species year).
5. Results- Parameter (Eg Altitude, Total nitrogen, pH etc) and vegetation recorded can be given and comparison between communities available and analysed groups can be made.
6. Regression analysis / graphs – N number may be given, relationship can be made more clearly visible. Table 8 – what does it convey not clear details may be given.
7. Discussion may be elaborated
8. Conclusion can be re-written in the view of aim of the study.

Few questions:

1. What is the need to mention about new discoveries and endemicity?
2. Dominant species can be analysed through using RF, RD, IVI, Density, Volume, etc why these analysis
3. Similarity can be tested and compared across the environmental and or soil parameters ?
4. Grouping also has variation with regards to altitudinal range (some are 100 m and some are > 300 m)
5. One side comparing community (Groups) and other side comparing only richness, diversity across the environmental variable, what is the purpose and how to conclude?
